# SECIMTools: a suite of metabolomics data analysis tools

**DOI:** 10.1186/s12859-018-2134-1

**Published:** 2018-04-20

**Authors:** Alexander S. Kirpich, Miguel Ibarra, Oleksandr Moskalenko, Justin M. Fear, Joseph Gerken, Xinlei Mi, Ali Ashrafi, Alison M. Morse, Lauren M. McIntyre

**Affiliations:** 1 Southeast Center for Integrated Metabolomics (SECIM), University of Florida, Gainesville, FL 32611 USA; 2 University of Florida Informatics Institute, University of Florida, Gainesville, FL 32611 USA; 3 University of Florida Genetics Institute, University of Florida, Gainesville, FL 32611 USA; 4 Department of Molecular Genetics and Microbiology, University of Florida, Gainesville, FL 32611 USA; 5 University of Florida Research Computing, University of Florida, Gainesville, FL 32611 USA; 6 National Institute of Health, Washington, DC USA; 7 Department of Biostatistics, University of Florida, Gainesville, FL 32611 USA

## Abstract

**Background:**

Metabolomics has the promise to transform the area of personalized medicine with the rapid development of high throughput technology for untargeted analysis of metabolites. Open access, easy to use, analytic tools that are broadly accessible to the biological community need to be developed. While technology used in metabolomics varies, most metabolomics studies have a set of features identified. Galaxy is an open access platform that enables scientists at all levels to interact with big data. Galaxy promotes reproducibility by saving histories and enabling the sharing workflows among scientists.

**Results:**

SECIMTools (SouthEast Center for Integrated Metabolomics) is a set of Python applications that are available both as standalone tools and wrapped for use in Galaxy. The suite includes a comprehensive set of quality control metrics (retention time window evaluation and various peak evaluation tools), visualization techniques (hierarchical cluster heatmap, principal component analysis, modular modularity clustering), basic statistical analysis methods (partial least squares - discriminant analysis, analysis of variance, *t*-test, Kruskal-Wallis non-parametric test), advanced classification methods (random forest, support vector machines), and advanced variable selection tools (least absolute shrinkage and selection operator LASSO and Elastic Net).

**Conclusions:**

SECIMTools leverages the Galaxy platform and enables integrated workflows for metabolomics data analysis made from building blocks designed for easy use and interpretability. Standard data formats and a set of utilities allow arbitrary linkages between tools to encourage novel workflow designs. The Galaxy framework enables future data integration for metabolomics studies with other omics data.

**Electronic supplementary material:**

The online version of this article (10.1186/s12859-018-2134-1) contains supplementary material, which is available to authorized users.

## Background

Metabolomics is the large-scale identification and quantification of small molecules across multiple biological samples [1]. These small molecules, predominantly less than 1500 Da, include primary and secondary metabolites, hormones, and metabolic intermediates. Their analyses can reveal the chemical processes and cellular physiology occurring within a biological sample at a given time [2].

The vast diversity of biochemical reactions and experimental goals requires the implementation of different technology in metabolic profiling. Unlike gene expression profiling, there is no single platform or technology that can capture the entire metabolome. Like expression profiling, the standard workflow can be divided into sample preparation, data acquisition, data preprocessing, and data analysis. Platform development is a focus of metabolomics research [3] with platform specific sample preparation and data acquisition. Each of technology has unique properties and different methods that are used to convert raw data into potential metabolites [4]. Thus, data preprocessing is platform specific. The feature identification, or “peak picking” is particular to the technological properties of each platform, and has its own literature [5, 6].

Targeted metabolite quantification is common in everything from drug tests [7, 8] and cholesterol measurement [9] to industrial scale safety testing [10]. The success of such measurements of metabolism has led to interest in unbiased assays of the metabolome. Untargeted metabolomics is a relatively new field, and there are few tools developed for the analysis of these data. Features are the starting point for MetaboAnalyst, a standalone, and state of the art, tool developed at the University of Alberta for data pre-processing and statistical analysis [11]. MetaboAnalyst has a user-friendly interface with a set of point and click menu options that guide the user through the analysis.

Galaxy is a web based platform with an intuitive interface [12]. Galaxy is an ecosystem for the development of analytical tools. As such, it is not focused on any single technology but rather enables analysis across a broad range of technological platforms. The platform is open source, allowing developers to share code and work in concert. Workflows can be created using a user-friendly workflow visualization tool and executed by scientists without a programming background. Workflows can be saved and shared, allowing reproducible data analysis. Each step is documented in the history. Histories can be saved, shared, and converted into new workflows. Using the Galaxy platform, developers can make tools accessible to a broad audience. Scientists can customize and integrate different tools from a variety of programmers into a single workflow. Galaxy can be installed on a server or on a local machine, and it can take advantage of a cluster environments.

Recently, two Galaxy toolkits for metabolomics data analysis have been developed. Galaxy-M was introduced for peak-picking/feature identification and data pre-processing [13]. Workflow4Metabolomics is a framework that focuses on feature annotation and includes analysis of variance (ANOVA), principal component analysis (PCA), and hierarchical clustering analysis [14]. SECIMTools (SouthEast Center for Integrated Metabolomics) are designed to complement both efforts. SECIMTools start with features and the suite enables comprehensive quality assessment and sophisticated statistical analysis. The data format for input to individual tools is similar among all three Galaxy platforms. There is some overlap among the tools. For example single factor fixed ANOVA analysis and PCA are included in all three. However, the emphasis of each suite is distinct and SECIMTools includes several new QC tools as well as variable selection tools not available in their toolkits, or in Galaxy.

While some of the components in SECIMTools are focused solely on metabolomics data, others can be applied more broadly to omics data. Most of the QC and statistical tools are new to the Galaxy platform. New functionality includes: blank feature filtering [15]; retention time diagnostics; run order evaluation; advanced imputation methods [16–19]; LASSO [20]; Elastic Net [21]; random forests [22]; support vector machine [23–25]; and Modulated Modularity Clustering [26]. To connect the tools into workflows utilities and graphing tools have been developed. The current set of tools is a balance between having familiar existing tools reprogrammed in the SECIMTools color palate and to enable a very straightforward workflow construction, with the addition of new to Galaxy features (e.g. Elastic Net) and new metabolomics specific QC tools. SECIMTools is an integrated suite for sophisticated statistical analysis of metabolomics data. Many of the tools can be used more broadly for analysis of omics data.

## Implementation

SECIMTools has standardized tool inputs and outputs and allows scientists to develop of novel workflows. SECIMTools is accompanied by a comprehensive user guide (Additional file 1), a set of workflow examples and example datasets. The user guide provides detailed descriptions of expected inputs, functionality, and outputs. Additional file 2 has examples to illustrate graphical output from each tool. SECIMTools is open source, the code, is available on GitHub using the MIT license [27].

**Fig. 1:**
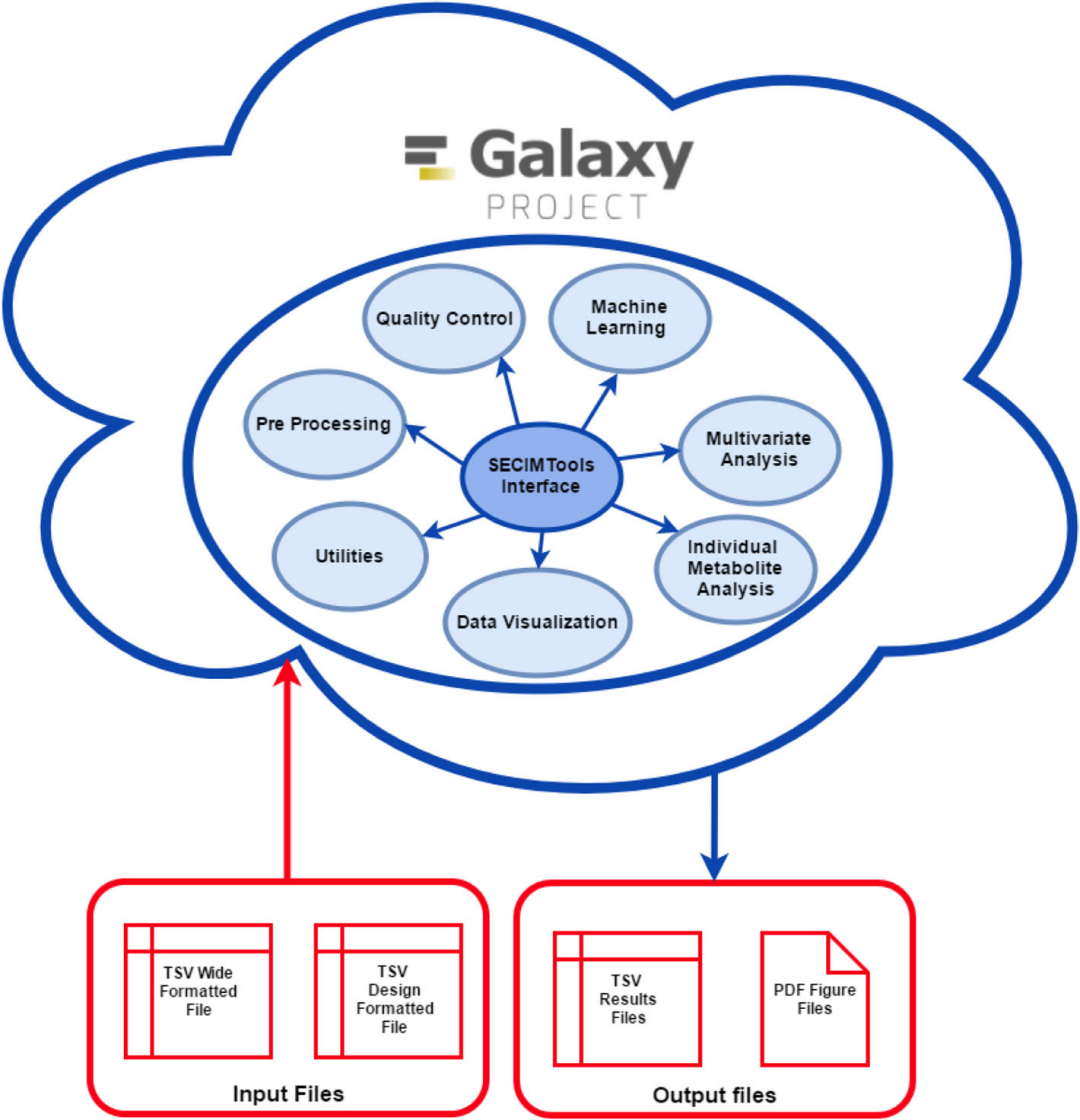
The SECIMTools structure: The outside cloud represents the Galaxy environment. The inside circle represents the set of SECIMTools. A common data handling and input/output architecture for all the SECIMTools, enables the development of analytical workflows without continual data manipulation and reformatting. Most tools expects two files describing the data, one giving information about each sample and the experimental design (design formatted file), and one giving the estimated feature intensities for each sample (wide formatted files). Galaxy expects files in a tab separated format (tsv). Tools that convert to tsv format from other common formats exist as a part of Galaxy. The output files are result files (e.g. -values from an ANOVA) and figures (e.g. Scatterplots). The result tables are returned to the user in a Galaxy compatible tsv format. Plots have a common color scheme with a customizable color palate that will apply the same coloring scheme to all results. A detailed description of the data formats is given in the user guide

**Fig. 2:**
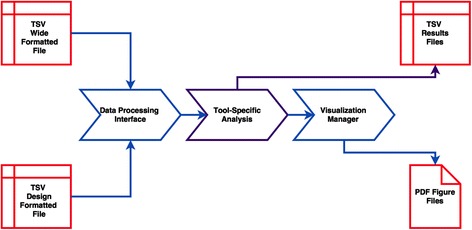
Individual tool structure: The input data have the same standard format, and a common visualization manager which generates outputs in a standard format

SECIMTools consists of four main types of tools: data pre-processing, quality control (QC), data analysis, and utilities (Fig. 1). The individual tools are organized using a modular structure. The input data, data processing interface, visualization manager and outputs are standardized (Fig. 2). Metabolomics Workbench is an online repository for metabolomics data as is Metabolites. Both of these databases use a file format with samples as columns and features as rows. The files available in both public repositories can be imported into Galaxy and used in SECIMTools. Scientists can also upload their own data into Galaxy and Galaxy can be installed on a local workstation. SECIMTools uses two main input files. The experimental data are represented in a data table in which samples are in columns and metabolomics features (or genes) are in rows. The table should contain feature identifiers that are unique for each row. This format is referred to as a “wide formatted file” or “wide format dataset”. Missing values can be imputed or features with missing values can be removed. The design file is used to relate sample data with sample characteristics (e.g. treatment group, batch ID, sample weight, run order). In the Metabolomics workbench [28] the design file is referred to as the meta-data file. Readers are referred to the user guide (Additional file 1) for more details on the input formats.

### Individual tool structure

#### Data pre-processing

Metabolomics specific data pre-processing tools *Blank Feature Filtering (BFF) Flags* and *Threshold Based Flags* are included in SECIMTools. The *Threshold Based Flags* tool identifies features below a user specified threshold in more than 50% of samples within a given group. The *Blank Feature Filtering (BFF)* tool calculates a limit of detection based upon values for a feature [15].

Additional omics data pre-processing tools are: *Data Normalization and Re-Scaling, Imputation,* and *Log/G-log Transformation*. The *Log/G-log Transformation* tool was developed to perform a log or a generalized log (g-log) [29] transformation with different bases (2, 10 and natural). The *Data Normalization and Re-Scaling* tool includes the sample mean, median and sum of all features as scaling factors used to divide by the selected sample specific factor. Data centering, autoscaling, Pareto scaling, range scaling, level scaling, and variable stability (VAST) scaling are available [30]. Normalizations for raw NMR data such as probabilistic quotient normalization (PQN) are available in other tools such as Galaxy-M [13].

The *Imputation* tool includes the use of the group mean or group median in place of any missing values as well as K-nearest neighbor (KNN) [16, 17] and stochastic imputation [19]. KNN imputation method is an advanced, sensitive and robust method [16, 17]. KNN is deterministic and produces the same result for a given dataset. In contrast, stochastic imputation provides an estimate based on a model that includes random noise and will produce a different result every time the tool is invoked. The parameters of the distribution (Poisson or Normal) are estimated from the available data, and missing values are drawn from a distribution where the parameters match the values estimated from the non-missing data. The KNN python code is distributed under the GNU license [17]. KNN should be considered carefully before use [31, 32]**.**

#### Quality control (QC) analysis tools

Quality control (QC) is an important and often overlooked part of an analysis workflow. The QC tools in the suite can be used not only for metabolomics but also for other types of -omics data. The tools presented here are not in place of the quality metrics that are used during data acquisition and initial processing to generate quantified features. The focus of the QC tools is to identify potential feature artefacts, and/or aberrant samples.

SECIMTools includes several unique QC elements as well as standard QC approaches. Inspection and removing (filtering) of features and samples is a critical part of any “omics” data QC analysis. Each QC tool creates a set of 0/1 indicator variables (flags) that the user can interpret using graphical output and determine which samples or features (if any) to filter from further analysis. The decision to filter features from further analysis is left to the discretion of the individual scientist and each tool outputs indicators that may or may not be used for downstream filtering. A separate tool that allows filtering of features and samples is part of the utilities suite. Samples can also be filtered using design files.

Metabolomics specific QC tools are *Retention Time (RT) Flags* and *Run Order Regression (ROR).* The *Retention Time (RT)* Flags tool is specific for mass spectroscopy (MS) analysis. Variation in retention time can indicate technical problems in the injection, issues in feature identification (e.g. alignment) and chromatographic artifacts. The *Retention Time (RT)* tool uses two criteria: the tool identifies features with the largest coefficients of variation by percentile using a threshold (10% by default) and features that exceed an absolute threshold. Flags are saved and output. AN example of the Retention Time tool graphic output is provided in the Additional file 2: Figure S1.

*Run Order Regression* (*ROR*) is designed to investigate potential problems due to carry over effects. In other words, intensities of a feature should not be associated with run order. The *ROR* tool uses linear regression to evaluate the relationship between feature intensity and the run order. In a feature with no carry over effects there should be no association between the run order and the estimated feature intensity, a slope of 0. Features are identified if there is an indication that regression slope is different from 0 for nominal type I error *α* = 0.05 or *α* = 0.01. Regression plots and a summary file with flags are produced. The example of the Run Order Regression tool graphic output is provided in the Additional file 2: Figure S2.

General QC tools that can be applied for any types of –omics data are: *Bland-Altman (BA), Coefficient of Variation (CV) Flags, Distribution of Features across Samples, Distribution of Features within Samples, Magnitude Difference,* and *Standardized Euclidean Distance (SED).*

The *Bland-Altman (BA) plot* [33] provides a visualization of pairwise agreement. Initially developed to compare measurements of the same samples, it has been adapted to compare replicates of the same type in microarray data [34] and for RNA-seq [35]. The difference between features from two samples is the value on the y-axis and the mean of the features is the value on the x-axis. A “good” Bland-Altman plot will have a cigar shape centered on a difference of 0. The tool can be used on a set of technical reps for pooled samples, where no differences among the pools are expected. Not all metabolomics experiments include such pools. Features with low repeatability will appear as distinct points separate from the main cluster of points. The *Bland-Altman (BA)* tool deploys a novel approach to automatically identify problematic features. The BA tool quantifies the relationship between the difference and the mean using a linear regression fit. A “good” plot has with the expectation of a slope equal to 0. The estimated slope, is reported on the plots. The features with large standardized residuals and leverage statistics (DFFITS and Cook’s D) [36–38] are identified. On the plots, those features identified by at least one of the three methods are colored in red. In the absence of technical replicates for pooled samples, comparisons within a group can be made, and corresponding unstable features identified. The examples of the Bland-Altman tool graphic outputs are provided in the Additional file 2: Figures S3 and S4.

The *Coefficient of Variation (CV)* is a common method for identification of measurements with particularly large variance relative to the mean [39]. Large CV values can indicate problems with specific features. By default, the *Coefficient of Variation (CV) Flags* tool identifies the top X% of features, with the user specifying X (default value is 10%). The example of the Coefficient of Variation tool graphic output is provided in the Additional file 2: Figure S5.

Within a treatment group feature intensities may be expected to be the same order of magnitude. The *Magnitude Difference Flags* tool counts the number of digits prior to the decimal point for each group and generates a report. The goal is to identify the differences in the order of magnitude. Large differences in magnitude for many features for an individual sample may be caused by a variety of technical problems. Large differences across samples for a feature may indicate and chromatographic artifact. The output for the tool includes a count of the number of order of magnitude differences for features with the most differences for a user defined number of features (default is 50). For each sample, the number of features with an order of magnitude difference is counted and a plot of all the samples is generated. Output files for each feature and each sample are created. The example of the Magnitude Difference Flags tool graphic output is provided in the Additional file 2: Figure S6.

*Distribution of Features across Samples* provides boxplots for 50 random features. Density plots for samples that show the distribution across features are also displayed. *Distribution of Features within Samples* provides the distribution boxplots and density plots for all features within each sample. The two tools are designed to identify consistent anomalies. The example of the Distribution of Features across Samples tool graphic output is provided in the Additional file 2: Figure S7. The examples of the Distribution of Features within Samples tool graphic outputs are provided in the Additional file 2: Figures S8 and S9.

The *Standardized Euclidean Distance (SED)* tool can be used to compare samples within a group. The group center is calculated as the mean of each feature across samples in the group.

$$ SED\left(x,y\right)=\sqrt{\sum \limits_i^n\frac{{\left({x}_i-{y}_i\right)}^2}{\sigma_i^2}} $$

Where *x*_*i*_ is the value of feature *i* and *y*_*i*_ is the mean of feature *i* across all samples within the group [25]. The SED per feature is then normalized using the estimated variance of feature *i*. SED can also be calculated for each pairwise comparison within the group. In this case, instead of using *y*_*i*_ as the mean of feature *i* it is another sample within the group. By examining the distance between the sample and the group center or other members of the group, it is possible to identify potential problematic samples. If the SED exceeds a threshold, then the sample is identified as a possible outlier. The distances between samples are presented in terms of box and whiskers plots. The examples of the Standardized Euclidean Distance tool graphic outputs are provided in the Additional file 2: Figures S10 and S11.

The SED relies solely on geometric distance and ignores the dependency structure between features. The Mahalanobis distance (MD) is a more general distance which can incorporate the correlation structure. MD relies on the estimate of the inverse of the variance-covariance matrix [40].

The Mahalanobis distance (MD) is a more general distance which can incorporate the correlation structure. MD relies on the estimate of the inverse of the variance-covariance matrix ∑^−1^ [29]. For sample vector *x* and *y* where each vector has *n* elements the Mahalanobis distance has the form:

$$ MD\left(x,y\right)=\sqrt{{\left(x-y\right)}^T{\sum}^{-1}\left(x-y\right)}. $$

When the dependency between metabolites is ignored the inverse variance-covariance matrix ∑^−1^ simplifies to diagonal matrix with diagonal values $1/{\sigma}_i^2$ for *i* = 1, 2, …, *n* and the MD simplifies to the SED. Since the inverse variance-covariance matrix used in MD is not defined when the number of features is bigger than the number or samples a penalized inverse variance-covariance matrix was used instead. The penalized version includes a common regularization [41] that is well described in the literature [42]. The details are provided in Additional file 3 for completeness. PMD provides output in the same format as SED. An example of the Penalized Mahalanobis Distance tool graphic outputs are provided in the Additional file 2: Figures S12 and S13.

### Data analysis tools

The data analysis tools include the following: *Single Group t-test, t-test, Group Comparison by Permutation, Analysis of Variance* (ANOVA), *Kruskal-Wallis, Hierarchical Cluster*, *LASSO/Elastic Net*, *Modulated Modularity Clustering* (MMC), *Multiple Testing Adjustment* (MTA), *Partial Least Squares Discriminant Analysis* (PLS-DA), *Principal Component Analysis* (PCA), *Linear Discriminant Analysis* (LDA), *Random Forest* (RF), and *Support Vector Machine* (SVM).

The *Single Group t-Test, t-test, Group Comparison by Permutation, Kruskal-Wallis, and Analysis of Variance* (ANOVA) tools compare the means of the data in different group(s) feature by feature [43, 44]. SECIMTools implements a fully fixed ANOVA framework that allows covariates in the model, an additional feature compared to many of the existing Galaxy ANOVA tools. All pairwise contrasts are calculated and for each contrast -values are produced. The model is based on the standard assumptions of normal and identically distributed random errors. There is an option to include an interaction effect between variables if more than one categorical variable is present. Output includes raw -values for each contrast, model diagnostics and volcano plots for each contrast (log base 10 -value against the difference between the group means) [45]. The examples of the *Analysis of Variance* tool graphic outputs are provided in the Additional file 2: Figures S14 and S15. *The Single group t-test* compares mean feature values against a fixed value (default,zero) and can be used to test differences between paired samples. The output includes raw -values, flags, and volcano plots. The *t-test* compares two groups with both paired and unpaired options. Paired samples are identified in the design file. Output includes raw -values, flags, and volcano plots. The examples of the *Single Group t-test* and *t-test* tools graphic outputs are provided in the Additional file 2: Figures S26 and S27. *Group Comparison by Permutation* calculates a t-statistic as in the t-test tool but determines the probability under the null of the t-statistic using permutation of the data. Output includes raw -values, flags, and volcano plots. *Kruskal-Wallis* is a non-parametric test [44] and takes the same input files as ANOVA, and provides -values, significance flags, and volcano plots as output files. The example of the graphic outputs for the *Kruskal-Wallis* tool are provided in the Additional file 2: Figure S28.

The *Multiple Testing Adjustment (MTA)* takes as input the raw -values. Three adjustment methods based on the false discovery rate (FDR) have been implemented; Bonferroni [46], Benjamini/Hochberg (BH) [47] and Benjamini/Yekutieli (BY) [48]. The tool produces a table containing columns with the -values for each adjustment method used.

*Hierarchical Clustering* [49, 50] is implemented using a centroid distance. The method relies on the assumption and properties of the multivariate normal distribution (MVN). This tool outputs a hierarchical clustering heatmap plot. The examples of the Hierarchical Clustering tool plot outputs are provided in the Additional file 2: Figures S16 and S17.

The *Modulated Modularity Clustering* (MMC) tool visualizes the latent structure in the data from weighted graphs [26, 51]. The method relies on the assumption and properties of the multivariate normal distribution (MVN). Pairwise correlations are calculated for all possible metabolite pairs. Then the correlations are sorted to identify groups of correlated metabolites. This tool is a wrapper for the python code developed by the algorithm developers [26] and made available via the GNU license. Output from the tool includes an estimate of the number of distinct correlated clusters and the metabolites in each cluster as well as unsorted, sorted, and sorted and smoothed dependency heatmaps. The example of the Modulated Modularity Clustering tool plot output is provided in the Additional file 2: Figure S18.

The *Principal Component Analysis (PCA)* calculates principal components (PCs) [49, 52]. The method relies on the assumption and properties of the multivariate normal distribution (MVN). All the PCs are orthogonal and are placed in the descending order based on the variability in the data that each PC explains. Multiple algorithms can be used to conduct PCA, SECIMTools utilizes the singular value decomposition (SVD) approach [53]. Visual summaries are provided in the form of 2D and 3D scatter plots using the first three principal components. The samples in the scatter plots are colored based on the group provided in the design file. The examples of the Principal Component Analysis tool plot outputs are provided in the Additional file 2: Figures S19 and S20.

The *Partial Least Squares Discriminant Analysis (PLS-DA)* is a tool based on partial least squares regression and binary response [54]. The method is applied to two groups. The tool produces 2D plots for comparison between the treatment groups and a file containing scores and weights of the model. Pairwise 2D plots are produced by default for the first two components only. Additional plots can be made using the plotting tools. Cross validation and double cross validation options are available to determine the best number of components for sample sizes larger than 100. The example of the Partial Least Squares Discriminant Analysis tool plot output is provided in the Additional file 2: Figure S21.

The *Linear Discriminant Analysis (LDA)* tool is a supervised method based on the underlying assumption of normality for each group under consideration and the same variance-covariance structure between the groups [49, 55]. The goal of the LDA is to find a linear partition (hyperplane) in multidimensional subspace that maximizes the separation between the groups under consideration. The dimension of the considered subspace has to be smaller than the number of groups. The method is well described in the literature [49, 42]. Cross validation and double cross validation options are available to determine the best number of components used for the subspace for sample sizes larger than 100. Visual summaries are provided pairwise for each two dimensions where the points for each treatment group are colored differently. The example of the Linear Discriminant Analysis tool graphic output is provided in the Additional file 2: Figure S22.

The *Random Forest (RF)* tool uses the random forest algorithm [22], to assign an importance score to every feature and rank order them. The importance score is a measure of how differentiating that feature is in a classification task, where the classes are the treatments group or any other feature that indicates the class labels. In the former case, the tool can be used to identify the most differentiating factors between treatment groups, where it provides variable importance plot (VIF) for the most important features. Unlike PCA, where the transformed features are rank-ordered by the level variance they contain, rank-ordering of the features in RF is directly measured by a “usefulness” score in an ensemble of decision trees. The ensemble is created by randomly choosing both the samples and features used to create and train a decision tree. This random ensemble approach has proven to be a useful regularizer, hedge against over fitting when sample sizes are adequate but is not a panacea [56]. The example of the Random Forest tool plot output is provided in the Additional file 2: Figure S23.

The *Support Vector Machine (SVM)* tool is a machine learning classifier for high dimensional data [23–25]. Using a set of labeled data (the label identifies which class the sample belongs to) as a training set, the SVM algorithm builds a model that can be used to predict the class label for the new and unclassified samples. The method performance depends on the sample size and the effect size [57]. Since high-dimensional data points are likely not separable by a linear hyperplane, SVM allows one to use non-linear kernel functions to separate the data points better in a non-linear space. To use the SVM tool, user must have both a training dataset with known categories in the design file and a target dataset. The tool then predicts the category for each sample in the target set. It also reports the accuracy of the trained model on the original training dataset. Cross validation and double cross validation options are available to determine the value of the regularization parameter for sample sizes larger than 100.

The *LASSO/Elastic Net* tool performs a selection of features that are different for each pairwise comparison between the groups in the grouping variable specified by the user. The selection is performed based on the logistic regression with Elastic Net shrinkage [21]. LASSO which stands for least absolute shrinkage and selection operator [20] is a special case of Elastic Net and is also included in the tool. The selection method is defined by shrinkage parameter α (defined within [0;1] range) specified by the user (default value α = 0.5). The value α = 1 corresponds to the least number of variables and the strictest selection criterion (LASSO), while α = 0 corresponds only to the estimated shrinkage without variable selection (ridge regression) [41]. The best subset of variables for a given α are selected. The examples of the LASSO/Elastic Net tool graphic outputs are provided in the Additional file 2: Figures S24 and S25. This tool is a wrapper for the R code developed by the inventors of the statistical approach and distributed under the GNU license [58].

**Fig. 3:**
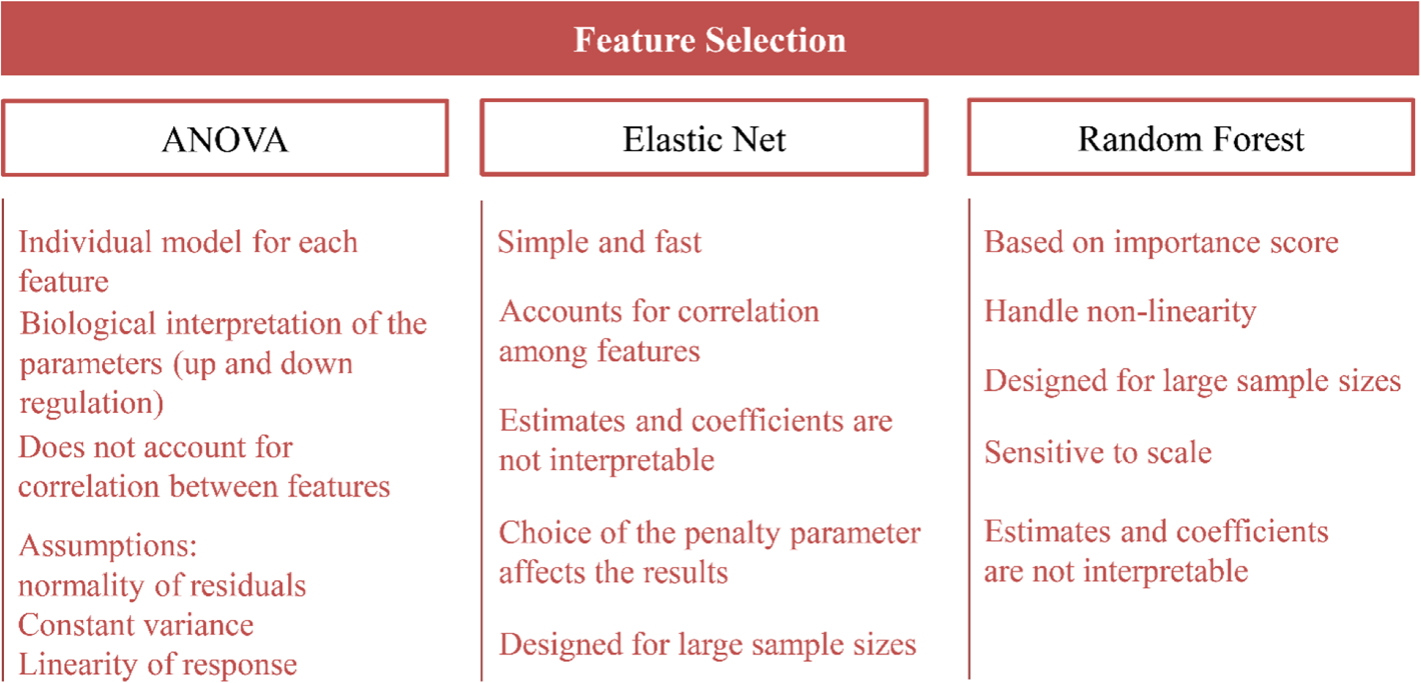
Summary of ANOVA, Random Forrest and LASSO/Elastic Net methods with their advantages and disadvantages

The summary comparison between ANOVA, Random Forrest and LASSO/Elastic Net methods is provided in Figs. 3.

### Utilities

Utilities are the auxiliary tools designed to facilitate users handling and processing of data. They are used to merge, filter, summarize and plot. The utilities included on the suite are *Compare Flags, Compound Identification Merge Flags, Modify Design File, Mass to Charge Ratio/Retention Time (m/z/RT) Matching*, *Remove Selected Features or Samples*, *Scatter Plot 2D*, *Scatter Plot 3D and Summary of the Flags*.

The *Compare Flags* tool compares two flags from a single flag files and produces a comparison table. When used with output from classification methods such as LDA, this tool can be used to produce the confusion matrix. Flags from multiple files can by compared after they are merged using the *Merge Flags* tool.

The *Compounds Identification* tool was designed to link a user’s library of compounds with the features identified in the analysis. The matching between the compound names and dataset feature ID-s is performed by comparing m/z and RT values within an error window (user specified). The users of this tool must have their own library of compound names and corresponding m/z and RT values in the wide format to be able to use the *Compounds Identification* tool.

The *Remove Selected Features or Samples*, *Merge Flags,* and *Summary of the Flags* tools were designed to work with the output files containing binary indicators for each feature. The *Merge flags* and *Summary of the flags* tools combine binary indicator files and produce summaries of indicators. The *Remove Selected Features or Samples* tool creates a new wide dataset where user identified column from the flag file is used to remove features. The *Modify Design File* tool allows the user to remove samples from the design file and to create a subset of the design file. The output is a new design file where specified group(s) of samples are removed.

The *Scatter Plot 2D* and *Scatter Plot 3D* tools were designed for plotting. The user has an option to select a coloring scheme using a grouping variable from the design file and a customizable color palate.

The *Mass to Charge Ratio/Retention Time (m/z/RT) Matching* can be used to match features from different parameter settings of peak calling programs. Each feature is characterized by mass to charge ratio and retention time (m/z and RT). Features are linked using mass to charge ratio and retention time for each feature, with a small interval window (user defined). Input files must contain at least three columns: mass to charge ratio (m/z), retention time (RT) and identifier (feature ID). The example of the Mass to Charge Ratio/Retention Time (m/z/RT) Matching tool graphic summaries outputs are provided in the Additional file 2: Figures S29, S30 and S31.

## Results

### Workflows and tool availability

**Fig. 4:**
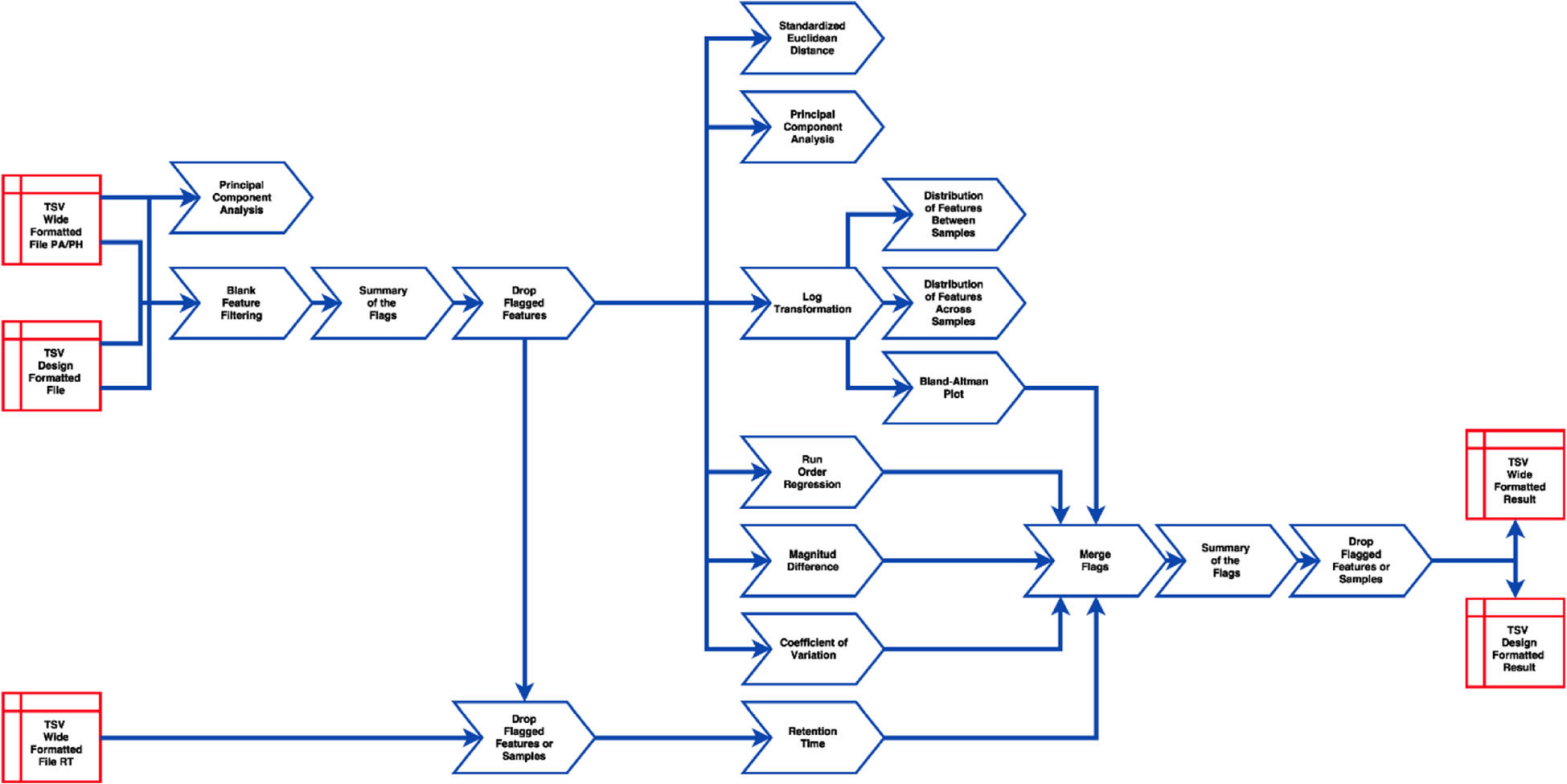
An example of data preprocessing and Quality Control for MS data. The workflow begins with the Blank Feature Filtering, and removal of the features below the level of detection. The Standardized Euclidian Distance, the Principal Component Analysis, the Run Order Regression, The Magnitude Difference, the Coefficient of Variation, and the Retention Time tools are used for the diagnostics at the next step. Some tools require log transformed data for the input, and the Log/G-Log Transformation tool is included into the workflow to address that. Multiple summary flags are produced by each tool. The tool’s flags are merged and summarized with the option to delete flagged features

**Fig. 5:**
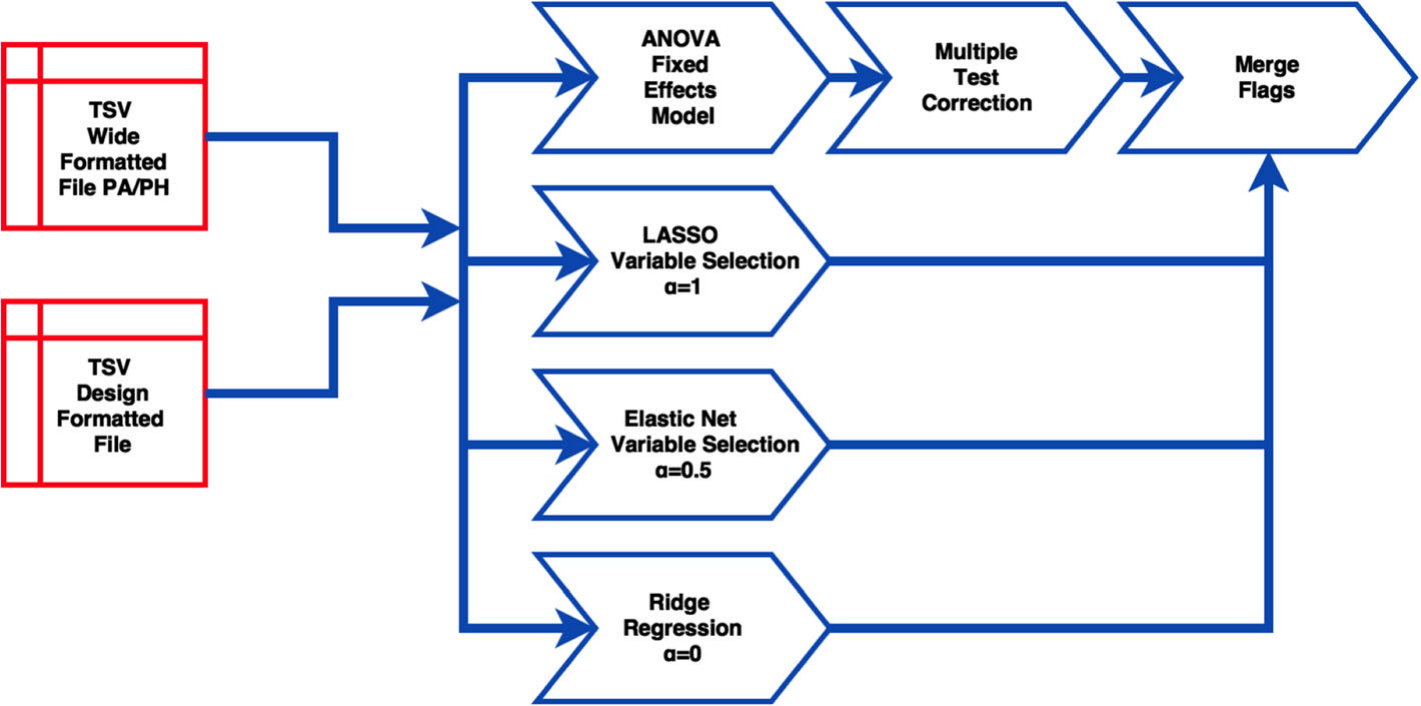
Workflow for ANOVA and Variable Selection. This workflow compares α = 0 Ridge Regression, α = 0.5 Elastic Net and α = 1 for LASSO to results from an ANOVA

The Galaxy platform provides a framework for the easy construction and implementation of workflows. The user has complete flexibility to choose the tools to be included into the workflow and the order of their execution. All the intermediate steps of the workflow remain in the history, allowing the user to track every step and potential discrepancies in the data. Some examples of the workflows are presented in Figs. 4 and 5.

## Installation

Installation of SECIMTools and their dependencies into Galaxy instances can be done in multiple ways depending on the local environment and the dependency resolution mechanism used in an instance. In general, any galaxy tool consists of the interface definition written in xml and the underlying tools and tool dependencies needed to run a Biocomputing analysis. SECIMTools can be installed either from the Galaxy Toolshed [41] or manually with the tool dependencies handled either automatically via one of the tool dependency resolvers or via a manual installation. Most SECIMTools consist of a tool definition xml file that describes the tool interface in the galaxy, a wrapper script written in python that drives the analysis, and underlying python module (Python 2.7 compatible) or third party executable dependencies that encompass the low-level functionality required for the analysis.

To simplify the installation we packaged all tools as a python package available from https://pypi.python.org/pypi/secimtools. The python package can be used with a modern tool dependency resolution approach of using environmental modules, docker, or the ‘conda’ package manager [59] via the bioconda project [60]. For instance, a Conda package manager has been available in Galaxy since the 16.01 release and is recommended for all instances running 16.07 release or newer code. We will provide a ‘secimtools’ conda package as a reference tool dependency (pending). For an older, developmental, or customized instance of Galaxy, which may either require rapid tool updates, preclude the use of a Conda package manager, or use a different resolver, a clone of the SECIMTools master branch from the SECIMTools Git repository [27] and a resolver configuration [61]; or a manual installation of specified dependencies into the Galaxy virtual environment; or via the environment modules mechanism are required. A list of all the specific libraries and functions used by SECIMTools is available by examining the dependencies for each tool.

## Conclusions

Untargeted metabolomics is a relatively new field. Analysis development has been primarily in self-contained web or Java-based standalone toolkits [11, 62]. The Galaxy platform has a modular structure and has been successfully used to bring bioinformatics to individual scientists with minimal computational background. Galaxy was designed to run via web browser providing a user-friendly, cross-platform setting that can be configured on global servers available in large universities [63] or locally oriented for small research groups and individual researchers. SECIMTools suite takes advantage of the Galaxy interface and its code is available to the community under the terms of MIT license on GitHub [27].

Source code for the Galaxy is open and supported by the developer community, which means it is constantly improved and enhanced. Modern research is characterized by its interdisciplinary nature and cooperation among scientists. Data analysis may be shared across groups and performed by people with different backgrounds at different locations. Reproducibility has recently become a focus in the scientific community and is a crucial component of the success of the scientific method [64–66]. Galaxy addresses reproducibility requirements by allowing tracking histories and allowing scientists to create reproducible workflows. Histories and workflows are easily shared amongst users, facilitating collaborative research.

SECIMTools compliments other metabolomics toolkits developed for Galaxy [13, 14]. The sophisticated QC and statistical techniques are currently not widely available to scientists working with metabolomics data without in depth knowledge of programming. Many of the modern statistical approaches in SECIMTools are not available in the stand-alone metabolomics analysis platforms, and have not previously been incorporated in the Galaxy platform. Having a potential wider applicability to other omics data and other novel tools that enhance metabolomics analysis (RT, BFF) is a distinct advantage of SECIMTools. The choice of Galaxy will allow for future integration of metabolomics analysis with other omics analysis and brings metabolomics forward.

## Additional files

Additional file 1: User Guide. (PDF 3648 kb)

Additional file 2: Example input and output. (DOCX 3818 kb)

Additional file 3: Mahalanobis Distance calculation. (PDF 102 kb)
