# Supplementary material for: SECIMTools: a suite of metabolomics data analysis tools
Source: BMC Bioinformatics. 2018 Apr 20;19:151. doi: 10.1186/s12859-018-2134-1 (PMC5910624; doi:10.1186/s12859-018-2134-1)
Supplement: Supplementary file 1 — User Guide. (PDF 3648 kb) [file 12859_2018_2134_MOESM1_ESM.pdf]

Table of Contents

Data Input 3

Data Output 7

Analysis of Variance (ANOVA) Fixed Effects Model 8

Bland-Altman (BA) Plot 12

Blank Feature Filtering (BFF) Flags 16

Compare Flags 18

Coefficient of Variation (CV) Flags 19

Compound Identification 21

Data Normalization and Re-Scaling 23

Distribution of Features across Samples 25

Distribution of Features within Samples 27

Hierarchical Cluster Heatmap 29

Imputation (Mean, Median, K-Nearest Neighbours (KNN), Stochastic) 30

Kruskal-Wallis Non-Parametric Test 33

LASSO/Elastic Net Variable Selection 36

Linear Discriminant Analysis (LDA) 39

Log and G-Log Transformation 42

Mass to Charge Ratio/Retention Time (m/z/RT) Matching 44

Magnitude Difference Flags 48

Modify Design File 50

Merge Flags 51

Modulated Modularity Clustering (MMC) 52

Multiple Testing Adjustment (MTA) 54

Partial Least Squares Discriminant Analysis (PLS-DA) 56

Penalized Mahalanobis Distance (PMD) 59

Principal Component Analysis (PCA) 63

Random Forests (RF) 65

Remove Selected Features or Samples 67

Retention Time (RT) Flags 69

Run Order Regression (ROR) 71

Scatter Plot 2D 73

Scatter Plot 3D 75
Standardized Euclidean Distance (SED) 77
Summary of the Flags 80
Support Vector Machine (SVM) Classifier 81
Threshold Based Flags 85
T-Test (Single Group) 86
T-Test (Unpaired or Paired) 88
Group Comparison by Permutation 91

Data Input

This section describes the structure and the format of the input datasets for SECIMTools on Galaxy. Only data files in text format can be used in SECIMTools.

Tabular File Structure

All the data files used for calculations in Galaxy **must** be text files and use the tabular delimiter. If your data are not saved in a tabular delimited file they will not appear in the dropdown menus for the SECIMTools. Tab delimited files often use the .tab or .tsv extensions.

A mechanism for the file conversion is included in Galaxy. The following sequence of steps can be used to convert a text file with an arbitrary delimiter to a text file with a tabular format:

1. Select the *Text Manipulation* menu on the left side of the screen.
2. Select the *Convert* tool which will convert delimiters to TAB.
3. Select the delimiter to convert (e.g. comma, whitespace, and colon) and the dataset you wish to convert.
4. Press the "Execute" button and new tabular delimited dataset will be created.

Wide Format Dataset

A wide formatted dataset should contain feature measurements for each sample with samples in columns and features in rows. Feature information may also be included in wide format datasets (e.g. average mass to charge ratio and retention time). The dataset must contain a column with a **unique** identifier for each row (Unique Feature ID). For example the column "Compound" in the figure above. In the wide dataset all the columns containing data to be analyzed should to be numeric. Missing values, can be represented by empty cells or cells with a common value denoting a missing observation. Missing values can be characters (e.g. NA). Please see the section on naming conventions below for more information about naming column headers.

Compound	sample1	sample2	sample3	...
compound1	10	20	10	...
compound2	5	22	30	...
compound3	30	27	2	...
compound4	32	17	8	...
...	...	...	...	...

Long Format Dataset

A long formatted dataset contains one column containing the numeric data and another column (or columns) uniquely identifying the numeric data. One of the columns should contain sample identifiers identical to the sample identifiers in the Design Dataset. This column must be called 'sampleID'. The other column contains numeric values (e.g. scores generated from a PCA) for each sample. In cases where there are multiple variables for each sampleID (e.g. peak intensities),

additional columns such as the unique Feature identifier may be required to fully describe the content of the value column.

sampleID	PC1	PC2	PC3
RC001_1	179203288.514	-8870447.24785	-6403864.11847
RC002_1	-59955188.197	20610844.0082	37100502.3101
RC003_1	-119248100.31	-11740396.7448	-30696638.1696
RC004_1	-733053175.232	153301347.981	33722673.2567

Design Dataset

The design dataset is used to relate the sample values in the wide format dataset with sample characteristics. Examples of sample characteristics are: treatment group, batch number, sample weight, order in which the sample was processed in the instrument (run order). The sample identifiers used as column headers in the wide dataset **have to** be saved in the design file with the column name sampleID. This is necessary for the tools to link the two files during analysis. Spelling of the sampleID column name is case sensitive. Values in the design dataset can be categorical (such as grouping variable containing treatment name or batch name) or numeric (such as sample weight or run order). Please see the section on naming conventions below for more information about how to name column headers.

```
+----------+--------+--------+
| sampleID | group1 | group2 |
+==========+========+========+
| sample1  | g1     | t1     |
| sample2  | g1     | t1     |
| sample3  | g1     | t1     |
| sample4  | g2     | t1     |
| sample5  | g2     | t1     |
| sample6  | g2     | t1     |
| ...      | ...    | ...    |
+----------+--------+--------+
```

Relationship between the Wide Format Dataset and the Design Dataset

In most of the tools the sampleID column in the design file is used to link sample information to the metabolomics data in the wide format dataset. The values for the column name in the wide format and in the sampleID column in the design file must match exactly and the matching algorithm is case sensitive. If there are values in the sampleID column that do not match a column name in the wide dataset a warning will be generated. Warnings are shown in the Tool Standard Output and the Tool Standard Error links in the information section for each tool output. To access the output click on the circular "eye". If there are columns with the data in the wide dataset that are absent from the sampleID column in the design file those columns will not be included in analysis.

NOTE: Keep in mind that all column names and values inside the column names are case sensitive i.e. Sample01 and sample01 are not the same and that a space is treated as a character. When a value in the sampleID column is saved with an extra space before (or after) the name this will not match the column header in the wide dataset.

Annotation File

The annotation files use the wide format files that link the featureID-s to the corresponding compound names and additional feature information. The annotation files are expected to have at least two columns: one containing the featureID-s and the other contains the corresponding compound names.

Flag File

Flag files are auxiliary datasets that are produced by many of the SECIMTools and are also used as an input by some SECIMTools. The flag files are named in this way to indicate that they usually contain columns of binary values (1/0) even though the other columns are possible. These files are used to identify (flag) features or samples. The flag files can either be in the wide format or in the design format.

For the flag files produced by the SECIMTools, the wide format flag files have the unique feature identifier column (featureID) and additional flag column(s) used to classify the features. The columns that start with the name flag_ contain binary values (0 or 1).

The design format flag files have the sampleID column and additional flag column(s) used to classify the samples. The columns with the flags contain binary values (0 or 1). The column indicating the sampleID must have values that match the values in the design file, but the name of the column in the flag file is arbitrary.

Users can import their own flag files. The tools that accept flag files are flexible and allow any categorical variable. Users only need to be mindful that the sampleID or featureID column values in the flag files have to match the corresponding values in the data files exactly.

NOTE: Flag files may contain multiple columns. In addition to binary flags, the flag files may contain other columns with information generated by a specific tool or information imported from external processes.

Naming conventions

There are naming conventions used by SECIMTools. These follow the conventions and restrictions of the Linux operating system and the tabular text file format used by Galaxy. The majority of the programming languages recognize variable names that follow these rules. Most of these rules are straightforward, however when dealing with chemical data there are some particular challenges. The design file values should not contain a comma (,) in the column name. Compound names often contain special characters that can be problematic in some contexts. Since SECIMTools use several published algorithms by other authors and incorporate many functions

from other software packages, users are advised to separate these columns into an annotation file during analysis and join them at the end of the analysis for results interpretation.

Column names and values in the sampleID and featureID columns **should** use roman letters from the English alphabet, **should not:** start with a number; be longer than 32 characters; or have special characters. The only permitted special character is the underline symbol (_).

Examples of special characters that are not recommended are $, €, £, #, %, space, dot, hyphen, single and double quotes, punctuation. Examples of letters that are not recommended are α, φ, β (Greek), ц, ю, ж (Cyrillic), ą, ś, ł. The column names that contain a space (e.g. sample 1) are particularly problematic as the space may not be treated consistently across the Galaxy platform. Examples of the **recommended** column names are: sample01, measure_1, s_01, s_num1_batch01, ozone_rep1.

To address possible naming violations that may cause problems the SECIMTools suite has a build-in string cleaning interface executed prior to running any tool. The interface replaces special characters with '_' symbol and prepends the '_' symbol to the column names and featureIDs that start with numbers. By default tools like MZmine output unique identifiers that are numeric for each row. The SECIMTools interface prevents this common convention from being problematic in operating within the Galaxy environment.

Metabolomics Workbench data compatibility

SECIMTools provides compatibility with the Study Design Data table and the Results Data table from the Metabolomics Workbench repository. Rename the first column of the Study Design Data Table to "sampleID" and verify that the names in the Study Design Data table and in the Results Data table follow the naming conventions described earlier. Some minor name changes of the Metabolomics Workbench datasets may be required to address the Galaxy required naming conventions before using them in SECIMTools.

Data Output

Data Output in Galaxy comes in two formats: PDF files that contain figures and TSV files that contain tab separated data.

The PDF file stands for the Portable Document Format file. The PDF files can be viewed in any appropriate document viewer (such as Adobe Acrobat Reader or DigiSigner) or browser (such as Mozilla Firefox or Google Chrome). The PDF files can also be edited (e.g. Adobe Acrobat Professional, Adobe Illustrator or Inkscape).

The TSV files are text files that have tab separated values. These files can be opened in any editor suitable to work with text (e.g. Microsoft Excel, Libre Office Calc, OpenOffice Calc).

Viewing Output

To view the output files click on the view button ("eye" symbol) in the green box in the history on the right side of the screen. If the tool outputs multiple files each file will have a separate view button.

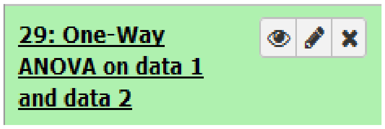

Saving Output

To download and save the output to the local machine click on the title of the output in the history. This will expand the box and give more information about the tool output. After expanding the history green box, click on the download button ("diskette" symbol) on the bottom of the box to save the file to a local machine.

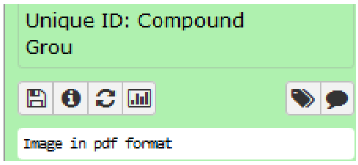

NOTE: TSV files produced by Galaxy have ".tabular" extension. Depending on the platform (e.g. Mac vs PC), operating system (e.g. MacOS, Windows, Linux) or browser used (e.g. Firefox, Chrome, Safari) warning when attempting to download and to open ".tabular" extension files may occur. The .tabular extension can be changed manually to ".tsv" or ".tab".

Analysis of Variance (ANOVA) Fixed Effects Model

The tool fits an analysis of variance (ANOVA) fixed effects models with multiple grouping variables, their interactions and covariates. The analysis is performed independently for each row. The user can choose whether to include interactions between grouping variables in the model or to use a pure additive model Click on the *Analysis of Variance (ANOVA) Fixed Effects Model* tool in the SECIMTools menu on the left side of the screen.

The ANOVA fixed effects model is fit performed using the “ols” function from the statsmodels package in Python.

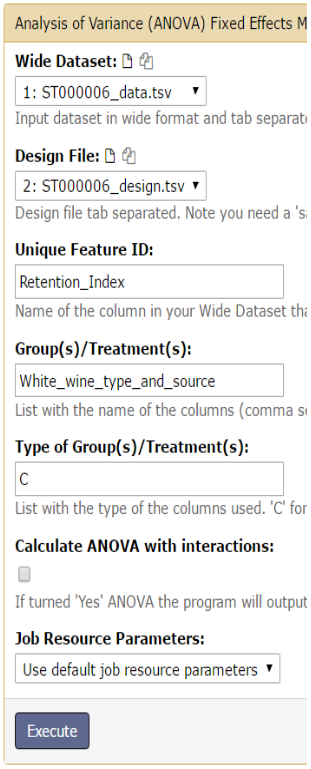

1. Select the **Wide Dataset** from the drop down menu.
2. Select the **Design File** from the drop-down menu.
3. In the **Unique Feature ID** field type the name of the unique Feature identifier.
4. In the **Group(s)/Treatment(s)** text box, type the names of the columns in the **Design File** that identify the variables used for the model. Categorical and continuous variables are supported. Separate the column names with a comma and without a space. For example “treatment_group,gender,age”. Column names are case sensitive.
5. In **Type of Group(s)/Treatment(s)** text box, input the variable types. These are specified as C for categorical and N for continuous and must be in the same order as the variable names. For example for “treatment_group,gender,age” input “C,C,N”
6. Clicking on the checkbox **Calculate ANOVA with interactions** will calculate all possible pairwise interactions between the **Group(s)/Treatment(s)** variables. Numerical variables are not included in the interactions.
7. Click **Execute**

Output

This tool outputs two files:

- TSV file for the results table containing the fixed effects ANOVA results for each variable, the corresponding contrast and analysis of the **means**.
    - Diff of {group_i-group_j}: difference in the mean for samples in group j compared to samples in group j
    - GrandMean: Mean across all samples
    - SampleVariance:Variance across all samples
    - Mean {group_i}: mean for samples in group i
    - StdError_for_Diff_{ group_i-group_j}
    - t-Value_for_Diff { group_i-group_j}
    - Prob>|t|_for_Diff{ group_i-group_j}
    - -log10(p-val)_{ group_i-group_j}
    - f-Value for the fixed effect of group
    - p-Value of f-Value
    - ErrorSS: Sum of squares for error in the overall model
    - ModelSS: Sum of squares for the model in the overall model
    - TotalSS: ErrorSS+ModelSS
    - MSE:mean square error
    - NDF: numerator degrees of freedom
    - DDF:denominator degrees of freedom
    - R2
- TSV file with flags equal to 1 if :
    - Flag_significant_0.05_on_{group_i-group_j} equal to 1 if Prob>|t|_for_Diff{ group_i-group_j} is less than 0.05
    - Flag_significant_0.01_on_{ group_i-group_j } equal to 1 if Prob>|t|_for_Diff{ group_i-group_j} is less than 0.01
    - Flag_significant_0.1_on_{ group_i-group_j } equal to 1 if Prob>|t|_for_Diff{ group_i-group_j} is less than 0.1

- PDF file for the Q-Q (quantile-quantile) plots.

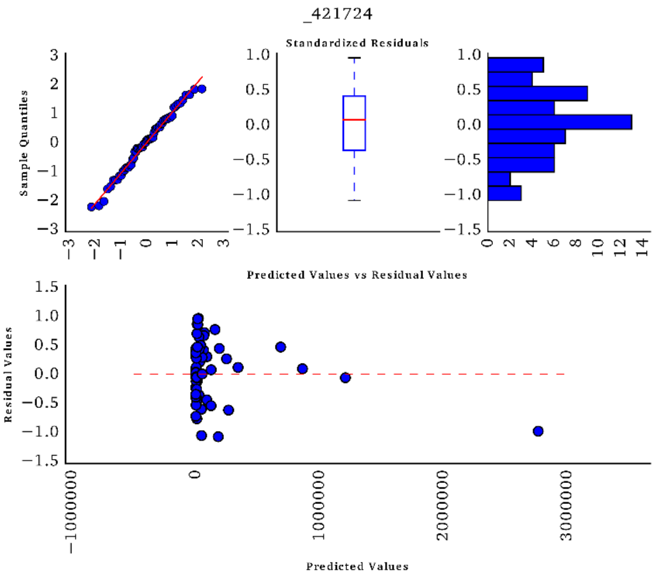

The Quantile-Quantile Plot (QQ Plot) is generated by the ANOVA Fixed Effects Models (top left). The QQ plot displays the expected quantiles of a normal distribution on x-axis versus the observed quantiles on y-axis. A boxplot (top center) of the residuals and corresponding histogram of the residuals (top right). In the bottom figure the x-axis contains the observed values, their corresponding residuals are on the y-axis.

- PDF file for the volcano plots.

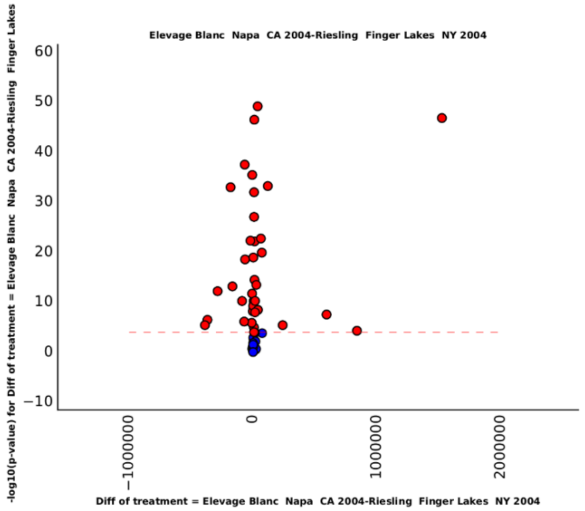

A volcano plot is generated by the ANOVA Fixed Effects tool. On the plot, the differences between the group means are displayed on the x-axis. The corresponding -log base 10 p -values from the test of the null hypothesis are displayed on the y-axis. Each dot represents a feature. One volcano plot is generated for each pairwise comparison. The red dashed line in the volcano plot(s)

corresponds to a p-value = 0.01 (2 on the negative log base 10 scale). Volcano plots were developed by Wei et al.:

Jin, Wei, Rebecca M. Riley, Russell D. Wolfinger, Kevin P. White, Gisele Passador-Gurgel, and Greg Gibson. "The contributions of sex, genotype and age to transcriptional variance in Drosophila melanogaster." Nature genetics 29, no. 4 (2001): 389-395.

Bland-Altman (BA) Plot

The Bland-Altman plot (BA-Plot) is used to look at the concordance of data between pairs of samples, particularly between replicates. The script generates BA-plots for all pairwise combinations of samples. If both a grouping variable name and a specific group name are provided, then the BA-Plots are generated only for pairwise combinations within the specified group.

In addition to generating BA-plots, a linear regression fit is produced between the values that correspond to the pair of samples to identify (flag) any unusual outlying feature values. The flags produced by the regression fit are used to generate distribution plots and text files for (i) each sample (column) and for (ii) each feature (row).

Bland-Altman plots are described in the reference below:

Bland JM, Altman D. 1986. Statistical methods for assessing agreement between two methods of clinical measurement. The Lancet. 327:307-310.

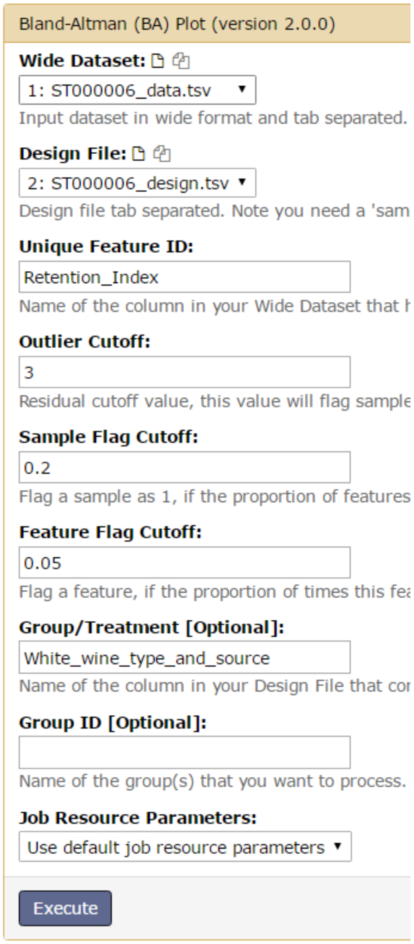

1. Select the **Wide Dataset** from the drop-down menu
2. Select the **Design File** from the drop-down menu.
3. In the **Unique Feature ID** text box, type the name of the unique feature identifier.
4. In the **Outlier Cutoff** text box, type a cutoff value for residuals. Any residuals with values ≥ the cutoff value will be flagged. (Default value is 3).
5. In the **Sample Flag Cutoff** text box, type a cutoff value for flagging samples (Default value is 0.2) If the proportion of features flagged for a particular sample is greater than the cutoff specified, then the sample will be flagged.
6. In the **Feature Flag Cutoff** text box, type a cutoff value for flagging features (Default is 0.05) If the proportion of samples for which that feature is flagged is greater than the cutoff specified then the sample will be flagged.
7. In the **Group/Treatment [Optional]** text box, type the name of the column in the **Design File** that identifies your groups. This will run the analysis by groups, if you want to run this analysis pairwise for all samples this box should be left empty.
8. In the **Group ID [Optional]** text box, type the name of a specific group. This will perform the analysis exclusively on this group.
9. Click **Execute**

Output

This tool outputs four files: depending on the input settings.

- TSV file for the feature flags with the column.
    - **Unique Feature ID**
      flag_feature_BA_outlier: 0/1 flag for each feature where "1" indicates that this feature was identified as an outlier. The **Feature Flag cutoff** is by default 0.05, meaning that 5% of the values in that feature need to be flagged as outliers for that feature to be flagged as well. The comparison measures used are Pearson residuals, DFFITS, and Cooks D.
- TSV file for the samples flags with the column.
    - **sampleID**
    - flag_sample_BA_outlier: 0/1 flag for each feature where "1" indicates that this sample was identified as an outlier. The **Sample Flag cutoff is** by default 0.2, meaning that 20% of the values in that sample need to be flagged as outliers for the sample to be flagged as well. The comparison measures used are Pearson residuals, DFFITS, and Cooks D.

- PDF file for BA-Plots.

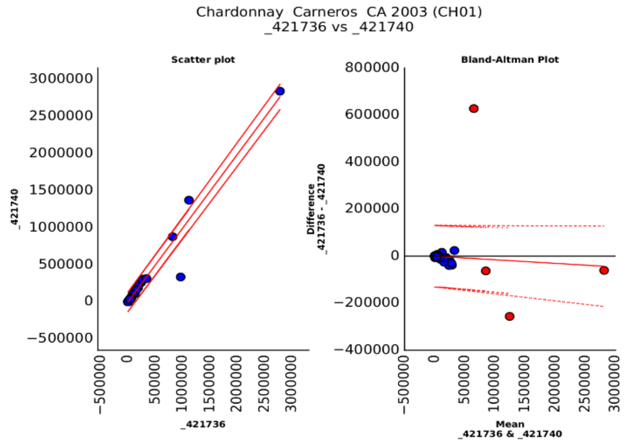

- The Scatter plot (left) displays the values of the first sample (_421736) on the x-axis and the values of the second sample (_421740) on the y-axis. Samples representing replicates are expected to have similar feature values. The Bland-Altman plot (right) displays the mean of each feature for the two samples on the x-axis and the corresponding difference between features on the y-axis. A linear regression fit and the corresponding confidence bands are displayed on each graph in red. The slope of the line is expected to be zero. The Red dots represent the outliers that have been identified (flagged) using at least one of these methods: Cook's Distance, Pearson Residuals and DFFITSPDF file of bar graphs for samples and features with the most outliers.

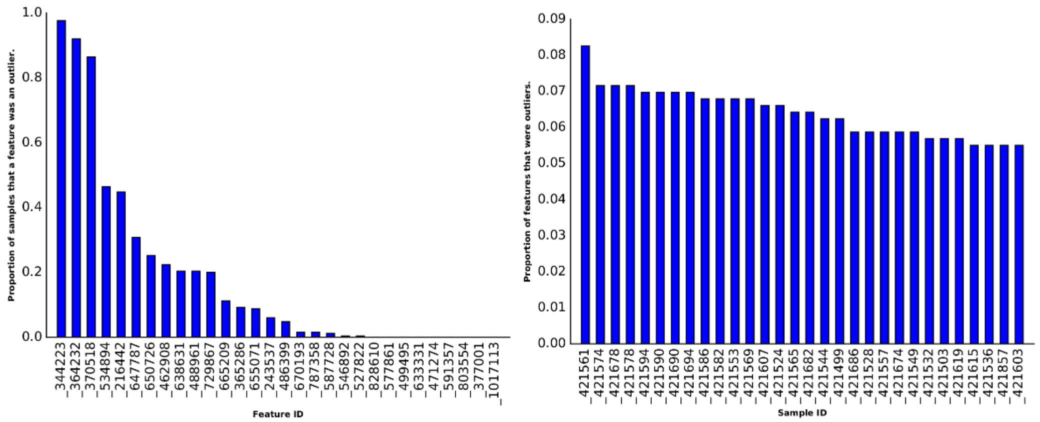

Histogram of 30 most flagged features (left) and samples (right) produced by the BA Plot Tool.

Blank Feature Filtering (BFF) Flags

NOTE: The tool is relevant for Mass Spectroscopy (MS) data analysis and allows removing "noise" from the data using values in the blank samples as a reference.

This tool uses features in negative (blank) control samples to calculate a limit of detection and flag features in non-blank samples that are below this limit. The BFF Threshold for each feature is equal to (3*Standard Deviation of the blank group) + (the average of the blank group) and is computed across blank samples only. If, for a given feature, the computed BFF Threshold is less than or equal to 0, the user specified BFF Threshold overrides the computed BFF Threshold (default value for user specified BFF Threshold is 5000). The user-specified BFF threshold is particularly important when the blank group contains a lot of zero values or is on log-transformed scale. A feature is flagged as below the detection limit for a given group if the ratio (group mean – BFF Threshold) / BFF Threshold is less than the Criterion Value (default 100) for the average within that group.

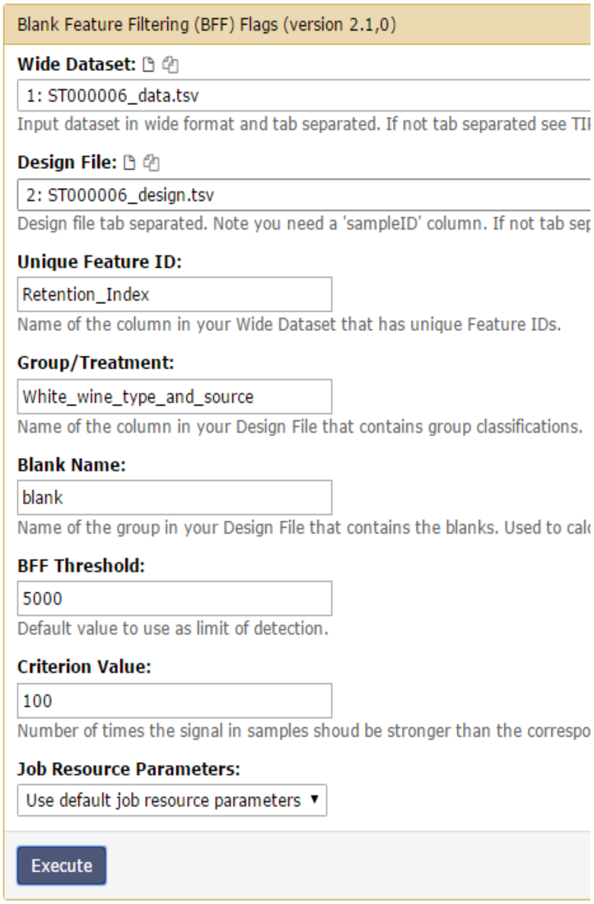

1. Select the **Wide Dataset** from the drop-down menu.
2. Select the **Design File** from the drop-down menu.
3. In the **Unique Feature ID** text box, type the name of the unique feature identifier.
4. In the **Group/Treatment** text box, type the name of the column in your **Design File** that contains the group classifications.
5. In the **Blank Name** text box, type the name from the **Group/Treatment** column that indicates which samples are blanks.
6. In the **BFF threshold** text box, type a value to use as a threshold should the calculated BFF threshold be less than or equal to 0. The default value is 5000.
7. In the **Criterion Value** text box, input the value to use as criteria for the ratio of (group mean – BFF Threshold) / BFF Threshold. The default value is 100.
8. Click **Execute.**

Output

This tool outputs two files:

- TSV file with values compared to the Criterion Value. It contains the following columns:
    - **Unique Feature ID**
    - A column for each **Group/Treatment**: Contains the value of (group mean – BFF Threshold) / BFF Thresholds for each feature. The BFF Threshold is calculated as (3*Standard Deviation of the **Blank Name** group) + (the average of the **Blank Name** group) and is computed across **Blank Name** samples only.

- TSV file containing flags for each feature. Flag values of one (1) correspond to features which failed to satisfy the BFF Threshold Criterion Value and are considered below the detection limit for the given group. Columns are:
    - **Unique Feature ID**
    - flag_bff_{**Group/Treatment**}_off: 0/1 indicator flag for each feature. A feature is flagged as below the detection limit for the given group if the ratio (group mean – BFF Threshold) / BFF Thresholds is less than the **Criterion Value.** The value "1" is assigned to those features that fall below the **Criterion Value**.

Compare Flags

This tool compares two flag columns in a flag file and creates a 'cross tabulation' results file. Flags from multiple flag files can be combined by first running the 'Merge_Flags' tool.

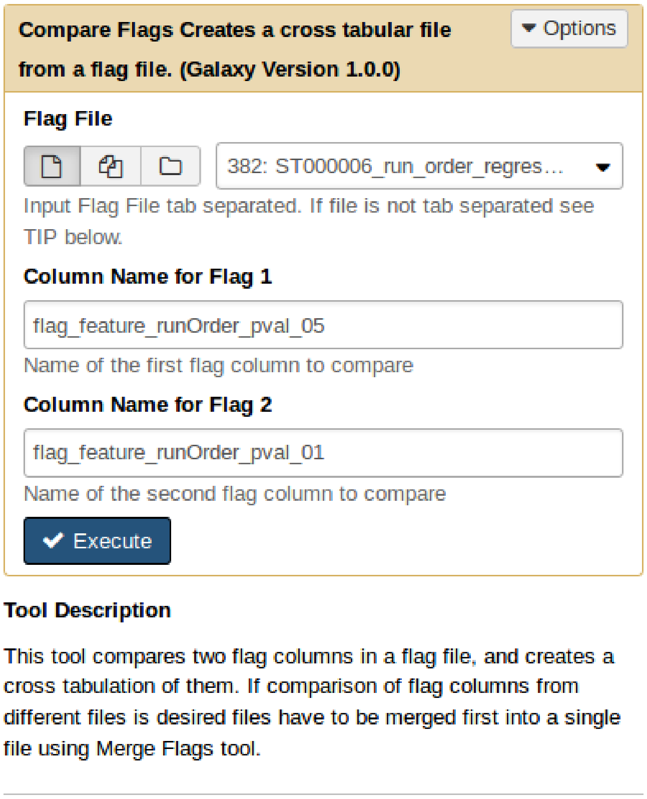

1. Select the **Flag File** from the drop-down menu.
2. In the **Column Name for Flag 1** text box, enter the name of the first flag column to compare.
3. In the **Column Name for Flag 2** text box, enter the name of the second flag column to compare.
4. Click **Execute**

Output

This tool outputs a single file:

- TSV file containing the frequencies of the compared flags in the appropriate cells.

Coefficient of Variation (CV) Flags

This tool calculates the coefficient of variation (standard deviation as a percentage of the mean) and is often used to look at the consistency of features across samples. The user can define what percent of features with the highest CV to flag. If no percentage is selected, then the top 10% of features with the highest CV are flagged (default value). The CV value corresponding to the percentage is indicated in the output plots.

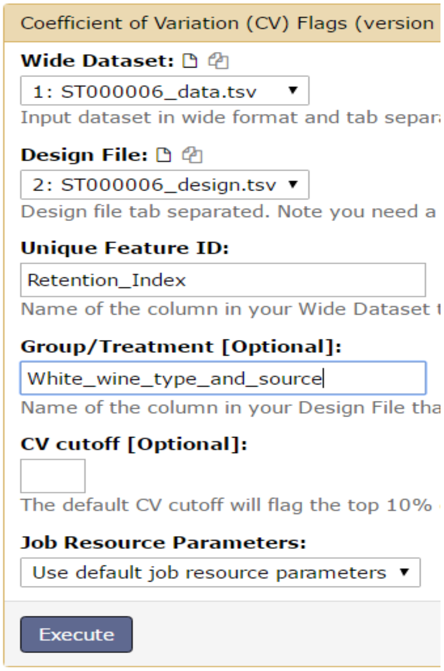

1. Select the **Wide Dataset** from the drop-down menu.
2. Select the **Design File** from the drop-down menu.
3. In the **Unique Feature ID** text box, type the name of the unique feature identifier.
4. In the **Group/Treatment [Optional]** text box, type the name of the column in the **Design File** that identifies the groups.
5. In the **CV cutoff [Optional]** text box, enter a CV cutoff to flag the top X% of features with the largest CV's. Enter the value as a decimal (e.g.: 10% = 0.10). If no value is entered, the top 10% (Default = 0.1) of features with the highest CV's are flagged.
6. Click **Execute**

Output

This tool outputs two files:

- TSV file containing the CV Flags for each feature for each group (if group variable is specified). A flag value of one (1) corresponds to features with large CV values as specified by the CV cutoff. :
    - **Unique Feature ID**
    - flag_feature_big_CV_{**Group/Treatment [Optional]**}: 0/1 indicator flag for each feature. The value "1" is assigned to those features with CV values that exceed the CV cutoff criteria.

- PDF file containing histograms with overlaid density plots of the coefficients of variation for each group (optional, if the group variable is provided) and a summary density plot containing the densities for each group without the histograms.

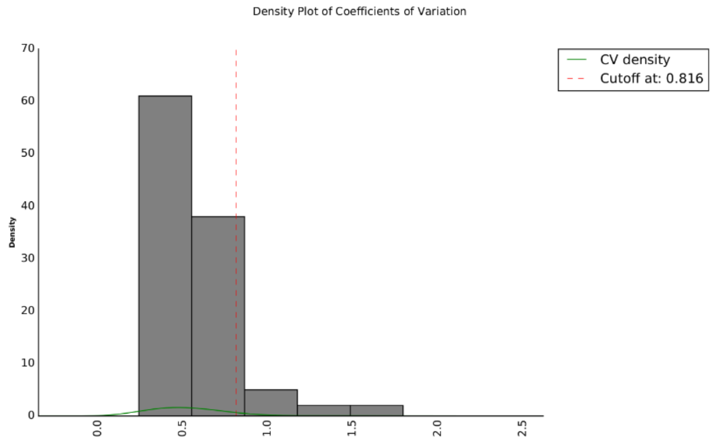

Histogram and density plot (in green) of the coefficients of variation for each feature across all samples. The dotted red line indicates the CV value corresponding to the top 10% of coefficient of variation values and the actual value that corresponds to the top 10% is also displayed in the legend.

Compound Identification

NOTE: This tool is primarily intended for identification of compounds in a target file given a mass spectroscopy library file.

Each metabolite (feature) is characterized by mass to charge (m/z) ratio and retention time (RT). This tool matches two files: (1) a mass spectroscopy library file and (2) a target annotation file. The library file (in tsv format) contains a list of compounds and their associated m/z ratios and RTs. The target annotation file contains the m/z ratios and RTs for the experimental samples. The unique identifier in the target annotation file is matched to compound name(s) from the library file based on the m/z ratio and RT. The match is performed using a window around the m/z ratio and a window around the RT.

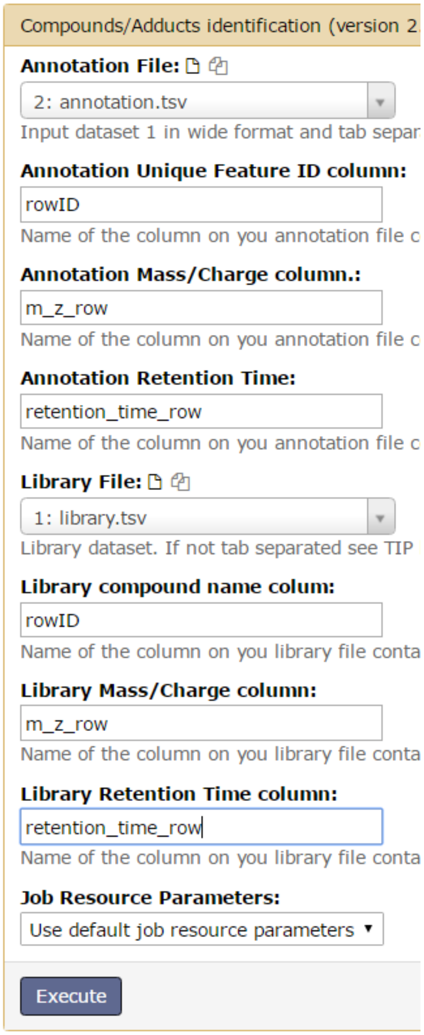

1. Select the **Annotation File** from the dropdown menu. This file contains m/z ratios and RTs for which compound identification is wanted.
2. In the **Annotation Unique Feature ID column** text box, specify the Unique ID in the **Annotation File**.
3. In the **Annotation Mass/Charge column** text box, specify the column in your **Annotation File** that contains the m/z ratio for the features.
4. In the **Annotation Retention Time** text box, specify the column in your **Annotation File** that contains the Retention Time for the features.
5. Select the **Library File** from the dropdown menu. This file contains a list of compounds and their associated m/z ratios and RTs.
6. In the **Library Unique Feature ID column** text box, specify the Unique ID in the **Library File**.
7. In the **Library Mass/Charge column** text box, specify the column in your **Library File** that contains the m/z ratio for the features.
8. In the **Library Retention Time** text box, specify the column in your **Library File** that contains the Retention Time for the features.
9. Click **Execute.**

Output

This tool outputs one file:

- TSV file. This file will contain the original target annotation input file plus an additional column containing the name of any compounds matching the m/z ratio and RT.
    - **Target Annotation Unique Feature ID column**
    - **Target Annotation Mass/Charge column**
    - **Target Annotation RT column**
    - **Library Unique Feature ID column:** If a compound is matched, this column will contain the name of the compound. If no compound is matched this field will be empty.
    - **{Additional columns}:** Note, Any additional columns present in the **Annotation File.**

NOTE: For features (rows) with no match in the library file, the compound column will have no value (be blank).

Data Normalization and Re-Scaling

The first three normalization methods (Mean, Sum and Median) perform re-scaling of the data by sample. Each individual sample (column) in the wide dataset is re-scaled by dividing of all the feature values within that column by the mean, median or sum of those feature values. Each sample (column) is re-scaled independently from the other samples (columns).

The last six normalization methods (Centering, Pareto, Autoscaling, Range, Level, and Variable Stability (VAST)) perform scaling of the data by features. Each feature (row) is re-scaled independently from other features. Each individual feature (row) in the wide dataset is centered by subtraction of the mean of that feature and is re-scaled by dividing all the feature values within that row by the scaling factor. The scaling factor is computed from the feature values in the current row and depends on the selected method. Centering does not have a scaling factor and does not perform division, Autoscaling uses the standard deviation, Pareto scaling uses the square root of the standard deviation, Range uses the difference between the max and min values, and Level uses the mean. VAST scaling is performed in two steps. The first step is Autoscaling, followed by division of the resulting feature values in each row by the coefficient of variation for that feature.

More information on the scaling methods are available via the references:

Keun, Hector C., Timothy MD Ebbels, Henrik Antti, Mary E. Bollard, Olaf Beckonert, Elaine Holmes, John C. Lindon, and Jeremy K. Nicholson. "Improved analysis of multivariate data by variable stability scaling: application to NMR-based metabolic profiling." Analytica chimica acta 490, no. 1 (2003): 265-276.

van den Berg, Robert A., Huub CJ Hoefsloot, Johan A. Westerhuis, Age K. Smilde, and Mariët J. van der Werf. "Centering, scaling, and transformations: improving the biological information content of metabolomics data." BMC genomics 7, no. 1 (2006): 142

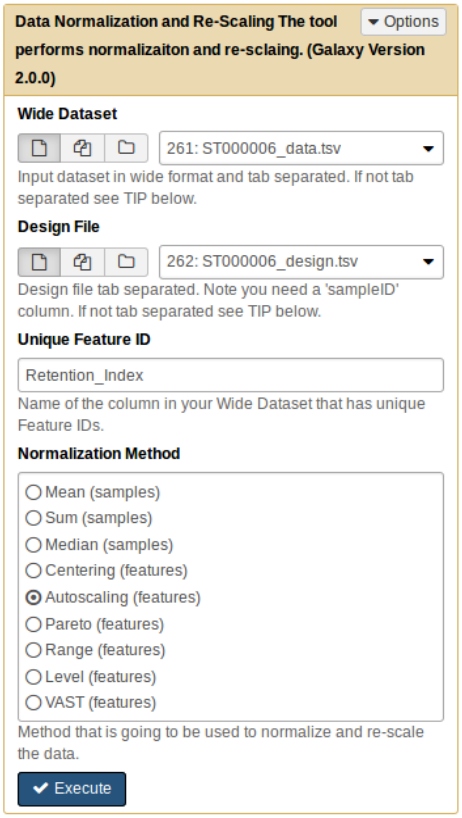

1. Select the **Wide Dataset** from the drop-down menu.

2. Select the **Design File** from the drop-down menu.

3. In the **Unique Feature ID** text box, type the name of the **Unique feature identifier**.

4. Select the **Normalization Method** using the radio button. Each method indicates whether it is applied to samples or features.

5. Click **Execute.**

Output

This tool outputs one file:

- TSV file containing the same column names as in the original **Wide Dataset** where the values in each cell correspond to the values after normalization/re-scaling.

NOTE: Rows with missing values and samples that disagree with the design file will be omitted from the analysis.

Distribution of Features across Samples

The tool summarizes the distribution of 50 randomly selected features (rows) across all samples. Boxplots with outliers and mean value are provided for each selected feature across all samples.

If group or treatment information is provided, boxplots are generated for samples within each group and for all samples. If a group or treatment variable is not provided, boxplots are provided for all samples in the dataset.

As an additional summary, all features are summarized in a single density plot. If group or treatment information is provided, then one density plot is provided for all features and all samples within each group. A single plot is also provided for all samples and features, ignoring the group information. If no group or treatment information is provided, a single density plot is provided for all features and all samples in the dataset.

NOTE: While it recommended to use the tool with log transformed data, the tool remains functional for non-transformed data.

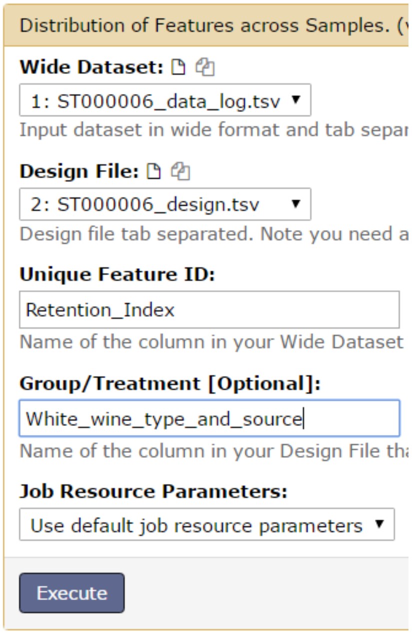

1. Select the **Wide Dataset** from the drop-down menu.
2. Select the **Design File** from the drop-down menu.
3. In the **Unique Feature ID** text box, type the name of the **Unique feature identifier**.
4. In the **Group/Treatment [Optional]** text box, type the name of the column in the **Design File** that identifies your groups or treatments.
5. Click **Execute.**

Output

The tool outputs one file:

- PDF file with boxplot(s) and density plot(s): if the **Group/Treatment [Optional]** variable is provided plots will be generated for every group as well as for all samples. Otherwise, a single plot will be generated for all samples.

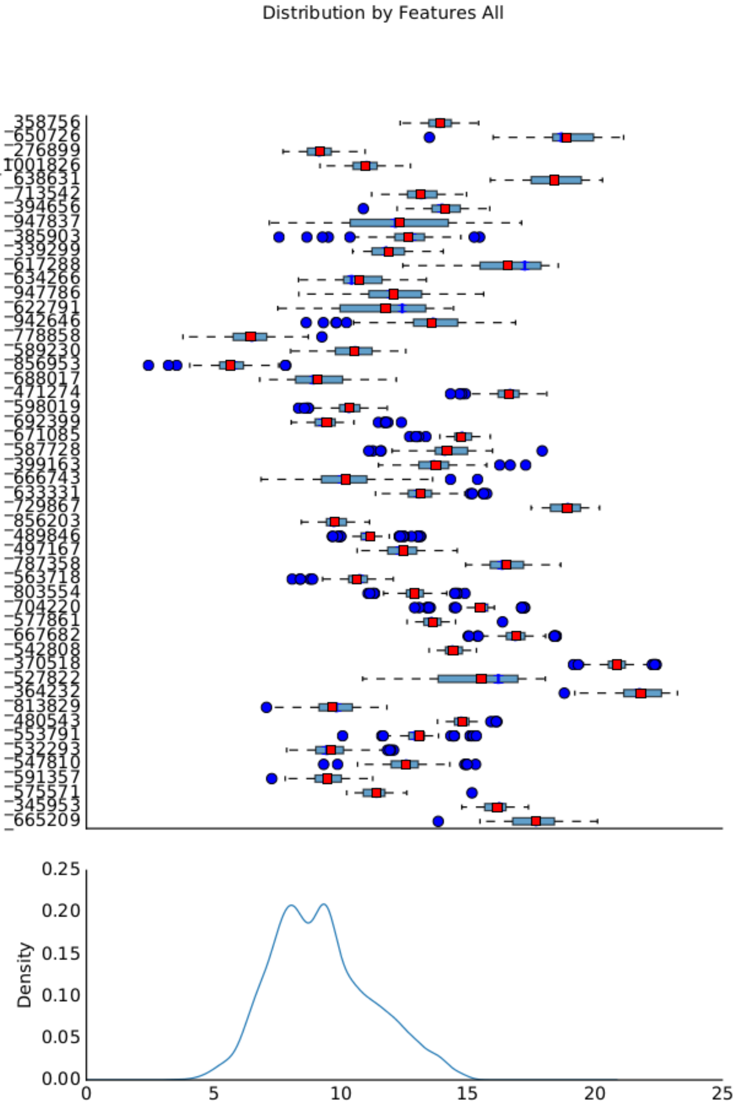

Boxplots of the values for 50 randomly selected features across the treatment groups (top). Density plot for all features across all samples is presented in one graph (bottom).

Distribution of Features within Samples

The tool plots the distribution of features within each sample. All samples are colored by group and are plotted on the same graph for comparison purposes. The distributions of the features within each sample are presented as estimated densities and box-and-whiskers plots with potential outliers. If the sample run order variable column name form the design file is specified in the input, box plots will be displayed according to the run order.

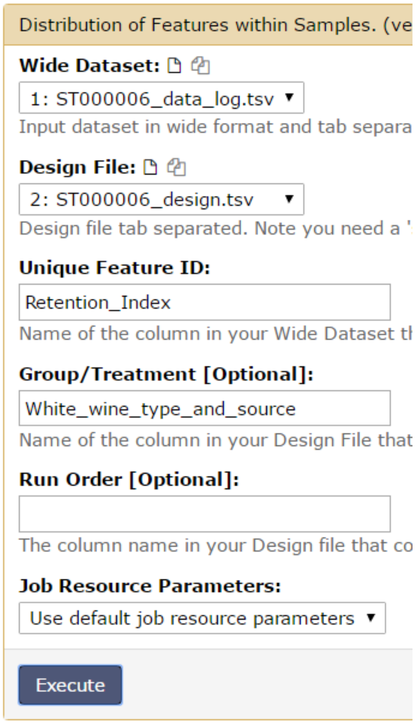

1. Select the **Wide Dataset** from the drop-down menu.
2. Select the **Design File** from the drop-down menu.
3. In the **Unique Feature ID** text box, type the name of the **Unique feature identifier**.
4. In the **Group/Treatment [Optional]** text box, type the name of the column in the **Design File** that identifies your groups.
5. In the **Run Order [Optional]** text box, type the name of the column in your design file that identifies the run order.
6. Click **Execute.**

Output

This tool outputs one file:

- PDF file containing two graphs:
  - Density plot.

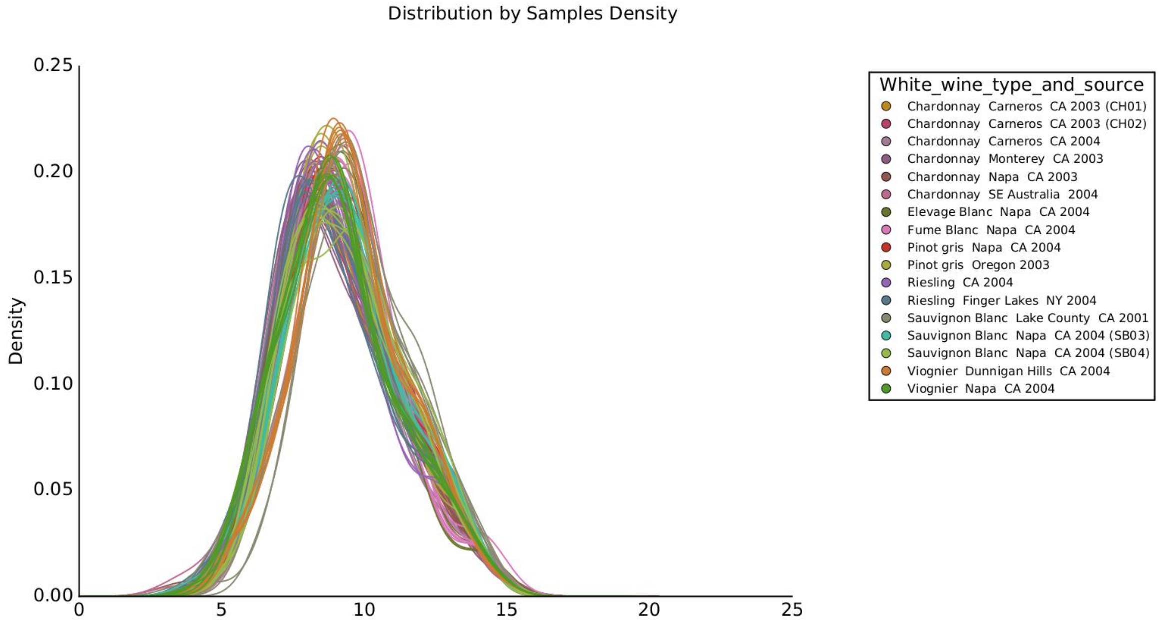

The density plots illustrate the distribution of features within the given sample. Each line represents a sample and its colored based on the group it belongs to.

  - *Box and whiskers* plot.

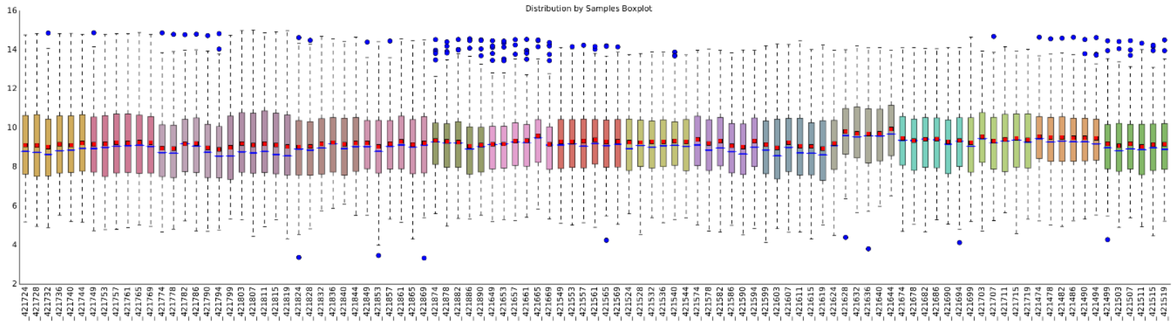

Boxplots of the distribution of features within each sample. Each boxplot is color coded based on the group it belongs to. Run order variable (if provided) defines the order of those boxplots.

Hierarchical Cluster Heatmap

This tool generates a hierarchical cluster heatmap from a wide format dataset. This tool works best on the log-transformed data without missing values. An option to add a hierarchical clustering dendrogram to the heatmap figure is included along with an option to remove plot labels.

The Hierarchical Cluster Heatmpaps are produced using the "heatmap" and "clustermap" functions from the seaborn package in Python.

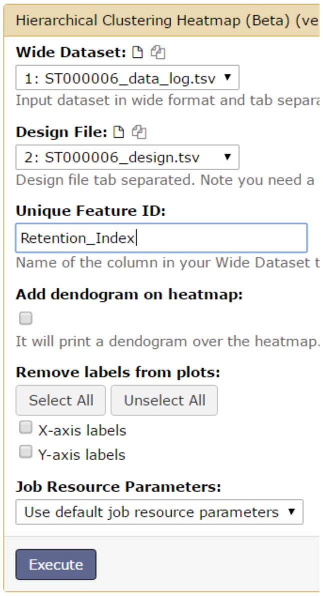

1. Select the **Wide Dataset** from the drop-down menu.
2. Select the **Design File** from the drop-down menu.
3. In the **Unique Feature ID** text box, type the name of the **Unique feature identifier**.
4. Under **Add dendogram on heatmap** click on the checkbox if you want to add a dendogram to the heatmap.
5. Under **Remove labels from plots** select whether to remove labels from any axis (x or y).
6. Click **Execute**.

Output

The tool outputs one file:

- A PDF file with a hierarchical cluster heatmap of the data

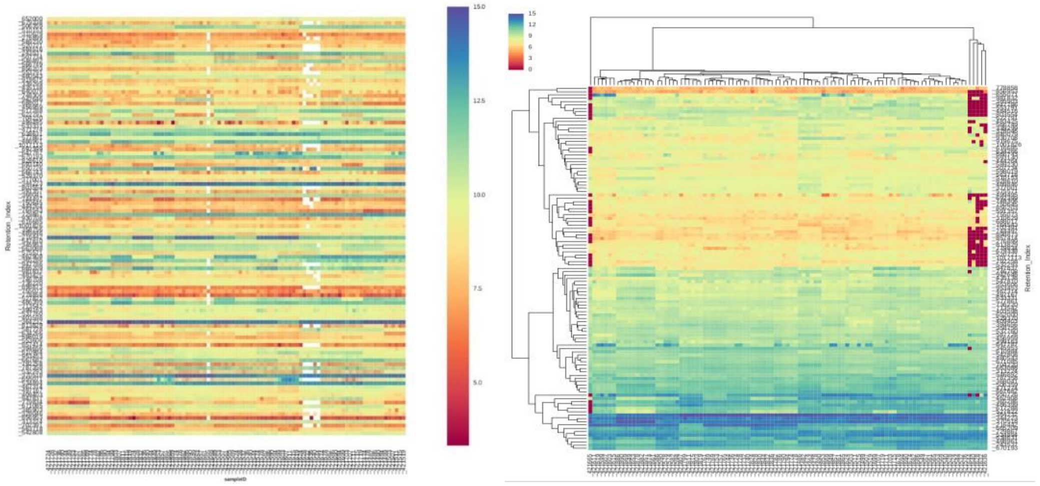

Heatmap representing the current dataset (left), different settings allow the usage of dendrograms (right).

Imputation (Mean, Median, K-Nearest Neighbours (KNN), Stochastic)

The tool performs an imputation procedure for missing data based on three conceptually different methods: (1) naive imputation (mean, median), (2) K-nearest neighbor imputation (KNN) and (3) stochastic imputation (based on normal and Poisson distributions). The user specifies which method to use.

Imputations are performed separately for each sample group since treatment groups are expected to be different. If only a single sample (column) is available for a given group, nothing is imputed and the sample is kept intact. An option to select which values should be treated as missing is included. The default value is an empty cell in the dataset with the option to treat zeroes, negative values and user-defined value(s) as missing and subsequently impute missing values.

(1) Naive imputation computes the mean (or median) of the features within the samples for a given group and uses that value to impute values for that feature among the missing samples. Feature values for all missing samples in the group get the same value equal to the mean (median) of that feature from the available samples, provided the allowed missing threshold is met.

(2) K-Nearest Neighbors (KNN) imputation is based on the procedure where the K nearest neighbor samples (default value K = 5) for the given sample within each group are considered. The neighboring samples are used to generate the missing feature value for the current samples. If less than the specified K neighbors are available for the current sample in the current group, then the maximum available number of neighbors is used. If the proportion of missing values for each row (feature) is greater than the specified Row Percent Cutoff value (default 0.5), then the column (sample) mean is imputed instead of feature values from the KNN algorithm. The maximum proportion of missing values for each column (sample) can be specified (Column Percent Cutoff default = 0.8) which determines whether a sample should be imputed or not. If the proportion of missing values for each sample is greater than the specified value, then the missing values are not imputed and the imputation process is interrupted. The algorithm is deterministic and always imputes the same missing values for the same settings.

The KNN imputation is performed using the "impute.knn" function from the impute package in R.

More details on the algorithm are available via the reference:

Olga Troyanskaya, Michael Cantor, Gavin Sherlock, Pat Brown, Trevor Hastie, Robert Tibshirani, David Botstein and Russ B. Altman, Missing value estimation methods for DNA microarrays BIOINFORMATICS Vol. 17 no. 6. 2001 Pages 520-525.

(3) Stochastic imputation is based on the assumption that each feature within a given group follows some underlying distribution. As a result, all missing values are generated from that underlying distribution. The parameter(s) of the underlying distribution is (are) estimated from the observed feature values within that group. Two distribution options are available: normal (recommended for logged and negative data) and Poisson (recommended for nonnegative counts). The normal

distribution parameters for each feature are estimated by the mean and standard deviation of that feature values among the observed samples in the given group. If all observed values for a feature are the same then the standard deviation is assumed to be 1/3 of the absolute value of the mean of those values. The Poisson distribution parameter is estimated by the mean of the observed values for a given feature and is expected to be positive for the imputation procedure to work correctly.

The stochastic imputation is performed using the “Impute”, “MCMC”, “Poisson”, and “Normal” functions from the PyMC package in Python.

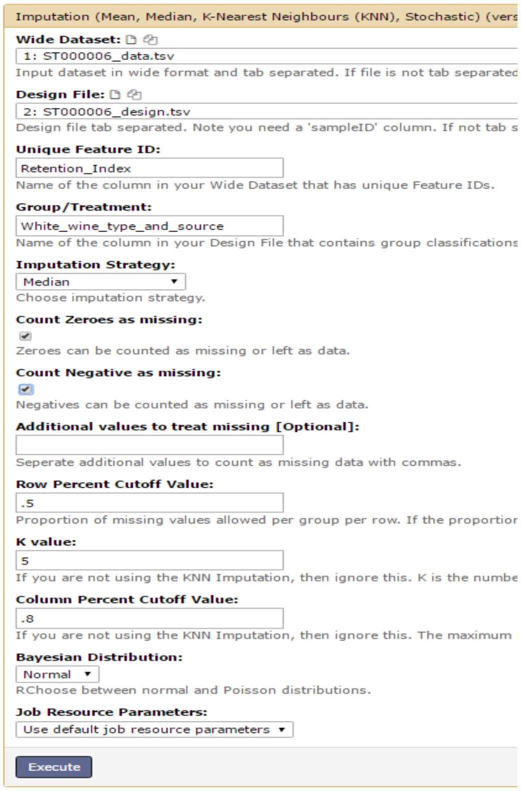

1. Select the **Wide Dataset** from the drop-down menu.
2. Select the **Design File** from the drop-down menu.
3. In the **Unique Feature ID** text box, type the name of the **Unique feature identifier**.
4. In the **Group/Treatment** text box, type the name of the column in the **Design File** that identifies your groups.
5. In the **Additional values to treat missing [Optional]** text box, type additional characters or values that are required to be treated as missing, they must be “,” separated.
6. Select the **Imputation Strategy** from the drop-down menu.
7. Click on the checkbox under **Count Zeroes as missing** if you want to treat “0” as a missing value.
8. Click on the checkbox under **Count Negative as missing** if you want to treat all negative values as missing.
9. In the **Row Percent Cutoff Value** text box, type the proportion of missing values allowed for each feature within a group (KNN **Imputation Strategy** only). The default value is 0.5. If the proportion of missing values for each feature is greater than the specified value the sample mean is imputed instead of values from the KNN algorithm.
10. In the **K value** text box, type the number of neighbors (K value.) (KNN **Imputation Strategy** only). K value is the number of neighbors to search. If less than 5 neighbors are available, all are used. The default is 5.
11. In the **Column Percent Cutoff Value** text box, type the proportion of missing values allowed for samples (KNN **Imputation Strategy** only). The default value is: 0.8. If the proportion of missing values is greater than the specified values the imputation stops and the tool returns the error.
12. Select the **Bayesian Distribution** from the drop-down menu (Stochastic **Imputation Strategy** only). The available options are Poisson and Normal.
13. Click **Execute.**

Output

This tool output one file:

- TSV file: containing the same column names as the original **Wide Dataset** where the values in each cell correspond to either the original values or to values obtained during the imputation procedure.

Kruskal-Wallis Non-Parametric Test

The tool performs a Kruskal-Wallis non-parametric test, an analog of the one-way ANOVA *F*-test that does not rely on the normality assumption of the distribution of features. Unlike *t*-tests or an ANOVA *F*-test, a Kruskal-Wallis test is based on ranks where ranks are compared between groups. The test is performed (1) for all samples from all groups together and (2) for all the samples belonging to each possible pair of groups.

The Kruskal-Wallis tests are performed using the "kruskalwallis" function from the SciPy package in Python.

The reader is referred to the literature for more details on the Kruskal-Wallis test and the computation/approximation of the corresponding p-values:

Kruskal, William H., and W. Allen Wallis. "Use of ranks in one-criterion variance analysis." Journal of the American statistical Association 47, no. 260 (1952): 583-621.

Meyer, J. Patrick, and Michael A. Seaman. "A comparison of the exact Kruskal-Wallis distribution to asymptotic approximations for all sample sizes up to 105." The Journal of Experimental Education 81, no. 2 (2013): 139-156.

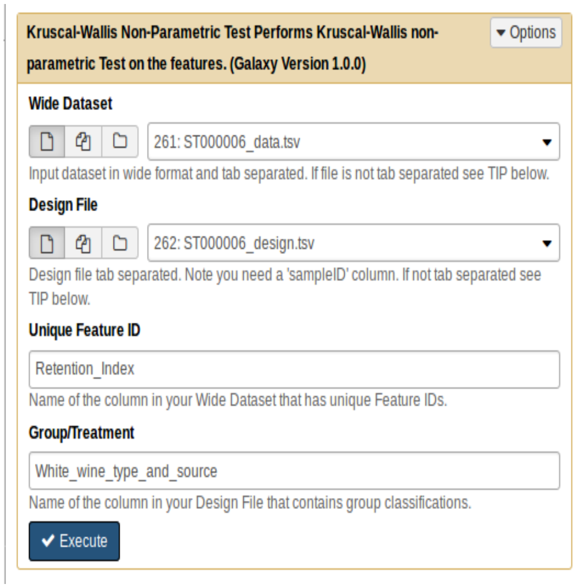

1. Select the **Wide Dataset** from the drop down menu.
2. Select the **Design File** from the drop-down menu.
3. In the **Unique Feature ID** field, type the name of the unique Feature identifier.
4. In the **Group/Treatment [Optional]** text box, type the name of the column in the **Design File** that identifies your groups.
5. Click **Execute.**

Output

A TSV file with summary statistics containing:

- GrandMean: Mean across all samples
- SampleVariance:Variance across all samples
- Mean {group_i}: mean for samples in group i
- Diff of {group_i_ group_j }: difference in the mean for samples in group i compared to group j.
- H-Value_for_Diff { group_i_ group_j }
- Prob>|H|_for_Diff{ group_i_ group_j }
- -log10(p-val)_{ group_i_ group_j }

A TSV file containing indicator flags. A flag = 1 if the difference in ranks between the compared groups is statistically significant.

- Flag_significant_0p05_on_all: equal to 1 if the difference in ranks between all groups is statistically significant using α=0.05
- Flag_significant_0p01_on_ all equal to 1 if the difference in ranks between all groups is statistically significant using α=0.01
- Flag_significant_0p1_on_ all equal to 1 if the difference in ranks between all groups statistically significant using α=0.1
- Flag_significant_0p05_on_{ group_i_ group_j }: equal to 1 if the difference in ranks between the group i and μ is statistically significant using α=0.05
- Flag_significant_0p01_on_{ group_i_ group_j } equal to 1 if the difference in ranks between the group i and μ is statistically significant using α=0.01
- Flag_significant_0p1_on_{ group_i_ group_j } equal to 1 if the difference in ranks between the group i and μ is statistically significant using α=0.1

A PDF file with the volcano plot(s) for the difference(s).

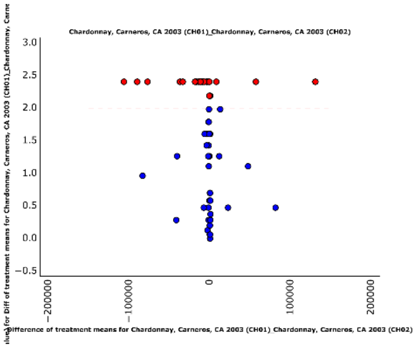

The volcano plot is produced based on the values of the differences between the means of group i and group j for each feature (x-axis) and the corresponding negative log (base 10) p-values (y-

axis). Each dot on the plot represents a feature. An individual volcano plot is generated for each pair of groups. The red dashed line in the volcano plot(s) corresponds to a p-value = 0.01 (2 on the negative log base 10 scale). The example of volcano plots in the literature can be found below:

Jin, Wei, Rebecca M. Riley, Russell D. Wolfinger, Kevin P. White, Gisele Passador-Gurgel, and Greg Gibson. "The contributions of sex, genotype and age to transcriptional variance in Drosophila melanogaster." Nature genetics 29, no. 4 (2001): 389-395.

LASSO/Elastic Net Variable Selection

The tool selects (identifies) features that are different between pairs of treatment groups. The selection is performed based on the logistic regression with Elastic Net shrinkage (with LASSO being a special case). The selection method is defined by shrinkage parameter α. Variable selection can be performed for any value of α in the range (0:1] where α = 1 corresponds to the fewest number of variables and the most strict selection criterion (LASSO) and α = 0 corresponds to shrinkage without variable selection (Ridge Regression). The default value is α = 0.5.

The LASSO/Elastic Net variable selection is performed using the “glmnet” and “cv.glmnet” functions from the glmnet package in R written by Friedman et.all. 2010. Details about the Elastic Net and LASSO methods can be found in the references below:

Zou, H., and Hastie, T. (2005). Regularization and variable selection via the elastic net. Journal of the Royal Statistical Society: Series B (Statistical Methodology), 67(2), 301-320.

Tibshirani, Robert. "Regression shrinkage and selection via the lasso." *Journal of the Royal Statistical Society. Series B (Methodological)* (1996): 267-288.

Friedman, Jerome, Trevor Hastie, and Rob Tibshirani. "Regularization paths for generalized linear models via coordinate descent." *Journal of statistical software* 33, no. 1 (2010): 1.

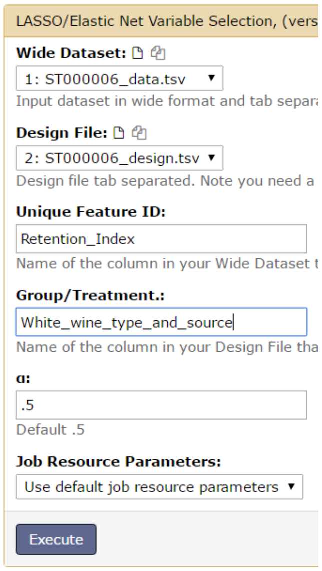

1. Select the **Wide Dataset** from the drop-down menu.
2. Select the **Design File** from the drop-down menu.
3. In the **Unique Feature ID** text box, type the name of the **Unique feature identifier**.
4. In the **Group/Treatment** text box, type the name of the column in the **Design File** that identifies your groups.
5. In the **α** text box, type the value for the **α** parameter. This parameter specifies the penalty for the LASSO/Elastic Net procedure. Default = 0.5
6. Click **Execute.**

Output

The tool outputs three files:

- A TSV file containing the values of the coefficients (including zeroes) for each feature generated by the algorithm for each pair of comparisons (in columns). These coefficients are produced from the transformed data (as part of the LASSO/EN method) and should be interpreted with caution.
  - {group_i_vs_group_j}: Coefficients for samples in group i vs samples in group j.

- A TSV file containing the corresponding flags for each feature.
  - {group_i_vs_group_j}_selection_flag_on: 0/1 flag where the value "1" corresponds to features selected by the method.

- A PDF file containing graphs for each pairwise comparison between the groups.

  - The first graph displays the behavior of the coefficients based on the value of penalty parameter λ..

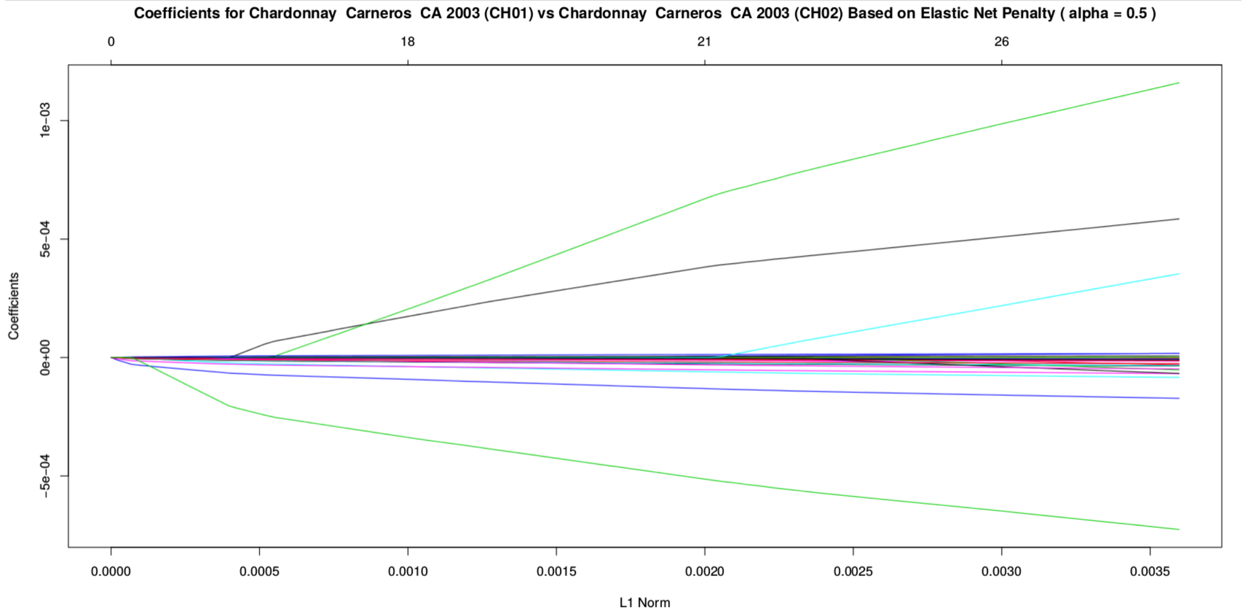

The plot shows the behavior of the LASSO/Elastic Net coefficients for the specified penalty split parameter α= 0.5. The value of α= 1 corresponds to the LASSO penalty split parameter. The value of the penalty parameter λ is displayed on the x-axis. The value λ = 0 (left part of the graph) forces all regression coefficients to be zero. The optimal value for the penalty λ is determined by a cross-validation procedure. For easier visualization the line for each coefficient (feature) has its own color.

- The second graph provides an estimate of the cross-validation error based on the log value of penalty λ.

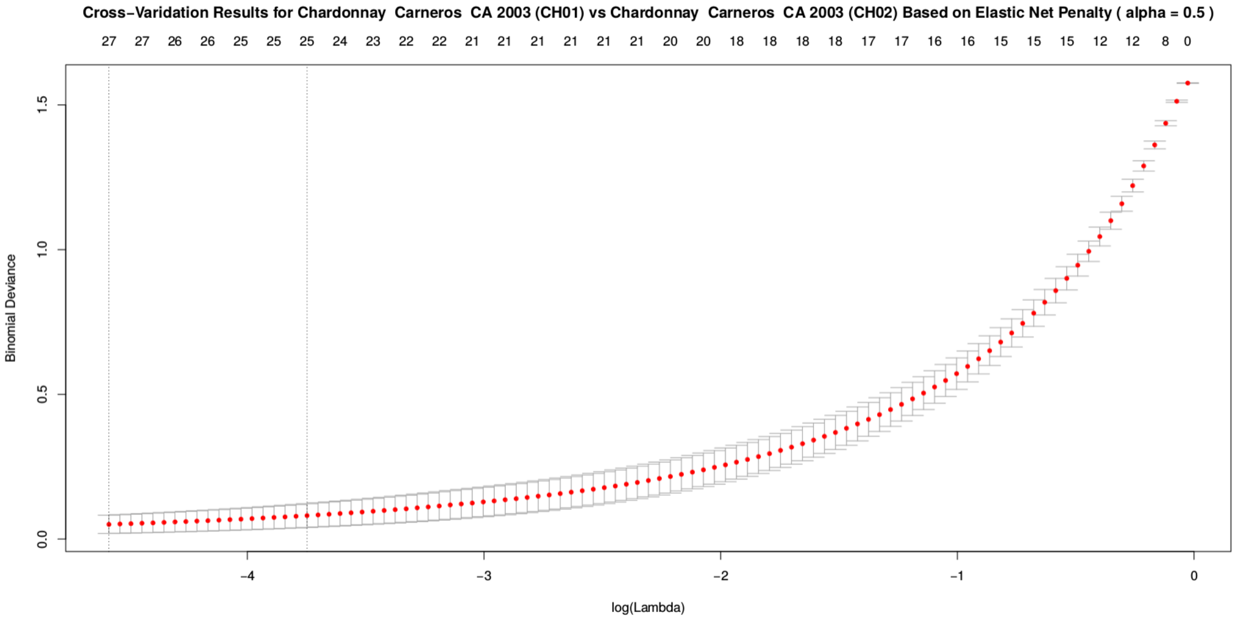

The value of the penalty parameter λ on the log scale is plotted along the x axis. The corresponding cross validation error (binomial deviance) is plotted along the y axis. The number of features that corresponds to the given value of penalty log(λ) is displayed on the top of the graph along the x-axis. The two vertical lines correspond to λ-s picked by the cross-validation algorithm. The first λ (dotted line on the left) corresponds to the smallest mean cross-validation error. The second λ (dotted line on the right) corresponds to the most regularized model with the error within one standard deviation from the minimum cross-validation error.

Linear Discriminant Analysis (LDA)

The tool performs linear discriminant analysis (LDA) of the data. LDA is a supervised method based on the projection of data in the linear subspace to achieve maximum separation between samples in different groups and minimum separation between samples within groups. The subspace dimension defines the number of components used to describe the variability within the data. Due to the LDA method specification, the subspace dimension must be less than the number of treatment groups. The user has an option to specify the dimension of the subspace directly (default = 2) or to perform single or double cross-validation to determine the dimension of the subspace. For single and double cross-validation, the dataset is split when model fit is performed. For double cross-validation, the data set is split into pieces and the model fit is performed on one piece using cross-validation and evaluated on the other pieces. For single cross-validation, the data are used to both fit and evaluate the model using a three-fold cross validation. Visual summaries are provided in the form of a 2D plot where samples are colored by group and plotted along the determined subspace components pairwise.

Linear Discriminant Analysis is performed using the "LinearDiscriminantAnalysis", "GridSearchCV", and "cross_val_score" functions from the scikit-learn package in Python.

Note: A minimum of 100 samples is required by the tool for single or double cross validation

Details about the LDA method can be found in:

Trevor J.. Hastie, Tibshirani, R. J., and Friedman, J. H. (2011). The elements of statistical learning: data mining, inference, and prediction. Springer. p106-119

Details about the double cross-validation method(s) are found in:

Cawley, Gavin C., and Nicola LC Talbot. "On over-fitting in model selection and subsequent selection bias in performance evaluation." Journal of Machine Learning Research 11, no. Jul (2010): 2079-2107.

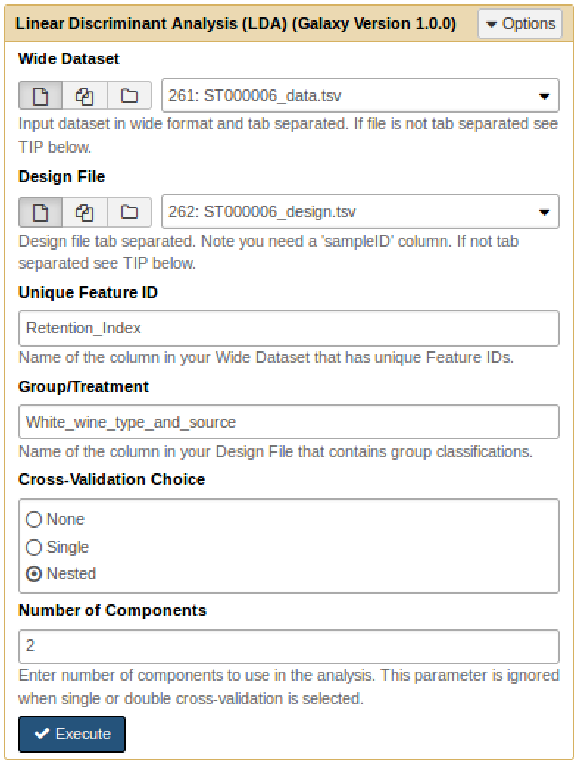

1. Select the **Wide Dataset** from the drop-down menu.
2. Select the **Design File** from the drop-down menu.
3. In the **Unique Feature ID** text box, type the name of the unique feature identifier.
4. In the **Group/Treatment** text box, type the name of the column in the **Design File** that identifies your groups.
5. Select **Cross-validation Options**. None corresponds to no cross-validation where the user specifies the number of components manually.
6. In the **Number of components** text box, type the number of components for the analysis to use (default = 2). This values should be less than the number of groups and is used only when the **Cross-validation Options** field is set to none.
7. Click **Execute**.

Output

This tool outputs:

(1) TSV file containing the components produced by the model for each sample.

Component_{i}: contains the score values for each sample. The number of levels {i} is specified in the Number of components text box or determined via cross validation.

(2) TSV file containing the sample classifications produced by the model.

Group_Observed: Initial group labels.

Group_Predicted: Predicted group labels.

(3) TSV file containing the classification accuracy (in percent) of the algorithm with respect to the number of correctly classified samples.

(4) A PDF file containing 2D plots for all pairwise comparisons of components. The samples are represented as dots and colored by treatment group.

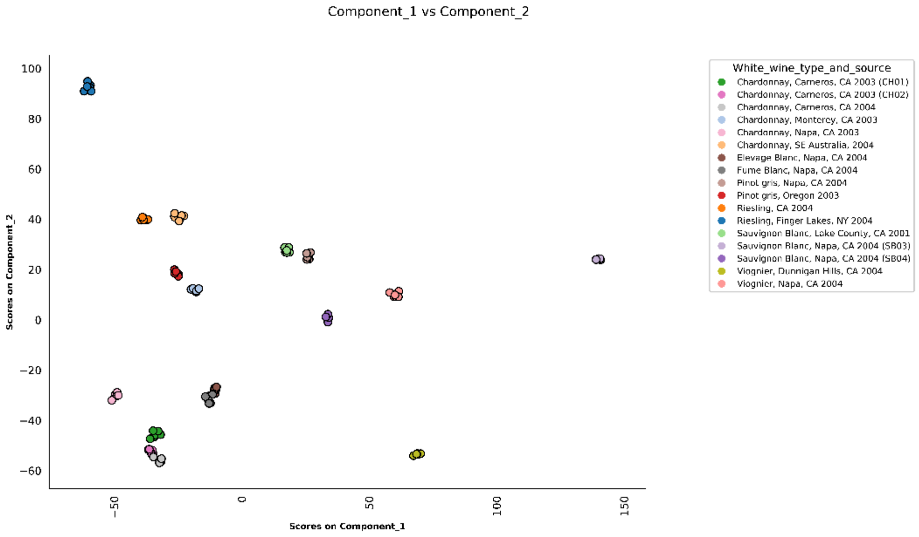

Scatterplot for the first two components for each sample. The samples are color coded based on the group they belong to.

Log and G-Log Transformation

This tool carries out either log or generalized log (g-log) transformation of values in a Wide Format dataset using the logarithm base specified by the user. The logarithmic transformation has the formula: $log(\, data\,)$ for each data cell and generalized logarithmic transformation has the formula: $log(\, data\, +\, \sqrt{data^2 + \lambda}\,)$ for each data cell. The generalized version becomes standard logarithmic transformation re-scaled by $\sqrt{2}$ if the λ value is 0. The value of λ is specified by the user. Three bases are available for both logarithmic transformations: base e (natural), base 2, and base 10.

Details about the generalized log and variance stabilizing transformations are in:

Huber, W., Von Heydebreck, A., Sueltmann, H., Poustka, A., and Vingron, M. (2002) Variance stabilization applied to microarray data calibration and to the quantification of differential expression, *Bioinformatics*, **18**, S96–S104.

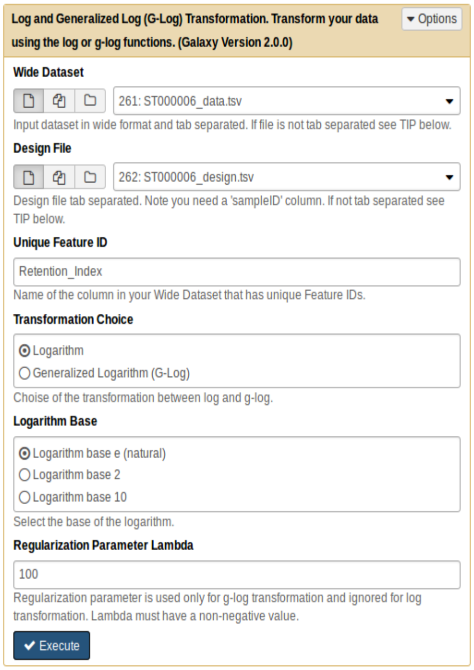

1. Select the **Wide Dataset** from the drop-down menu.
2. Select the **Design File** from the drop-down menu.
3. In the **Unique Feature ID** text box, type the name of the unique feature identifier.
4. Using the radio button, select the **Transformation Choice,** either Logarithm or Generalized Logarithm
5. Select the **Logarithm Base** using the radio button. Choices are the natural logarithm, logarithm base 2 and logarithm base 10.
6. In the **Regularization Parameter Lambda** text box, specify the regularization parameter lambda. Default value is 100.
7. Click **Execute**.

Output

This tool outputs one file:

- TSV file. Contains the same column and row names as the original **Wide Dataset** where the values in each cell correspond to the values obtained by either log or generalized og transformation procedure.

NOTE: If the original dataset has 0 or negative values before transformation they will be replaced with blank values after log function is applied since logarithms are not defined for non-positive values. Any values missing in the original dataset will remain missing.

Mass to Charge Ratio/Retention Time (m/z/RT) Matching

NOTE:This tool is primarily intended for matching mass spectrometry data processed using different parameter settings.

Each metabolite (feature) is characterized by a mass to charge (m/z) ratio and retention time (RT). After raw metabolomics data are processed (such as in mzMine), features are given internal identifiers (numbers) that are different for every run or set of parameters, making it very difficult to impossible to directly compare results across different parameter setting using the internal identifiers. However, it is possible to link internal identifiers using the m/z ratio and RT for each feature since changing parameter settings are predicted to result in only minor variations in m/z ratio and RT. This tool matches two MS datasets that correspond to different parameter settings. Each file should contain at least three columns: the m/z ratio, RT and internal identifier (feature ID). A feature matches across datasets if the m/z ratio and RT values in both MS files fall within a user defined window surrounding the m/z ratio and RT. The size of each window can be specified by the user where the final window width is two times the specified value.

NOTE: Since this is a 'many to many' merge where matching occurs within windows around the m/z ratio and the RT, a single internal identifier in one dataset can be matched with many identifiers in the other dataset.

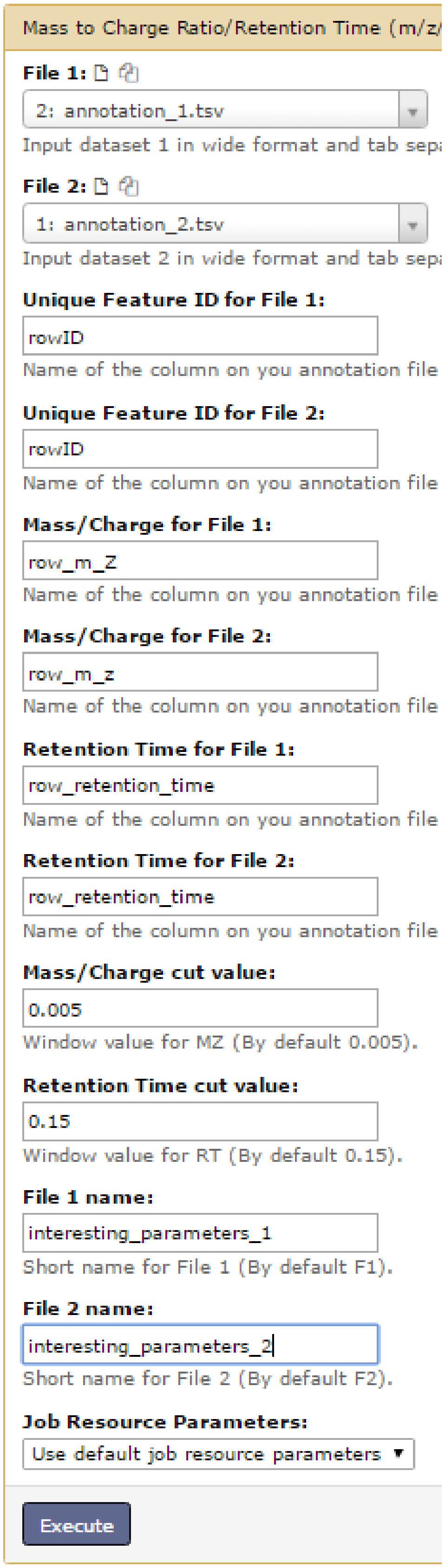

1. Select the **File 1** and **File 2** from their respective drop-down menus.
2. In the **Unique Feature ID for File 1** text box enter the name of the Unique identifier for **File 1.**
3. In the **Unique Feature ID for File 2** text box enter the name of the Unique identifier for **File 2**
4. In the **Mass/Charge for File 1** text box enter the name of the mass/charge column for **File 1.**
5. In the **Mass/Charge for File 2** text box enter the name of the Unique identifier for **File 2**
6. In the **Retention Time for File 1** text box enter the name of the retention time column for **File 1.**
7. In the **Retention Time for File 2** text box enter the name of the retention time column for **File 2.**
8. In the **Mass/Charge cut value** text box enter the window value for the m/z ratio. Accuracy of the method will change depending on this value. The default is 0.005.
9. In the **Retention Time Cut Value** text box enter the window value for the RT. Accuracy of the method will change depending on this value. The default is 0.15
10. In the **File 1 Name** text box type a short name or identifier for the **File 1.** This name will be use in the plot.
11. In the **File 2 Name** text box type a short name or identifier for the **File 2.** This name will be use in the plot.
12. Click **Execute**.

NOTE: While initially designed for MS data, this tool could also be used for other types of data where there is a need to match unique identifiers across datasets using values in two columns. A detection window set to zero (0) would provide an exact match.

Output

This tool outputs six files.

- TSV All peak combinations file. This file contains all combinations of possible features between **File 1** and **File 2.**
- TSV Matched peak combinations file. This file contains the features that match between **File 1** and **File 2.**
- TSV Unmatched peak combinations in file1. This file contains the features in **File 1** that do not have a match in **File 2.**
- TSV Unmatched peak combinations in file2. This file contains the features in **File 2** that do not have a match in **File 1.**

All four output files have the following 6 columns:

- ***Unique Feature ID for File 1.***
- ***Mass/Charge for File1.***
- ***Retention Time for File1***
- ***Unique Feature ID for file 2.***
- ***Mass/Charge for File2.***
- ***Retention Time for File 2.***

- TSV Summary file. This file contains a summary of the matching.
  - *UnmatchCombinations1*: Combinations of features in **File 1** that were not matched.
  - *UnmatchCombinations2*: Combinations of features in **File 2** that were not matched.
  - *MatchCombinations*: Matched combination of features between both files.
  - *AllCombinations*: All possible combination of features between both files.
  - *MatchFeatures1*: Features in file 1 with one or more matches in **File 2.**
  - *MatchFeatures2*: Features in file 2 with one or more matches in **File 1.**
  - *AllFeatures*: Total number of features between both files.
  - *MultipleFeatures1*: Features in file 1 that matched more than one feature n **File 2**.
  - *MultipleFeatures2*: Features in file 2 that matched more than one feature in **File1**.
  - *MultipleFeatures*: Total number of features with multiple matches for both files.
  - *SingleFeatures1*: Features in file 1 that matched only one feature in **File 2**.
  - *SingleFeature2*: Features in file 2 that matched only one feature in **File 1.**
  - *SingleFeatures*: Total number of features with only one match in the other file.

- PDF file. This file contains a set of 3 Venn diagrams that visualizes the matching.

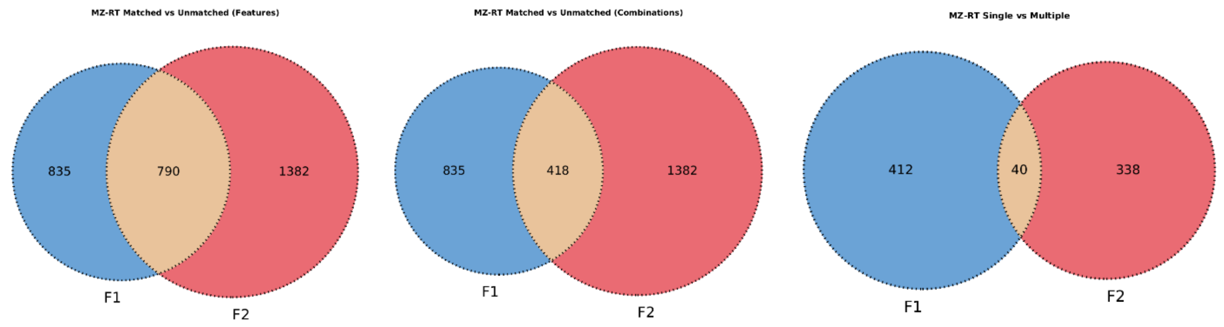

Venn diagram output produced by the Mass to Charge Ratio/Retention Time (m/z ratio RT) matching tool. Representation of the matched vs the unmatched features between **File 1** and **File 2** (left). Representation of the matched vs the unmatched combinations between **File1** and **File 2.** (center**).** Representation of the single vs multiple matches on the matched features (right).

Magnitude Difference Flags

This tool counts the number of digits before the decimal place for each feature in each sample. The tool identifies features with different orders of magnitude across different samples in the given group and produces corresponding flags. Unusual samples are identified by finding systematically low or high feature values for a particular sample.

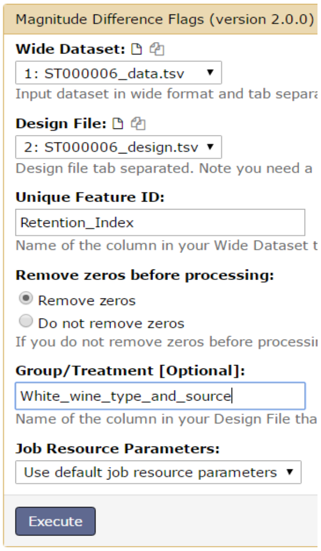

1. Select the **Wide Dataset** from the drop-down menu.
2. Select the **Design File** from the drop-down menu.
3. In the **Unique Feature ID** text box, type the name of the **Unique feature identifier**.
4. Choose whether you want to **Remove zeros before processing** by clicking on the radio button.
5. In the **Group/Treatment [Optional]** text box, type the name of the column in the **Design File** that identifies your groups.
6. Click **Execute**.

Output

The tool outputs a variable number of files: This tool will create from 2 to n+1 files. (where n is the number of groups for the **Group/Treatment [Optional]** parameter.

- TSV file. This flag file contains the following columns.
  - ***Unique Feature ID***
  - *Flag_feature_count_digits:* 0/1 flag where "1" is used to flag to features where the difference in the digit counts is greater than 2.

- TSV file: This file contains the digit counts for all samples or for the samples within groups depending on whether the **Group/Treatment[Optional]** parameter was provided.
  - ***Unique Feature ID***
  - **{SampleIDs}:** Every column contains a count of the digits corresponding to the values in the **Wide Dataset.** If the **Group/Treatment[Optional]** parameter is provided only the sampleIDs for the group will be included.

- A PDF file of the distribution of the digit counts within each group of samples.

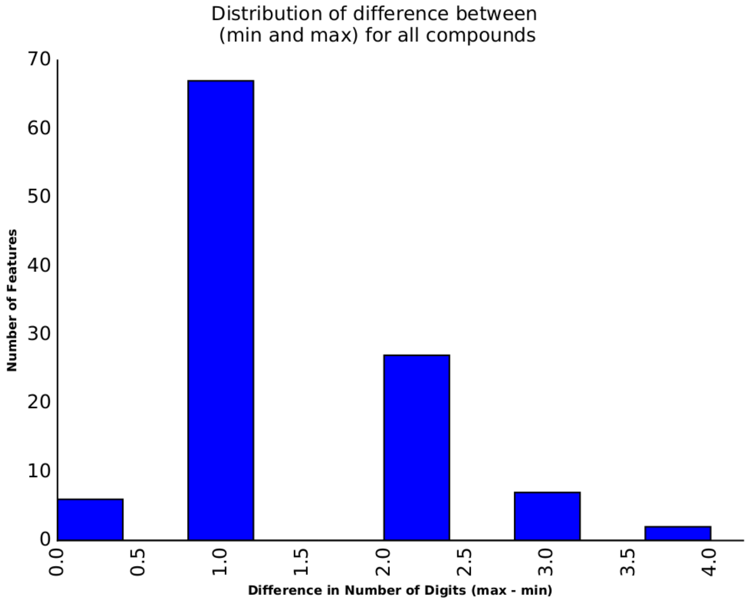

Histogram of the maximum differences between the largest and the smallest number of digits for a given feature across all samples. Differences larger than one might suggest that this feature is unstable.

Modify Design File

The tool creates a new design file based on an existing wide dataset and design file where the specified groups or samples are removed in the new design file.

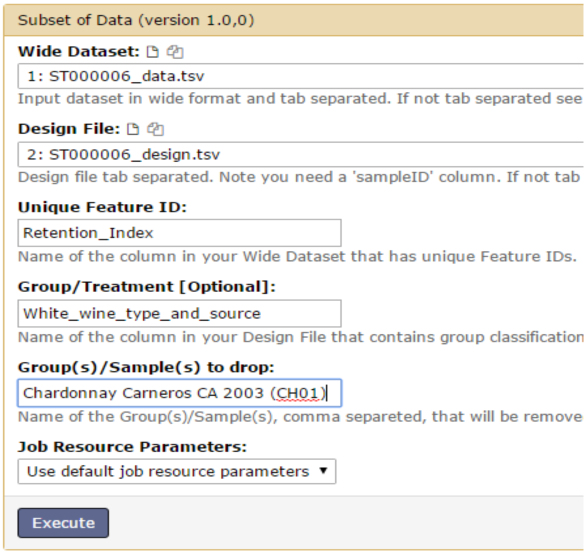

1. Select the **Wide Dataset** from the drop-down menu.
2. Select the **Design File** from the drop-down menu.
3. In the **Unique Feature ID** text box, type the name of the **Unique feature identifier**.
4. In the **Group/Treatment [Optional]** text box, type the name of the column in the **Design File** that identifies your groups. If left blank the dropping will be performed by sampleID.
5. In the **Group(s)/Sample(s) to drop** text box, type the names of the groups you want to **drop.** If more than one group is given, separate using commas (without spaces).
6. Click **Execute**.

Output

This tool will output one TSV file:

- A TSV **Design File** where the selected **Group(s)/Sample(s) to drop** have been removed. The remaining column names and corresponding values will be the same as in the original **Design File.**

Merge Flags

The tool performs a merge of two or more flag files. The flag files can be either in wide format or in design format. The merging requirements are that (1) the number of rows should be the same in all files being merged and (2) all files should contain the same **Unique ID** column name to merge by

Note: More broadly, the tool can merge non-metabolomics data files as long as the above requirements are met.

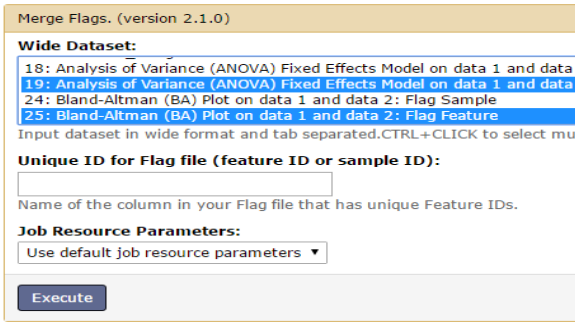

1. In the **Wide Dataset** box, select multiple flag datasets using CTRL+CLICK.
2. In the **Unique ID for Flag file (feature ID or sample ID)** text box, type the name of the column that contains either the unique feature IDs or unique sample IDs depending on what type of flag files are merged.
3. Click **Execute**.

Output

This tool outputs single file:

- TSV file. This file contains the columns from all the input **Wide Datasets** Flag Files.
  - ***Unique Feature ID***
  - *{Columnsfrom file 1+ columns from file 2+ … + columns file n}. For example:* If the columns in file one are ("column_a", "column_b") and the columns in file two are ("flag_a","flag_b") the columns in the result file will be ("column_a", "column_b", "flag_a","flag_b").

Note: If the input flag files have the same flag column name in multiple files the resulting, merged file will have columns from all imputed files. To distinguish columns obtained from different files the column names will be altered by adding the corresponding file name in the end of the column name. All non-supported file name characters will be changed to '_'.

Modulated Modularity Clustering (MMC)

Modulated Modularity Clustering method (MMC) was designed to detect latent structure of the variance-covariance matrix using weighted graphs. The method searches for optimal community structure and detects the magnitude of pairwise relationships. The optimal number of clusters and the optimal cluster size are selected by the method during the analysis.

The initial boundaries (lower and upper) for sigma as well as the number of points in the search grid are specified initially by the user. The boundaries are extended automatically by the algorithm if the values are close to the boundary. The correlation type (Pearson, Kendall or Spearman) can be specified.

Details about the method can be found in:

Stone, E. A., and Ayroles, J. F. (2009). Modulated modularity clustering as an exploratory tool for functional genomic inference. PLoS Genet, 5(5), e1000479.

MMC uses a python code developed by the authors and available under the GNU license using the link below:

http://mmc.gnets.ncsu.edu/

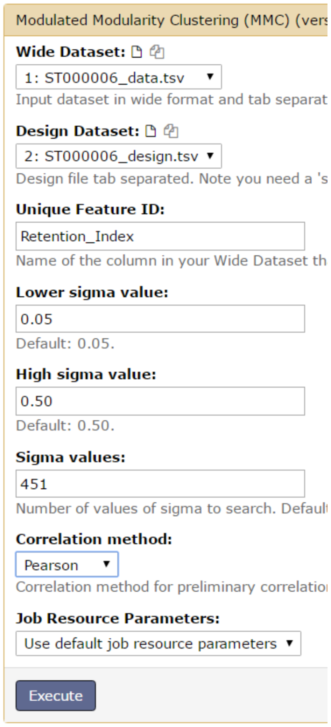

1. Select the **Wide Dataset** from the drop-down menu
2. In the **Unique Feature ID** text box, type the name of the **Unique feature identifier**.
3. In the **Lower sigma value** text box, type the decimal lower sigma value from 0 to 1 range. The default value is 0.05.
4. In the **High sigma value** text box, type the decimal upper sigma value from 0 to 1 range. The default value is 0.50. **High sigma value** has to be bigger than **Lower sigma value** for the algorithm to work.
5. In the **Sigma values** text box, type the number of sigma values considered. The default is 451. Higher numbers increase the precision but decrease the performance time.
6. Select the **Correlation method** from the drop-down method. (Pearson-standard correlation coefficient, default value, Kendall-Kendall Tau correlation coefficient, Spearman-Spearman rank correlation).
7. Click **Execute**.

Output

This tool outputs four files:

- TSV file. This file contains the algorithm summaries, in the following columns:
  - *Gene:* Contains the names of the rows of the input **Wide Dataset.**
  - Module: Contains the module number for each feature calculated in the script.
  - Entry Index: Contains the original order of the names of the rows of the input **Wide Dataset.**
  - Degree: Average of the absolute values of correlations for the given element in the block to other elements within that block.
  - Average Degree: Average values of the degrees computed above across all elements within the given block.
- A PDF containing unsorted, sorted, and sorted-smoothed heatmaps of the variance-covariance matrixes.

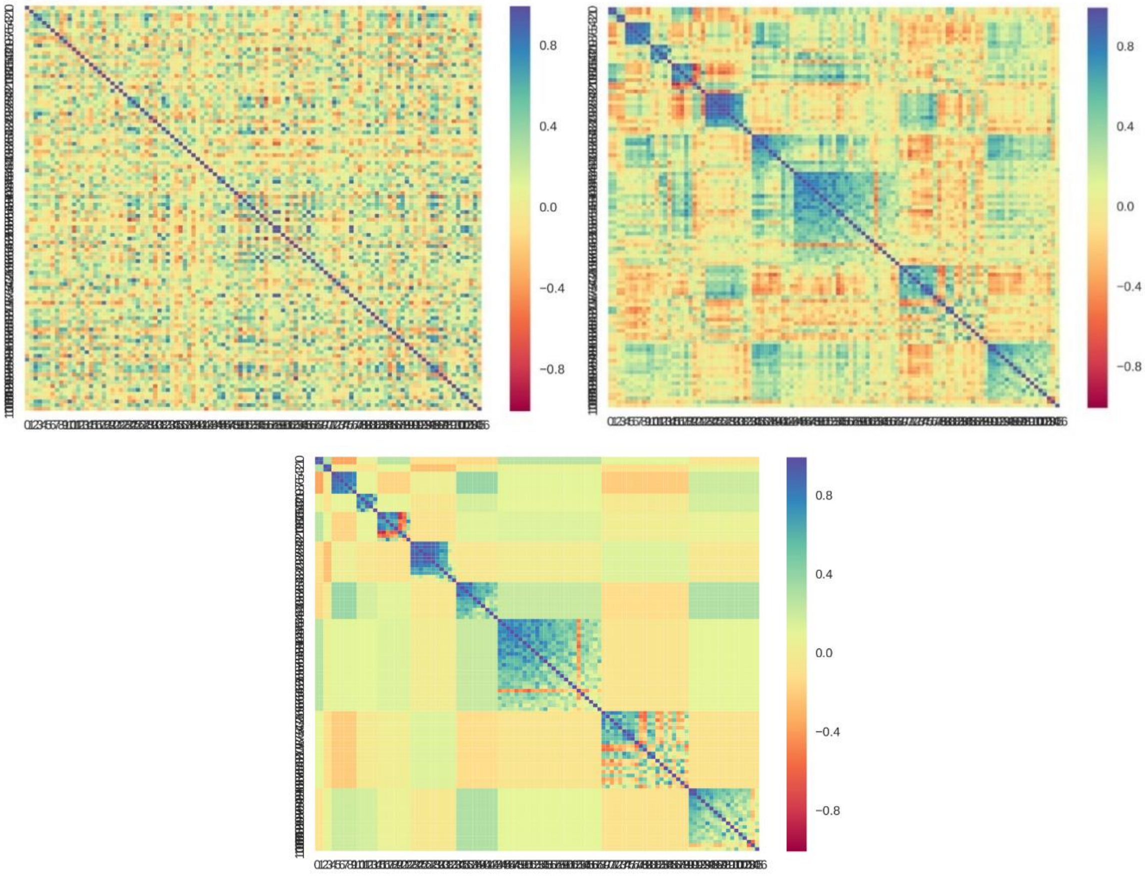

The heatmaps show the original variance-covariance matrix (top left), the rearranged variance-covariance matrix (top right) and the smoothed version of the rearranged variance-covariance matrix (bottom).

Multiple Testing Adjustment (MTA)

The tool is designed to adjust a column of p-values for multiple comparison using three different methods. Three adjustment methods have been implemented; Bonferroni based on the family-wise error rate, Benjamini/Hochberg (BH) and Benjamini/Yekutieli (BY) based on the false discovery rate. The tool produces a table containing columns with the *p*-values for each adjustment method used.

Multiple Testing Adjustment is performed using the "multipletests" function from the statsmodels package in Python.

Details about the PH and BY methods are available in the papers below:

Benjamini, Y., & Hochberg, Y. (1995). Controlling the false discovery rate: a practical and powerful approach to multiple testing. Journal of the royal statistical society. Series B (Methodological), 289-300.

Benjamini, Y., & Yekutieli, D. (2001). The control of the false discovery rate in multiple testing under dependency. Annals of statistics, 1165-1188.

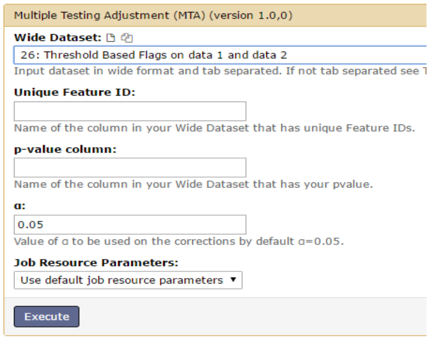

1. Select the **Wide Dataset** from the drop-down menu.
2. In the **Unique Feature ID** text box, type the name of the **Unique feature identifier**.
3. In the **p-value column** text box, type the name of the column in your **Wide Dataset** that contains *p*-values.
4. In the **α** text box, type the value to be used for multiple correction. Default **α** =0.05.
5. Click **Execute**.

Output

This tool outputs two files:

- A TSV results file containing the columns:
  - {**p-value column}:** The values in this column are the same as in the **p-value column** from the **Wide Dataset.**
  - {**p-value column**}_bonferroni: results of Bonferroni correction on **p-value column** from the **Wide Dataset.**
  - {**p-value column**}_bHochberg: results of Benjamin/Hochberg correction on **p-value column** from the **Wide Dataset.**

    - {**p-value column**}_bYekutieli: results of Benjamin/Yekutieli correction on **p-value column** from the **Wide Dataset.**
- A TSV flag file containing the following columns (non-significant values are flagged "1"):
    - flag_{**p-value column**}_bonferroni_off: 0/1 flag where "1" represents those features in which the p-value after the Bonferroni correction are smaller than **α.**
    - flag_{**p-value column**}_bHochberg_off: 0/1 flag where "1" represents those features in which the p-value after the Benjamin/Hochberg correction are smaller than **α.**
    - flag_{**p-value column**}_bYekutieli_off: 0/1 flag where "1" represents those features in which the p-value after the Benjamin/Yekutieli correction are smaller than **α.**

Partial Least Squares Discriminant Analysis (PLS-DA)

The tool performs partial least square discriminant analysis (PLS-DA) for the two treatment groups selected. The subspace dimension defines the number of components that will be used to describe the variability within the data. The subspace dimension is specified in the range of two to the number of sample in the two selected groups. The user has the option to specify the dimension of the subspace directly (Default=2) or to perform single or double cross-validation to determine the dimension of the subspace. For double cross-validation the data set is split into pieces and the model fit is performed on one piece using cross-validation and evaluated on the other pieces. For single cross-validation using three-fold cross validation is used.

PLS-DA is performed using the “PLSRegression”, GridSearchCV, and “cross_val_score” functions from the scikit-learn package in Python.

NOTE: A minimum of 100 samples is required by the tool for single or double cross validation.

Details about the PLD-DA method are available via references:

Geladi, Paul, and Bruce R. Kowalski. "Partial least-squares regression: a tutorial." Analytica chimica acta 185 (1986): 1-17.

Details about the cross-validation method(s) are available via the reference and link:

Cawley, Gavin C., and Nicola LC Talbot. "On over-fitting in model selection and subsequent selection bias in performance evaluation." Journal of Machine Learning Research 11, no. Jul (2010): 2079-2107.

NOTE: The user has to insure that group names themselves do not contain commas or spaces. The separator for the two groups should only include comma and no extra spaces.

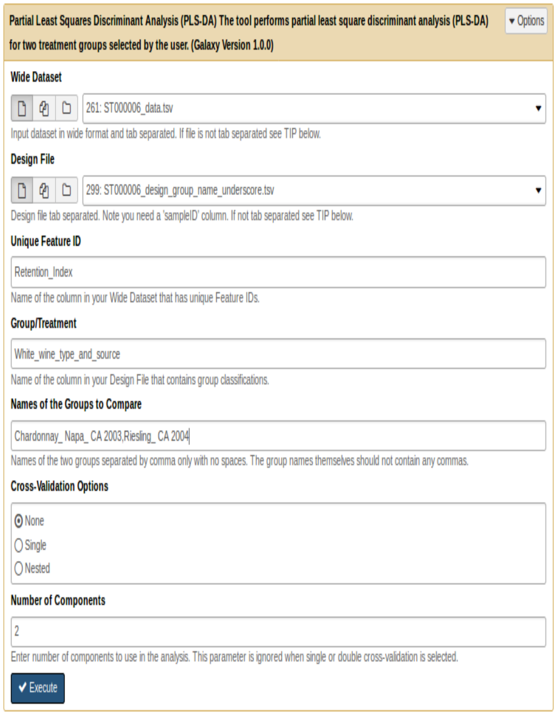

1. Select the **Wide Dataset** from the drop-down menu.
2. Select the **Design File** from the drop-down menu.
3. In the **Unique Feature ID** text box, type the name of the unique feature identifier.
4. In the **Group/Treatment** text box, type the name of the column in the **Design File** that identifies your groups.
5. In the **Name of the Groups to Compare** text box, type the names of the two groups to compare. The user has to insure that group names themselves do not contain commas. The separator for the two groups should only include comma and no extra spaces.
6. Select **Cross-validation Options**. None corresponds to no cross-validation. Nested corresponds to nested cross-validation
7. In the **Number of components** text box, type the number of components for the analysis to use (default = 2). This field is used only when **Cross-validation Options** field is set to none.
8. Click **Execute**.

Output

This tool outputs three files:

- TSV file. This file contains the scores produced by the model for each sample.
  - ***SampleID***
  - ***LV_{i}***: Each column contains the score values for each sample the amount of levels is specified in the **Number of components** text box or determined via CV.
- TSV file. This file contains the weights produced by the model for each feature.
  - ***Unique Feature ID***
  - ***LV_{i}:*** Each column contains the weight value for each feature, the amount of levels is specified in the **Number of components** text box.
- TSV file. This file contains the classification produced by the model for each sample.
  - ***SampleID***
  - ***Group_Observed:*** Initial group labels using 0-1 binary codes.
  - ***Group_Predicted:*** Predicted group labels in integers.
  - ***Group_Predicted_Rounded:*** Predicted group labels that were rounded to match the observed group binary classification.
- TSV file. This file contains the classification accuracy in percent between the classes.

A PDF file containing the 2D plots for all pairwise comparisons of components between the two treatment groups.

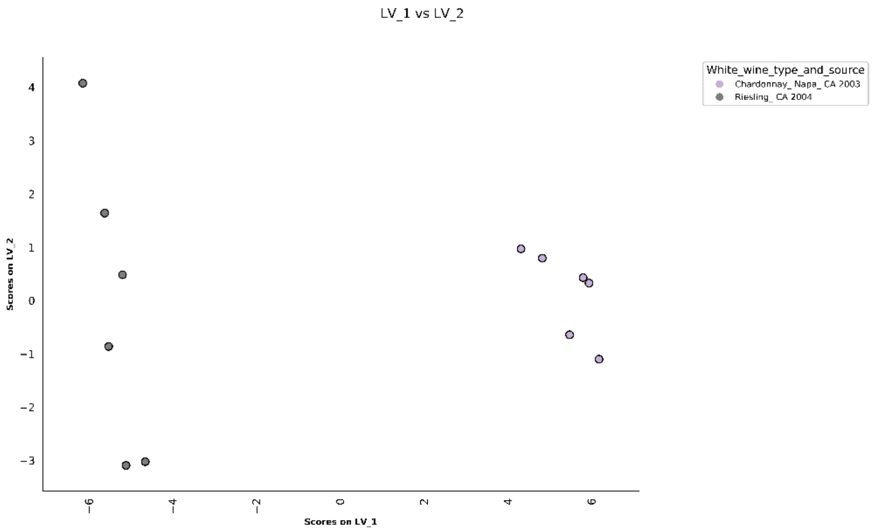

Penalized Mahalanobis Distance (PMD)

The Penalized Mahalanobis distance (PMD) tool can be used to compare samples within a group and to accounts for the correlation structure between metabolites. In contrast, Standardized Euclidian distance (SED) relies solely on geometric distance and ignores any dependency structures between features. PMD incorporates the correlation structure inside the distance measurement.

When correlation structure and dependency between metabolites are ignored, the features inverse variance-covariance matrix simplifies to a diagonal matrix with diagonal values. In this case, MD simplifies to SED. When the number of features is greater than the number of samples, the inverse of the features variance-covariance matrix does not exist. This is the case for most -omic data. Here, the inverse is estimated using a regularization method (Archambeau et al. 2004).

Details of the regularization algorithm can be found in Supplementary file 3 in Kirpich et al. 2017 and using the references.

Hoerl, Arthur E., and Robert W. Kennard. "Ridge regression: Biased estimation for nonorthogonal problems." Technometrics 12, no. 1 (1970): 55-67.

Archambeau C, Vrins F, Verleysen M. Flexible and Robust Bayesian Classification by Finite Mixture Models. InESANN 2004 (pp. 75-80).

NOTE: Groups with less than three samples will be excluded from the analysis.

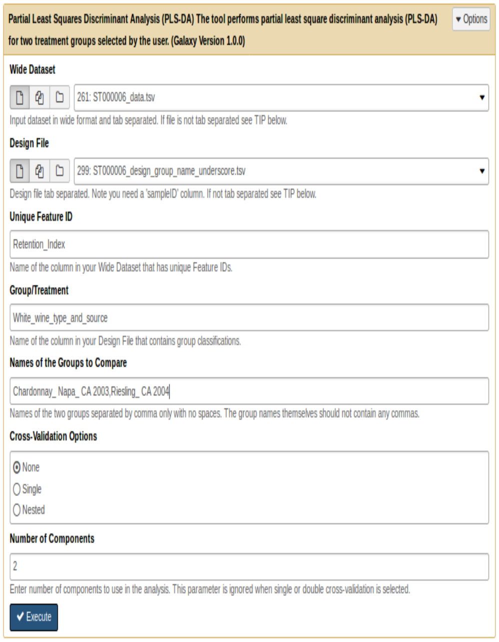

1. Select the **Wide Dataset** from the drop-down menu.
2. Select the **Design File** from the drop-down menu.
3. In the **Unique Feature ID** text box, type the name of the **Unique feature identifier**.
4. In the **Group/Treatment [Optional]** text box, type the name of the column in the **Design File** that identifies your groups.
5. In the **Input Run Order Name [Optional]** text box, type the name of the column in your **Design File** that has the Run Order variable.
6. In the **Additional groups to separate by [Optional]** text box, type the name of additional columns in your **Design File** to separate your data by.

7. In the **Threshold** text box, type the threshold, specified as a percentile, to be used for the distribution of the distance values. The default is 0.95.
8. In the **λ Penalty** text box, type the percentile to be used on the calculation of the penalized variance-covariance matrix.
9. Click **Execute**

Output

This tool outputs three different files:

- TSV file. This file contains the distances from each sample to the estimated mean.
    - **sampleID**
    - *distance_to_mean:* distance from the sample to the estimated mean. If **Group/Treatment [Optional]** is given then distances will be calculated from each sample to the estimated group mean.

- TSV file. This contains a n x n matrix (where n is the number computed samples) of the pairwise distances between the samples. If the **Group/Treatment [Optional]** variable is specified, the distances will be computed within the groups.
    - **sampleID**

- A PDF file containing
    - Boxplots of the distribution of distances.

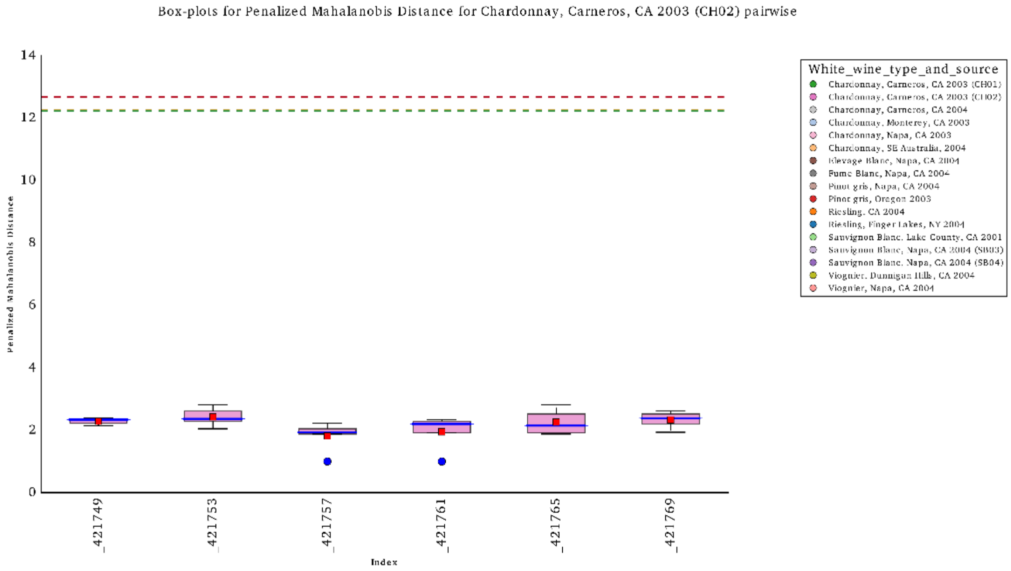

Penalized Mahalanobis distances are computed pairwise for each sample within a group. All pairwise distances computed are summarized for each sample as boxplots. Potential outliers (blue dots), means (red squares), and median (dark blue bars) are displayed. The threshold to declare a potential outlier a threshold (specified as a percentile) in the input. This example shows the Standardized Euclidean Distances in the "Chardonnay, Carneros, CA 2003 (CH02)" group. The dashed lines correspond to the cutoffs are computed from beta, normal and chi-squared distributions in red, yellow and green respectively. Please note that normal and chi-squared cutoffs are expected to be very close to each other.

- 2D scatter plots that show the distances

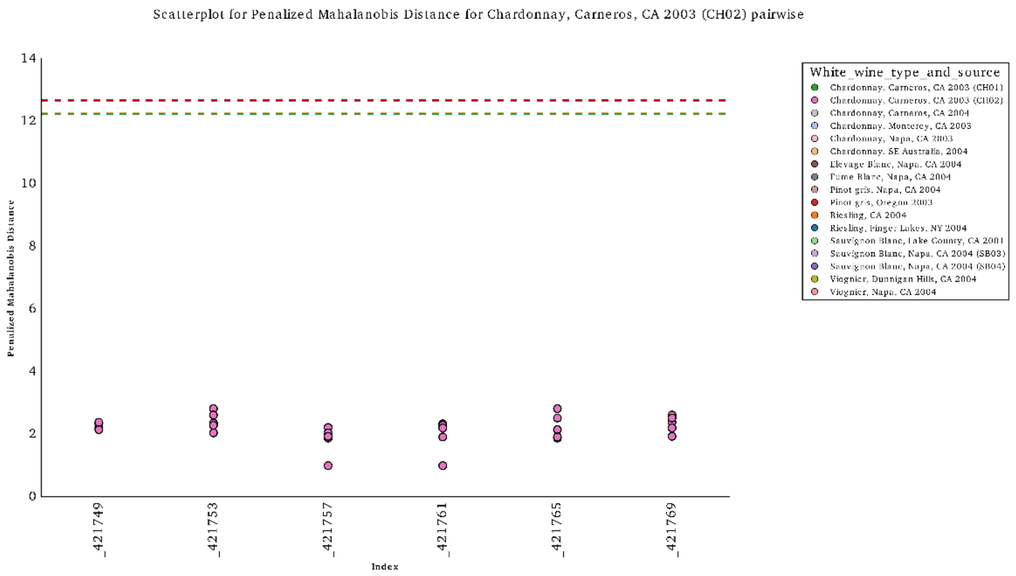

A scatterplot of the Penalized Mahalanobis Distances in the “Chardonnay, Carneros, CA 2003 (CH02)” group. Distances are computed pairwise between samples within a group. The dashed lines correspond to the cutoffs are computed from beta, normal and chi-squared distributions in red, yellow and green respectively. Please note that normal and chi-squared cutoffs are expected to be very close to each other.

Principal Component Analysis (PCA)

The tool performs principal component analysis (PCA) of the data. Visual summaries are provided in the form of 2D and 3D scatter plots for the first three principal components. The samples in the scatter plots are colored based on group classifications.

PCA is performed using the "PCA" function from the scikit-learn package in Python.

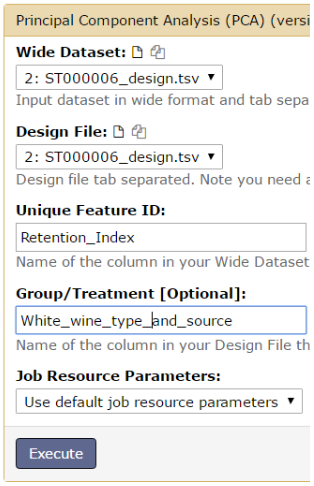

1. Select the **Wide Dataset** from the drop-down menu .
2. Select the **Design File** from the drop-down menu.
3. In the **Unique Feature ID** text box, type the name of the **Unique feature identifier**.
4. In the **Group/Treatment [Optional]** text box, type the name of the column in the **Design File** that identifies your groups.
5. Click **Execute**.

Output

This tool outputs three files:

- TSV file. This file contains the loadings for each **Unique Feature ID** in the **Wide Dataset**.
    - ***Unique Feature ID***
    - ***PC{i}:*** For each component, contains the eigenvectors/variable loadings for each feature.

- TSV file. This file contains the scores for each **sampleID** in the **Wide Dataset**.
    - ***SampleID***
    - ***PC{i}***: Each column contains the scores for each component/sample.

- A PDF file of scatter plots of the first three principal components.

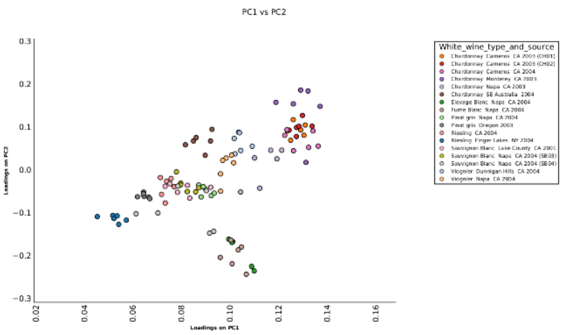

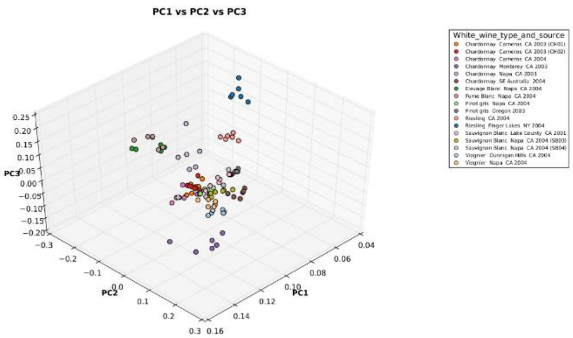

The samples are color coded based on group classifications. Scatterplot of the first two principal components (left) and 3D scatterplot shows the first three components (right).

Random Forests (RF)

The tool identifies features that are different between treatment groups based on the random forest (RF) algorithm. Based on Classification and Regression Trees (CART), random forests are an ensemble learning method for classification, regression and variable importance evaluation.

Random Forest classification is performed using the “RandomForestClassifier” function from the scikit-learn package in Python.

Details about the algorithm can be found in the book:

Breiman, L. (2001). Random forests. Machine learning, 45(1), 5-32.

NOTE: The use of machine learning algorithms (including random forest) on datasets with a small number of samples is controversial.

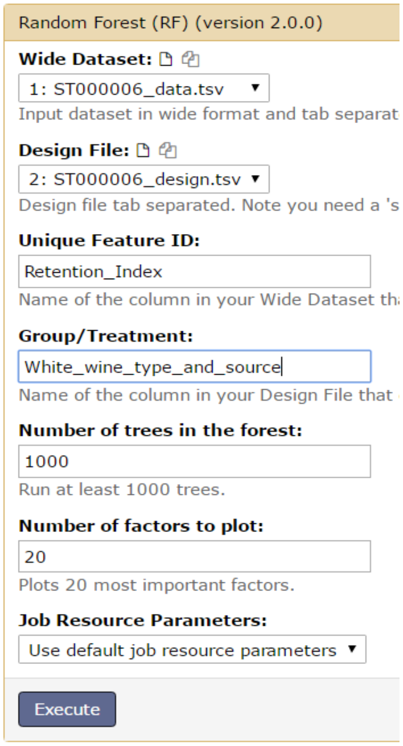

1. Select the **Wide Dataset** from the drop-down menu.
2. Select the **Design File** from the drop-down menu.
3. In the **Unique Feature ID** text box, type the name of the **Unique feature identifier**.
4. In the **Group/Treatment** text box, type the name of the column in the **Design File** that identifies your groups.
5. In the **Number of trees in the forest** text box, type the number of trees that you want to have in your forest (default is 1000)
6. In the **Number of factors to plot** text box, type the number of factors that you want to plot on the Variable Importance plot (default 20).
7. Click **Execute**.

Output

This tool will always output three different files:

- TSV file. This file contains the **Unique Feature IDs** from the **Wide Dataset** ranked in order of relative importance,

- *Unique feature ID*
- *ranked_importance:* importance value for each feature as calculated by the random forest method.

- TSV file. This file contains the ranked features from the **Wide Dataset** saved in columns in the order that corresponds to their relative importance.
    - ***SampleID***
    - ***Group/Treatment***
    - ***{Unique feature ID}:*** Sorted unique feature IDs according to the relative importance of the variable
- a PDF file with the variable importance plot.

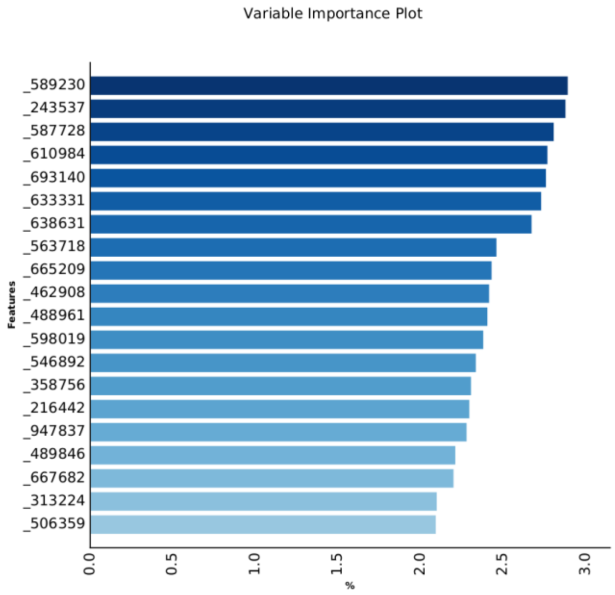

The variable importance plot displays the 20 most important features based on the random forest algorithm. The color of each feature changes from the most important among the 20 (dark blue) to the least important among the 20 (light blue).

Remove Selected Features or Samples

The tool removes features (rows) or samples (columns) from a wide format dataset based on the flags in a separate flag file. The user specifies a flag file and a flag column name to indicate removal. Features or samples with a flag value equal to, greater than, or less than a user specified Cutoff Value (Default = 1) will be removed from the *wide dataset*. The flag file should be either a wide format flag file (used for dropping features) or design format flag file (used for dropping samples). The difference between the flag file formats is described in the beginning of this manual.

NOTE: Flag files generated outside of SECIM Tools can be used for feature removal.

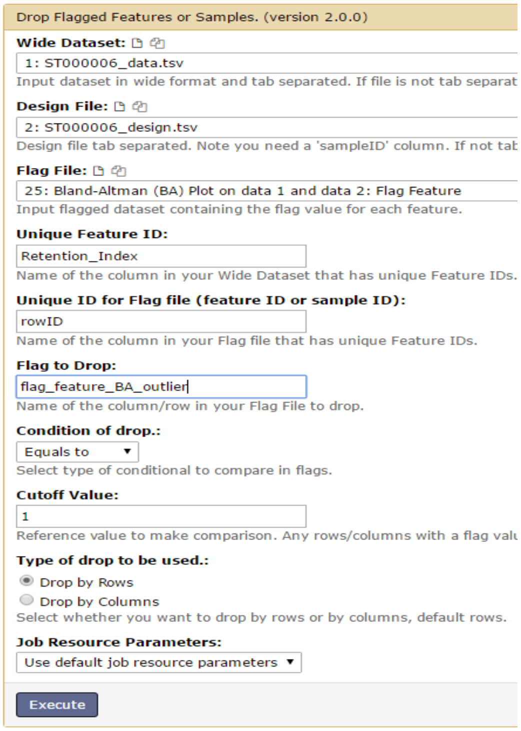

1. Select the **Wide Dataset** from the drop-down menu.
2. Select the **Design File** from the drop-down menu.
3. Select the **Flag File** from the drop down menu. The **Flag File** should be either a **Wide Format** flag file used for features or a **Design Format** flag file used for samples. More details about flag file formats are available in the Data Format section of this manual.
4. In the **Unique Feature ID** text box, type the name of the **Unique feature identifier**.
5. In the **Unique ID for Flag file (feature ID or sample ID)** text box, type the name of the column in the **Flag File** that contains the **Unique Feature ID** (or **Sample ID**.)
6. In the **Flag to Drop** text box, type the name of the column in the **Flag File** that contains the flag values to use to perform the drop.
7. Select the **Condition of drop** from the drop-down menu.
8. In the **Cutoff Value** text box, type the value to be used as a cutoff. Features or samples with a flag value {equal to, greater than, or less than} this value (Default = 1) will be dropped from the wide dataset.
9. Choose the **Type of drop to be used** by clicking the radio button.
10. Click **Execute**

NOTE: The choice for the type of drop used depends on the flag file: for wide format flag file the row option should be utilized while for design format flag file column option should be utilized.

Output

This tool outputs two files:

- A TSV **Wide Dataset** obtained from the input **Wide Dataset** where the flagged features (row) or samples (columns) have been removed. The remaining column names and corresponding values will be the same as in the original **Wide Dataset.**

- A TSV **Flag File** obtained from the input **Flag File** where flag values for the remaining features (or samples) are saved. The remaining column names and corresponding values will be the same as in the original **Flag File.**

Retention Time (RT) Flags

NOTE: This tool is primarily intended for flagging features with variation in retention times in mass spectrometry data analysis. The goal of the tool is to identify potential problems with the instrument or with data processing and pre-processing.

The retention time for a given feature is predicted to be relatively consistent across samples. This tool identifies potential abnormalities or shifts in the retention time for a feature. The tool uses multiple criteria to identify feature's discrepancies.

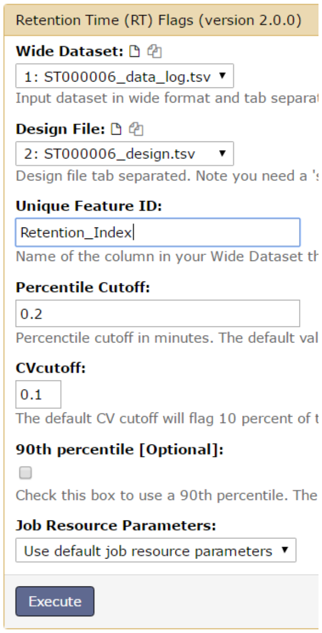

1. Select the **Wide Dataset** from the drop-down menu .
2. Select the **Design File** from the drop-down menu.
3. In the **Unique Feature ID** text box, type the name of the **Unique feature identifier**.
4. In the Coefficient of Variation **(CV) cutoff** text box, enter the coefficient of variation threshold to flag the top X% of features with the largest CV's. The default value is 0.1 (90th percentile).
5. In the **Value Cutoff** text box, enter the threshold for the RT difference in minutes. The default value is 0.2 minutes.

6. Click the **90th percentile [Optional]** checkbox to flag features where the RT difference between the 10th and 90th percentiles is greater than the **Percentile Cutoff** (default uses the 5th/95th percentiles).
7. Click **Execute**.

Output

The tool outputs two files:

- TSV file. This file contains flags for each feature, it has the following columns:
    - ***Unique Feature ID***
    - *flag_RT_Q95Q05_outlier:* 0/1 flag where the value "1" is for features where the difference in the retention time between the 95th and 5th percentile (customizable to 90th and 10th percentiles) is greater than the user-specified threshold in minutes (default is 0.2 minutes).
    - *flag_RT_max_gt_threshold:* 0/1 flag where the value "1" is for features where the difference between the retention time maximum and median is greater than user-specified threshold in minutes/2 (default is 02/2 = 0.1 minutes).

- *flag_RT_min_lt_threshold:* 0/1 flag where the value "1" is for features where the difference between the retention time minimum and median is greater than user-specified threshold in minutes/2 (default is 02/2 = 0.1 minutes)..
- *flag_RT_min_max_outlier:* 0/1 flag where the value "1" is for features where the retention time minimum or maximum is greater than 3 times the standard deviation away from the mean.
- *flag_RT_big_CV*: 0/1 flag where the value "1" is for features whose coefficient of variation (CV) in retention time is greater than some threshold percentile of all the features. The default value is 0.1 which corresponds to 90th percentile.

- PDF file. This file contains a density plot of the coefficients of variation (CV) for the retention time.

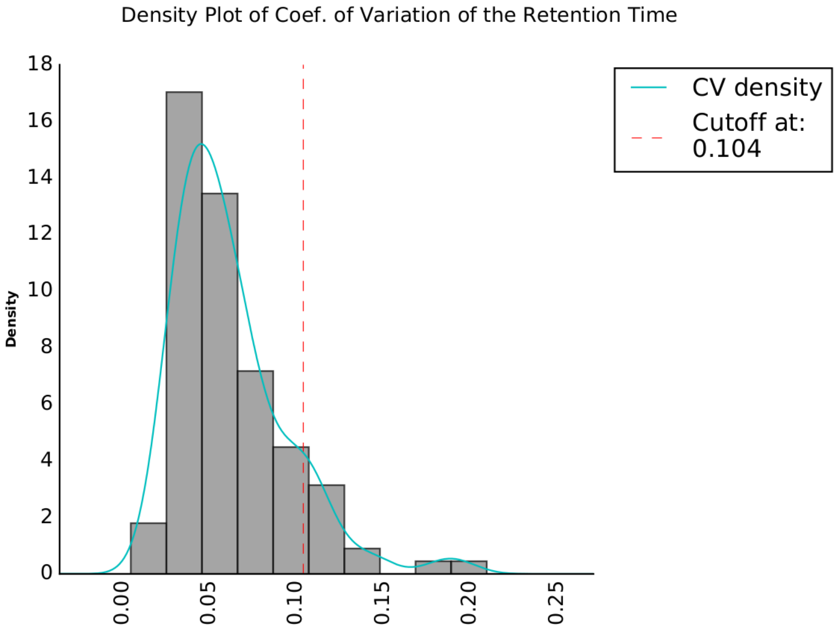

The histogram and smoothed density plot of the coefficients of variation for retention time. The red dotted line shows the cutoff for the top 10 % of the data.

Run Order Regression (ROR)

NOTE: The tool is intended to evaluate the impact of sample run order on feature (row) values. Not applicable in the absence of known run order.

This tool fits a simple linear regression by feature (row) using values for each feature as a response and sample run order as a linear predictor. The goal is to identify a linear trend that can change over time and whether the trend's slopes are significantly different from zero. The tool generates flags if the slope is statistically significant for two different levels of statistical significance ($\alpha = 0.05$ and $\alpha = 0.01$).

Run Order Regression model fits are performed using the "ols" function from the statsmodels package in Python.

NOTE: Groups with one element are excluded from the analysis.

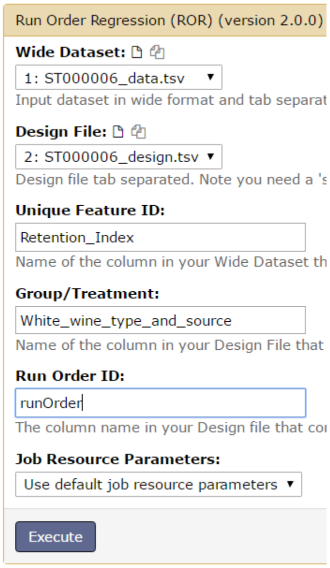

1. Select the **Wide Dataset** from the drop-down menu.
2. Select the **Design File** from the drop-down menu.
3. In the **Unique Feature ID** text box, type the name of the **Unique feature identifier**.
4. In the **Group/Treatment [Optional]** text box, type the name of the column in the **Design File** that identifies your groups
5. In the **Run Order ID** text box, type the name of the column in your **Design File** that has the Run Order variable.
6. Click **Execute**

Output

This tool outputs three different files:

- TSV file. This file contains the regression summaries with the following columns:
    - *pval*: p-value of the regression.
    - *rsq*. **R^2** values of the regression.
    - *slope*: values of the regression slope.

- a TSV flag file with the following columns.

- *flag_feature_runOrder_pval_01*: 0/1 flag for each feature where the value “1” is assigned for p-values smaller than 0.01.
- *flag_feature_runOrder_pval_05*: 0/1 flag for each feature where the value “1” is assigned for p-values smaller than 0.05.

- a PDF file with fitted regression plots for each significant feature.

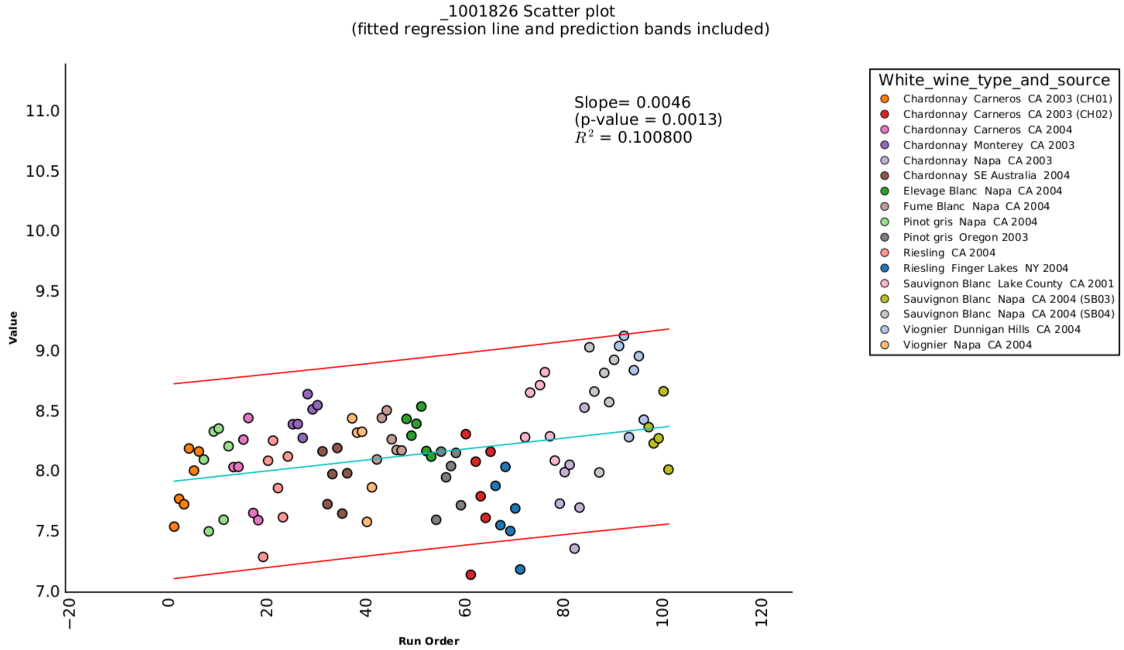

This plot shows the color-coded scatterplot of the values of “_1001826” feature for all samples. The run order of the samples is displayed along the x-axis and the feature value is displayed on the y-axis. Each color represents a different group of samples. A regression line has been fit and the R^2 Is displayed on the plot. If there is no effect of run order, the slope of the regression line will be zero. A test of the null hypothesis that the slope is zero is performed and the resulting p-value. The fitted line (light blue) and the 95% confidence bands (red) are displayed on the graph.

Scatter Plot 2D

The tool provides a 2D scatter plot of the points saved in a Long Format file. If coloring by group is desired the column with the sample names in the Long Format dataset has to have the name "sampleID" to match the name in the Design File. Scatter plot 2D allows the user to plot any pair of scores output from the Principal Component Analysis (PCA) or other data uploaded independently.

NOTE: The user should verify that the Long Format and Design File datasets have no missing values.

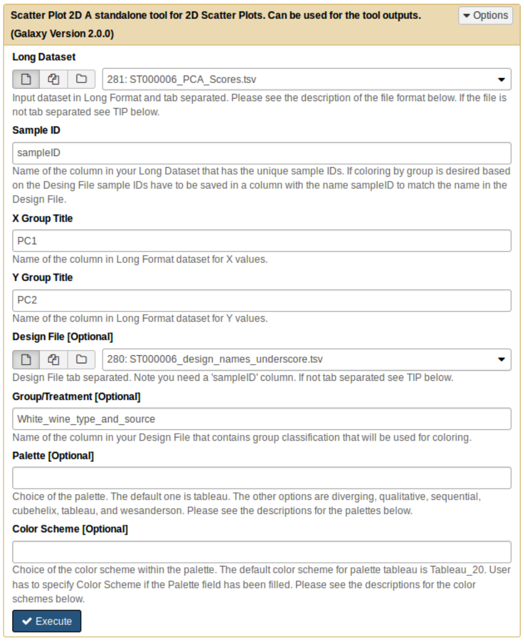

1. Select the **Long Dataset** from the drop-down menu. The scores in the PCA tool are output in the long format or the user can upload another data set.
2. In the **Sample ID** text box, type the name of the **Sample ID** column from the **Long Format** dataset. In order for the coloring to work correctly this name should be the same as in the **Design File** i.e. the name for the samples column should be **sampleID.**
3. In the **X Group Title** text box, type the name the column name from your **Long Format** that you want to use for X axis.
4. In the **Y Group Title** text box, type the name the column name from your **Long Format** that you want to use for Y axis.
5. Select the **Design File [Optional]** from the drop-down menu. The Design File should have a **Group/Treatment** grouping variable column that will be used for sample's coloring.
6. In the **Group/Treatment [Optional]** text box, type the name of the column in the **Design File** that contains the group classifications.
7. In the **Palette [Optional]** text box, specify the palette. Default = tableau. The other options are diverging, qualitative, sequential, cubehelix, tableau, and wesanderson. Please see the descriptions for the palettes below.
8. In the **Color Scheme [Optional]** specify the color scheme within the **Palette**. The default color scheme for palette tableau is Tableau_20. User has to specify **Color Scheme** if the **Palette** field has been filled. Please see the descriptions for the color schemes below.
9. Click **Execute**.

The user has an option to specify the palette and the color scheme within the palette. If the palette is specified the color scheme has to be specified. The list of available palettes is diverging,

qualitative, sequential, cubehelix, tableau, and wesanderson. The lists of corresponding color schemes available within each palette are available from:

https://jiffyclub.github.io/palettable/tableau/
https://jiffyclub.github.io/palettable/colorbrewer/diverging/
https://jiffyclub.github.io/palettable/colorbrewer/qualitative/
https://jiffyclub.github.io/palettable/colorbrewer/sequential/
https://jiffyclub.github.io/palettable/cubehelix/
https://jiffyclub.github.io/palettable/wesanderson/

Output

This tool outputs one file:

- A PDF file containing a 2D scatterplot of the selected data.

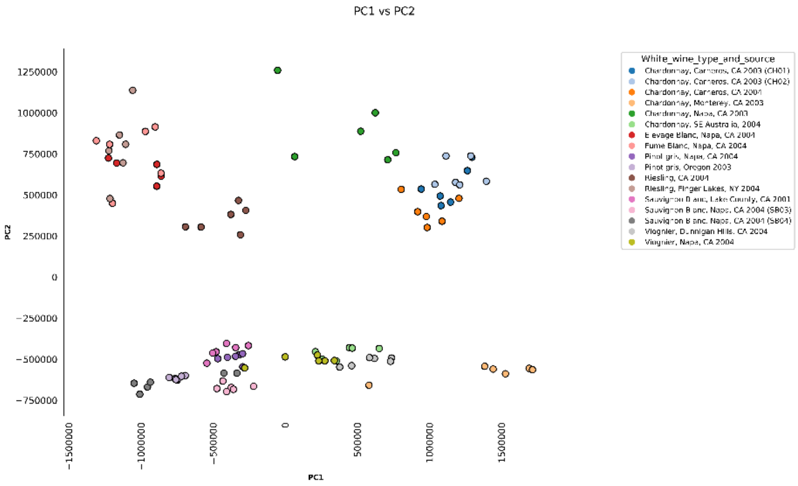

Scatter Plot 3D

The tool provides a 2D scatter plot of values in a Long Format file. If coloring by group is desired, the column with the sample names in the Long Format dataset has to have the name "sampleID" to match the name in the Design File. Scatter plot 2D allows the user to plot any pair of values from the Principal Component Analysis (PCA) output or plot other data.

NOTE: The user has to check that the Long Format and Design File datasets have no missing values.

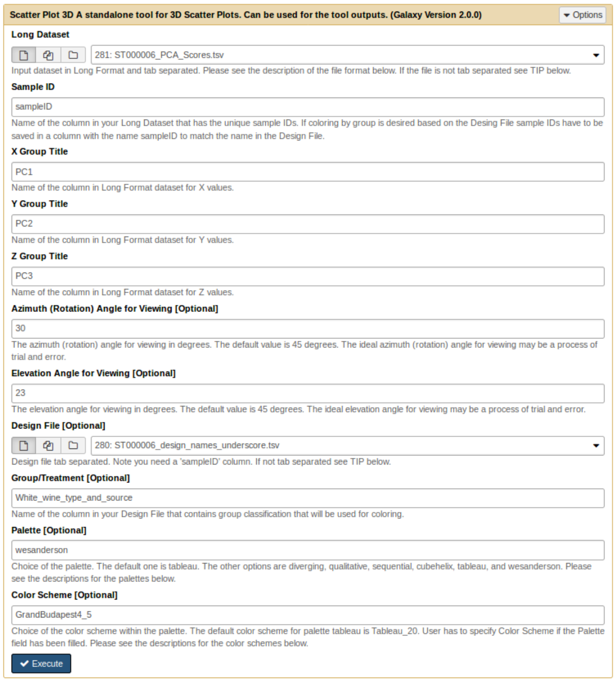

1. Select the Long Dataset from the drop-down menu. The scores in the PCA tool are output in the long format or the user can upload some other data set.
2. In the **Sample ID** text box, type the name of the **Sample ID** column from the **Long Format** dataset. In order for the coloring to work correctly this name should be the same as in the **Design File** i.e. the name for the samples column should be **sampleID.**
3. In the **X Group Title** text box, type the name the column name from your **Long Format** that you want to use for X axis.
4. In the **Y Group Title** text box, type the name the column name from your **Long Format** that you want to use for Y axis.
5. In the **Z Group Title** text box, type the name the column name from your **Long Format** that you want to use for Z axis.
6. Specify the **Azimuth (Rotation) Angle for Viewing [Optional]** in degrees. The default value is 45 degrees. The ideal azimuth (rotation) angle for viewing may be a process of trial and error.
7. Specify the **Elevation Angle for Viewing [Optional]** in degrees. The default value is 45 degrees. The ideal elevation angle for viewing may be a process of trial and error.
8. Select the **Design File [Optional]** from the drop-down menu. The Design File should have a **Group/Treatment** grouping variable column that will be used for sample's coloring.
10. In the **Group/Treatment [Optional]** text box, type the name of the column in the **Design File** that identifies the groups that will be used for coloring.
11. In the **Palette [Optional]** text box, specify the palette. The default one is tableau. The other options are diverging, qualitative, sequential, cubehelix, tableau, and wesanderson. Please see the descriptions for the palettes below.

12. In the **Color Scheme [Optional]** specify the color scheme within the **Palette**. The default color scheme for palette tableau is Tableau_20. User has to specify **Color Scheme** if the **Palette** field has been filled. Please see the descriptions for the color schemes below.
13. Click **Execute**.

The user has an option to specify the palette and the color scheme within the palette. If the palette is specified the color scheme has to be specified. The list of available palettes is diverging, qualitative, sequential, cubehelix, tableau, and wesanderson. The lists of corresponding color schemes that are available within each palette are available via the links below:

https://jiffyclub.github.io/palettable/tableau/
https://jiffyclub.github.io/palettable/colorbrewer/diverging/
https://jiffyclub.github.io/palettable/colorbrewer/qualitative/
https://jiffyclub.github.io/palettable/colorbrewer/sequential/
https://jiffyclub.github.io/palettable/cubehelix/
https://jiffyclub.github.io/palettable/wesanderson/

Output

This tool outputs one file:

- A PDF file containing a 3D scatterplot of the selected data.

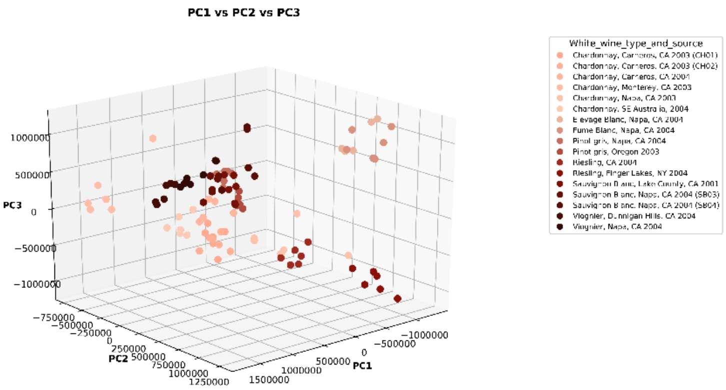

Standardized Euclidean Distance (SED)

The tool is designed to identify samples that are different using the standardized Euclidian distance (SED) between samples. The tool estimates the variance of features and calculates the SED between each pair of samples in addition to the SED between each sample and the estimated mean. If a group or treatment variable is provided, then the same distance plots are generated for each group and for all samples together.

SED computations are performed using the "DistanceMetric" function from the scikit-learn package in Python.

NOTE: Groups with less than three samples will be excluded from the analysis.

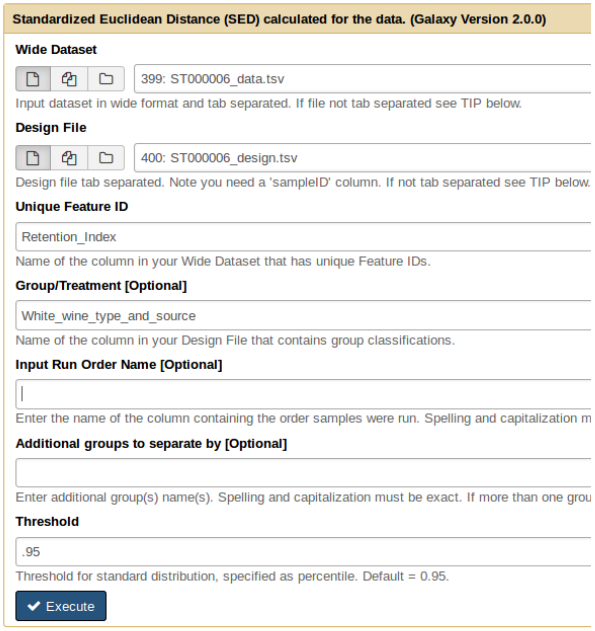

1. Select the **Wide Dataset** from the drop-down menu.
2. Select the **Design File** from the drop-down menu.
3. In the **Unique Feature ID** text box, type the name of the **Unique feature identifier**.
4. In the **Group/Treatment [Optional]** text box, type the name of the column in the **Design File** that identifies your groups.
5. In the **Input Run Order Name [Optional]** text box, type the name of the column in your **Design File** that has the Run Order variable.
6. In the **Additional groups to separate by [Optional]** text box, type the name of additional columns in your **Design File** to separate your data by.
7. In the **Threshold** text box, type the threshold, specified as a percentile, to be used for the distribution of the distance values. The default is 0.95.
8. Click **Execute**

Output

This tool outputs three different files:

- TSV file. This file contains the distances from each sample to the estimated mean.
    - **sampleID**
    - *SED_to_mean:* distance from the sample to the estimated mean. If **Group/Treatment [Optional]** is distance will be calculated from each sample to the estimated group mean.
- TSV file. This contains a n x n matrix (where n is the number computed samples) of the pairwise distances between the samples. If the **Group/Treatment [Optional]** variable is specified, the distances will be computed within the groups.
    - **sampleID**
- A PDF file containing
    - Boxplots of the distribution of distances.

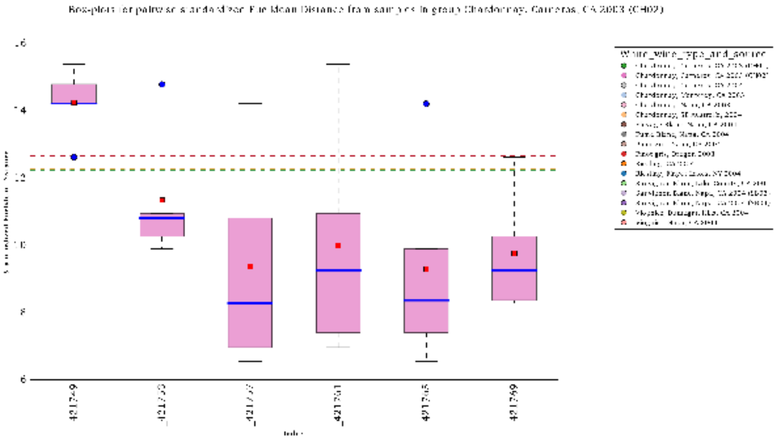

Euclidean distances are computed pairwise for each sample within a group. All pairwise distances computed are summarized for each sample as boxplots. Potential outliers (blue dots), means (red squares), and median (dark blue bars) are displayed. The threshold to declare a potential outlier is specified as threshold (specified as a percentile) in the input. This example shows the Standardized Euclidean Distances in the "Chardonnay, Carneros, CA 2003 (CH02)" group. The dashed lines correspond to the cutoffs are computed from beta, normal and chi-squared distributions in red, yellow and green respectively. Please note that normal and chi-squared cutoffs are expected to be very close to each other.

- 2D scatter plots that show the distances

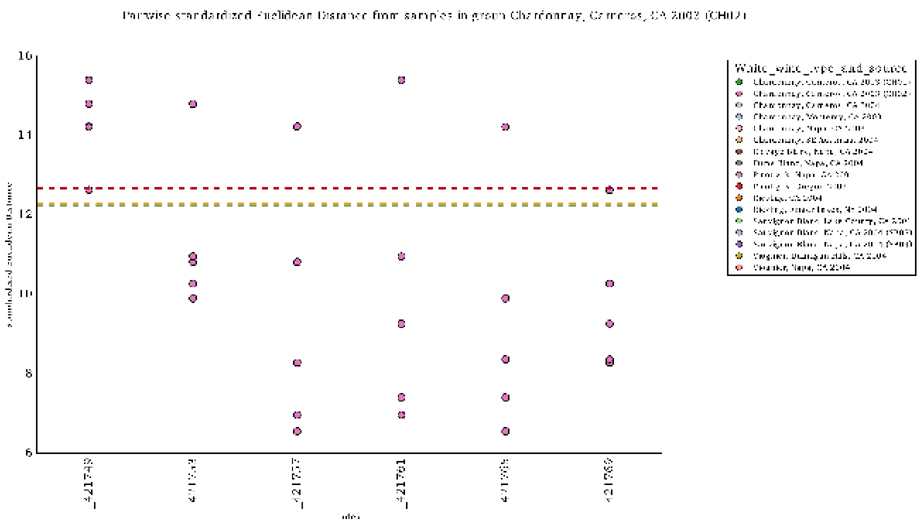

Figure showing a scatterplot of the Standardized Euclidean Distances in the “Chardonnay, Carneros, CA 2003 (CH02)” group. Distances are computed pairwise between samples within a group. The dashed lines correspond to the cutoffs are computed from beta, normal and chi-squared distributions in red, yellow and green respectively. Please note that normal and chi-squared cutoffs are expected to be very close to each other.

Summary of the Flags

This tool takes a wide format flags file and summarizes information about the flags. The flag summary includes, for each feature: (i) the sum of the flags, (ii) the mean of the flags, (iii) an indicator if at least one of the original flags had a value of 1, and (iv) a second indicator if all of the original flags had a value of 1.

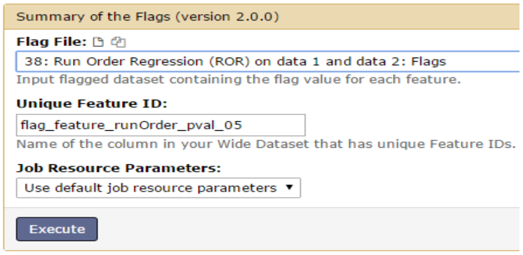

1. Select the **Wide Dataset** from the drop-down menu.
2. In the **Unique Feature ID** text box, type the name of the **Unique feature identifier**.
3. Click **Execute**.

Output

This tool will output one file:

- TSV Flag File. This file contains the same column names and values as the original **Flag File** with the addition of the following columns:
  - *flag_sum*: The sum of the value of the flags by feature (row). For example, if "row_1" flags are equal to "0","1", and "1" then flag_sum will be "2".
  - *flag_mean*: The mean of the flags by feature. For example, if "row_1" flags are equal to "0","1", and "1" then flag_mean will be "0.666".
  - *flag_any*: This flag will be a "1" if any of the flag values for the feature are a "1". For example, if "row_1" flags are equal to "0", "1", and "1" then flag_any will be "1".
  - *flag_all*: This flag will be a "1" only if all the flag values for the feature are a "1". For example, if "row_1" flags are equal to "0", "1", and "1" then flag_all will be "0".

Support Vector Machine (SVM) Classifier

Given a set of supervised samples in a **Training Dataset**, the SVM training algorithm builds a model based on these samples that can be used for predicting the categories of new, unclassified samples in a **Target Dataset**. The **Target Dataset** is not used for model training or evaluation, only for prediction based on the finalized model. SVM classification is performed on the target data and accuracy is estimated for both **Target** and **Training Datasets**.

SVM classification is performed using the "SVC", "GridSearchCV", and "cross_val_score" functions from the scikit-learn package in Python.

NOTE: Design files for both target and training datasets are required and the grouping variable column should be present in both files and have exactly the same name in both files. Unique Feature ID name should also be the same for both datasets and the feature names should be identical. Training Dataset can also be used as Target Dataset.

SVM uses different kernel functions to carry out different types of classification such as radial bassis (gaussian), linear, polynomial, and sigmoid. The classification model can be trained with and without cross-validation (single or double).

For no cross-validation the user specifies the value of the Regularization Parameter C. For cross-validation, the user specifies the Upper and Lower bounds for the Regularization Parameter C. For more information about the algorithm Regularization Parameter C see references below.

Details about the SVM algorithm can be found in the references below:

Cortes, C., & Vapnik, V. (1995). Support-vector networks. Machine learning, 20(3), 273-297.

Steinwart, I., & Christmann, A. (2008). Support vector machines. Springer Science & Business Media.

To use the SVM tool, users need to provide the following information:

(i) a **Training Dataset** with known categories in the training design file and

(ii) a **Target Dataset** with predicted categories in the target design file.

(iii) the name of the **Group/Treatment** classification column should be the same for both design files.

(iv) the U**nique Feature IDs** should be the same in both the wide datasets.

(v) the number of **Unique Feature ID**s should be the same in both the wide datasets.

NOTE: A minimum of 100 samples is required by the tool for single or double cross validation. The use of machine learning algorithms (including the support vector machine classifier) and cross-validation on datasets with small numbers of samples is controversial.

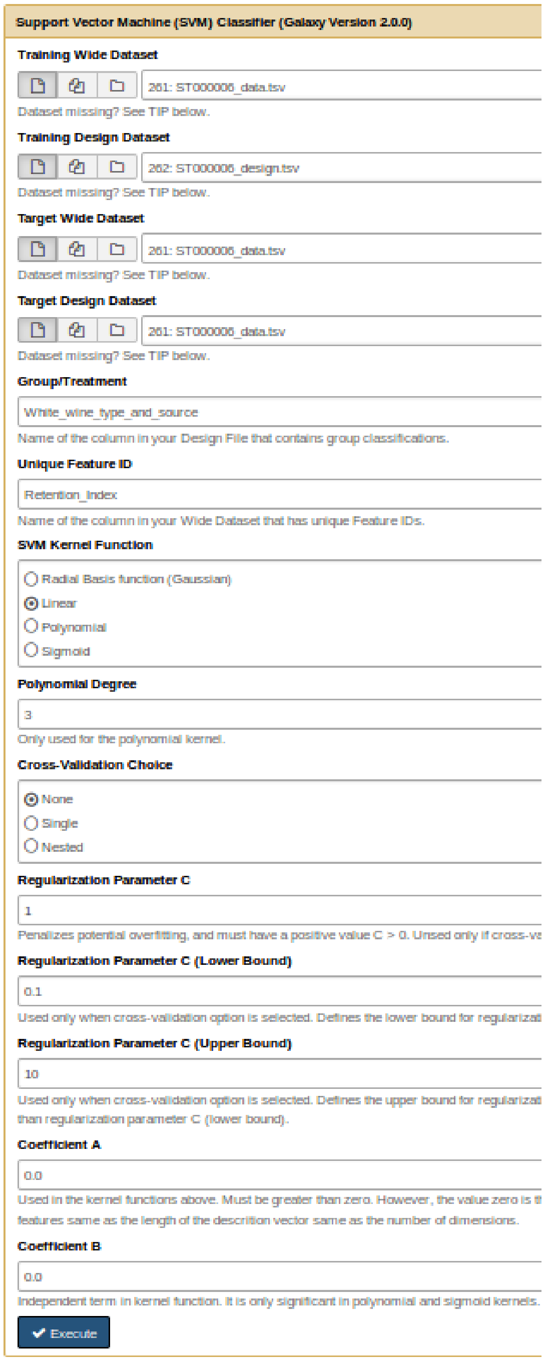

1. Select your ***training*** Wide dataset from the drop down menu.
2. Select your ***training*** **Design file** in the corresponding drop down menu.
3. Select your ***target*** **Wide dataset** from the corresponding drop down menu. Training wide dataset can also be used as target wide.
4. Select your ***target*** **Design file** in the corresponding drop down menu. Training design dataset can also be used as target design.
5. In the **Group/Treatment** text box, type the name of the column in the **Design File** that identifies your groups.
6. In the **Unique Feature ID** text box, type the name of the unique feature identifier.
7. Select the In the **Group/Treatment** text box, type the name of the column in the **Design File** that identifies your groups.
8. In the **Unique Feature ID** text box, type the name of the **Unique feature identifier**
Select the elect the **Polynomial Degree** used in the algorithm as an integer. The default degree is cubic (3). The parameter is used for *polynomial kernels only.*
9. Select the **SVM Kernel function**
10. Select the **Polynomial Degree** used in the algorithm as an integer. The default degree is cubic (3). The parameter is used for *polynomial kernels only.*
11. Select **Cross-validation Options** parameter. None corresponds to no cross-validation. Nested corresponds to nested cross-validation which is also called double.
12. Select **Regularization Parameter C.** This field is relevant only if Cross-validation Options field is none. Larger values for C add to the bias and overfitting but decrease misclassification. Smaller values for C decrease bias and overfitting but increase

misclassification. The default value is 1 and must be positive. The parameter is *used for all kernels.*

13. Select **Regularization Parameter C (Lower Bound)**. This field is relevant only if Cross-validation Options parameter is either single or nested. Has to be positive.
14. Select **Regularization Parameter C (Upper Bound)**. This field is relevant only if Cross-validation Options parameter is either single or nested. Has to be positive and bigger than **Regularization Parameter C (Lower Bound)**.
15. Select **Coefficient A** the kernel coefficient gamma parameter. The parameter must be greater than zero. If zero value is specified it will be converted to 1/(number of features). This is the default value. The parameter is only relevant *for radial basis function, polynomial and sigmoid kernels*.
16. Select **Coefficient B** the independent term in the kernel function. The default value is zero. The parameter is only relevant for *polynomial and sigmoid kernels.*
17. Click **Execute**.

NOTE: The parameters in fields 7, 8, 10, 11, 12, 13, 14 require detailed knowledge of SVM

SVM is implemented with scikit module svm-kernels Nested cross validation is implemented with scikit module nested_cross_validation

Details about the cross-validation method(s) are available via Cawley, Gavin C., and Nicola LC Talbot. "On over-fitting in model selection and subsequent selection bias in performance evaluation." Journal of Machine Learning Research 11, no. Jul (2010): 2079-2107.

Output

This tool will output four files:

- TSV file. This file contains the classification produced by the model for each sample in the training data set.
    - ***SampleID***
    - ***Group_Observed:*** Initial group labels.
    - ***Group_Predicted:*** Predicted group labels.
- TSV file. This file contains the classification accuracy in percent between the classes in the training data set.
- TSV file. This file contains the classification produced by the model for each sample in the target data set.
    - ***SampleID***
    - ***Group_Observed:*** Initial group labels.
    - ***Group_Predicted:*** Predicted group labels.

- TSV file. This file contains the classification accuracy in percent between the classes in the target data set.

Threshold Based Flags

This tool flags a feature in a given group with a binary indicator if, for half (or more) of the samples within the group, the feature value is below a user specified threshold or is missing. The default threshold value of 30,000 is primarily useful for peak intensities from mass spectroscopy data and should be evaluated carefully for other types of values (e.g. for peak height).

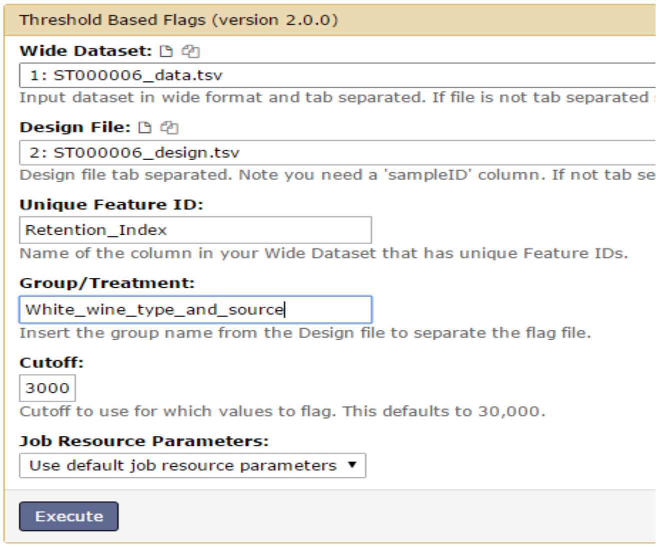

1. Select the **Wide Dataset** from the drop-down menu.
2. Select the **Design File** from the drop-down menu.
3. In the **Unique Feature ID** text box, type the name of the **Unique feature identifier**.
4. In the **Group/Treatment** text box, type the name of the column in the **Design File** that identifies your groups.
5. In the **Cutoff** text box, type a threshold value. The default value is 30,000.
6. Click **Execute**.

Output

This tool outputs one file:

- TSV file: The file contains flags for each feature by group. The file contains the following columns:
    - ***Unique Feature ID***
    - *flag_feature{group}_off* is flagged a “1” if the feature is below the **Cutoff** value in half of more of the samples in {group}.
    - *flag_feature_any_off* is flagged a “1” when at least one of the *flag_feature{group}_off* for that feature is flagged a “1”.
    - *flag_feature_all_off* is flagged a “1” when all the *flag_feature_{group}_off* for that feature are flagged a “1”.

T-Test (Single Group)

The tool performs a one sample t-test for each feature. Two options are available for the t-test: if the user provides the **Group/Treatment** variable then the mean for each treatment condition is compared with **Mu**, the user-specified value of the true mean under the null hypothesis. If **Group/Treatment** is not provided, then the sample mean is compared to **Mu**.

T-Test (Single Group) is performed using the "ttest_1samp" function from the SciPy package in Python.

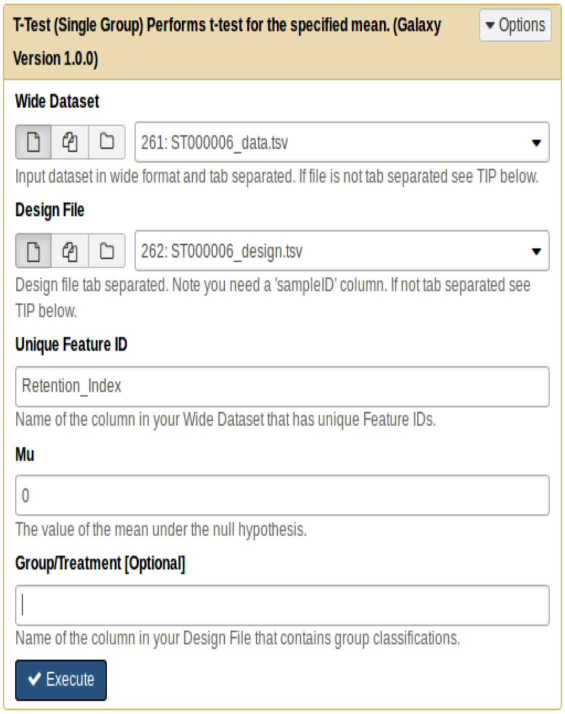

1. Select the **Wide Dataset** from the drop down menu.
2. Select the **Design File** from the drop-down menu.
3. In the **Unique Feature ID** field type the name of the unique Feature identifier.
4. Specify the value of **Mu**. The default value is 0.
5. Specify the **Group/Treatment [Optional]** field. This field should contain the grouping variable (column name) in the **Design File** that identify the grouping variable used for the test. This filed is optional.
6. Click **Execute.**

Output

This tool outputs two TSV files: one file with summary statistics and another file with flags. If no Group/Treatment variable is provided group i is the only group and is called "all" in the summary statistics file. If Group/Treatment variable is provided then the group i variable below is replaced by the group name.

- TSV file for the summary table with the columns:
  - *Unique Feature ID*
  - *GrandMean*: Mean across all samples
  - *SampleVariance*: Variance across all samples

- *Mean_{group_i}*: mean for samples in group i
- *diff_of_{group_i_Mu}*: difference in the mean for samples in group i compared to the value of Mu.
- *t-value_for_diff_{group_i_Mu}*
- *prob_greater_than_t_for_diff_{group_i_Mu}*
- *neg_log10_p_value_{group_i_Mu}*

- TSV file for the flags table with the columns:
  - *flag_significant_0p05_on_{group_i_Mu}* is equal to 1 if prob_greater_than_t_for_diff_{group_i_Mu} is less than 0.05
  - *flag_significant_0p01_on_{group_i_Mu}* is equal to 1 if prob_greater_than_t_for_diff_{group_i_Mu} is less than 0.01
  - *flag_significant_0p1_on_{group_i_Mu}* isequal to if prob_greater_than_t_for_diff_{group_i_Mu} is less than 0.1

- PDF file with the volcano plot(s) for the difference(s).

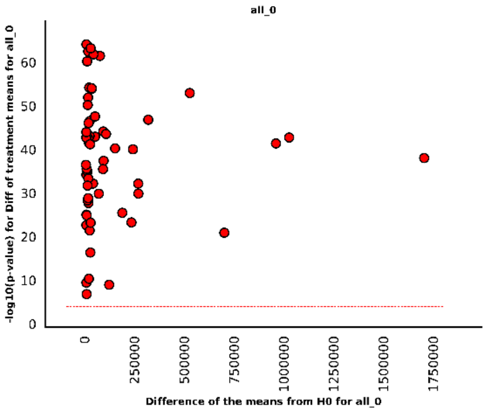

The volcano plot is produced based on the values of the differences between the mean of a current group i and the value of μ for each feature and the corresponding negative log (base 10) p-values. The differences are displayed on x-axis and the re-scaled p-values are displayed on the y-axis. Each dot on the plot represents a feature. Individual volcano plots are generated for each group, if **Group/Treatment** is given, and single volcano plot is generated if no grouping variable is provided. The red dashed line in the volcano plot(s) corresponds to a p-value = 0.01 (2 on the negative log base 10 scale). Volcano plots were first described in:

Jin, Wei, Rebecca M. Riley, Russell D. Wolfinger, Kevin P. White, Gisele Passador-Gurgel, and Greg Gibson. "The contributions of sex, genotype and age to transcriptional variance in Drosophila melanogaster." Nature genetics 29, no. 4 (2001): 389-395.

T-Test (Unpaired or Paired)

The tool performs a two-sided t-test for two groups of dependent samples (paired or dependent t-test) or multiple (two or more) groups of independent samples (unpaired or independent t-test). The user selects which test (paired or unpaired) to perform.

In an unpaired t-test the samples within and between groups are independent. The test is performed for all pairs using the **Group/Treatment** field. For example, if there are three treatment conditions (Control, Time1 and Time2) then t-tests will be performed for: (i) Control vs Time1, (ii) Control vs Time2, and (iii) Time1 vs Time2. Note that this will give slightly different results than the contrast in an ANOVA because the ANOVA uses all groups to estimate the error.

A paired t-test can be performed for pairs of treatment conditions where sample pairs are known and specified by the user in the **Pairing ID** field. Here, the difference between the measurements for the pairs is calculated. To ensure that the differences are taken in the same order across all pairs, the **Group/Treatment** variable is required. The differences will be calculated between the groups in the order that the groups appear in the **Design File**. The **Pairing ID** specifies which samples are paired. A two sided t-test will be performed for the test of the null hypothesis that the difference is zero.

T-Test (Unpaired or Paired) are performed using the “ttest_ind” and “ttest_rel” functions from the SciPy package in Python.

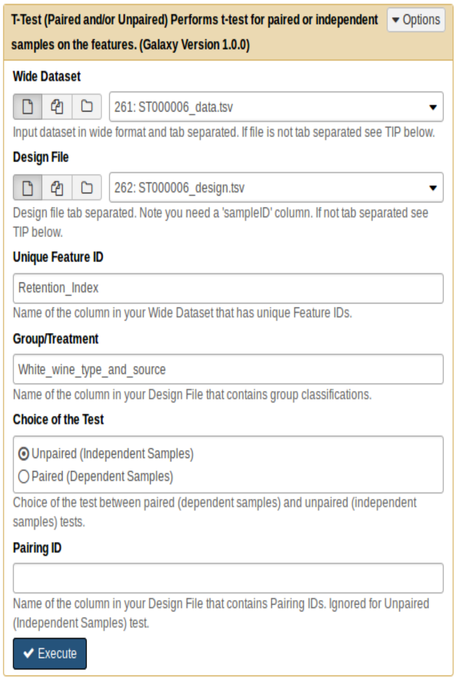

1. Select the **Wide Dataset** from the drop down menu.
2. Select the **Design File** from the drop-down menu.
3. In the **Unique Feature ID** field type the name of the unique Feature identifier.
4. Specify the **Group/Treatment** field. This field should contain the grouping variable (column name) in the **Design File** that identify the grouping variable used for the test.
5. Select the desired test in the **Select Test** field. There are two options: Unpaired (Independent Samples) and Paired (Dependent Samples).
6. Specify the **Pairing ID**. This variable is required for the Paired (Dependent Samples) test to run. This variable is not applicable when the Unpaired (Independent Samples) test is selected.
7. Click **Execute.**

Output

This tool outputs two TSV files: one file with summary statistics and another file with the flags. The comparisons are performed for pairs of different groups i and j provided by the user in **Group/Treatment** variable. Only two group are compared in paired t-test due to the test design. The number of comparisons in unpaired t-test is not limited and is controlled by the number of groups provided by the user in **Group/Treatment** variable.

- TSV file for the summary table with the columns:
  - *Unique Feature ID*
  - *GrandMean*: Mean across all samples
  - *SampleVariance*: Variance across all samples
  - *mean {group_i}*: mean for samples in group i
  - *mean {group_j}*: mean for samples in group j
  - *diff_of_{group_i_ group_j}*: difference in the mean for samples in group i compared to group j.
  - *t-value_for_diff_{group_i_ group_j}*
  - *prob_greater_than_t_for_diff_{group_i_ group_j}*
  - *neg_log10_p_value_{group_i_ group_j}*

- TSV file for the flags table with the columns:
  - *flag_significant_0p05_on_{group_i_ group_j}* is equal to 1 if prob_greater_than_t_for_diff_{group_i_ group_j} is less than 0.05
  - *flag_significant_0p01_on_{group_i_ group_j}* is equal to 1 if prob_greater_than_t_for_diff_{group_i_ group_j} is less than 0.01
  - *flag_significant_0p1_on_{group_i_ group_j}* is equal to if prob_greater_than_t_for_diff_{group_i_ group_j} is less than 0.1

- PDF file with the volcano plot(s) for the difference(s).

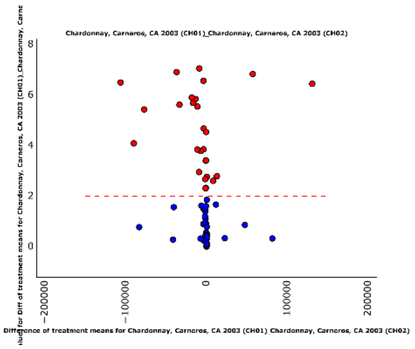

The volcano plot is produced based on the values of the differences between the means of group i and group j for each feature and the corresponding negative log (base 10) p-values. The differences

between the group values are displayed on x-axis and the re-scaled p-values are displayed on the y-axis. Each dot on the plot represents a feature. Individual volcano plots are generated for each pair of groups compared by the t-test. The red dashed line in the volcano plot(s) corresponds to a p-value = 0.01 (2 on the negative log base 10 scale). Volcano plots were first described in:

Jin, Wei, Rebecca M. Riley, Russell D. Wolfinger, Kevin P. White, Gisele Passador-Gurgel, and Greg Gibson. "The contributions of sex, genotype and age to transcriptional variance in Drosophila melanogaster." Nature genetics 29, no. 4 (2001): 389-395.

Group Comparison by Permutation

The tool performs a permuted two-sided pairwise comparison of multiple (two or more) groups of independent samples where the p-value is calculated by permutation of the data (http://www.jarrodmillman.com/publications/millman2015thesis.pdf, https://github.com/statlab/permute).

The permutation test is carried out on all pairs of conditions specified using the **Group/Treatment** field. The number of iterations is specified using the **Iteration Number** field. If there are three treatment conditions (Control, Time1 and Time2) then a t statistics will be calculated for: (i) Control vs Time1, (ii) Control vs Time2, and (iii) Time1 vs Time2. The following parameters in the original are code are set to the following values: (1) stat = 't' for a two-sample t-statistic, (2) alternative = 'two-sided' as the alternative hypothesis, and (3) seed = 'None' to use the pseudorandom number generator.

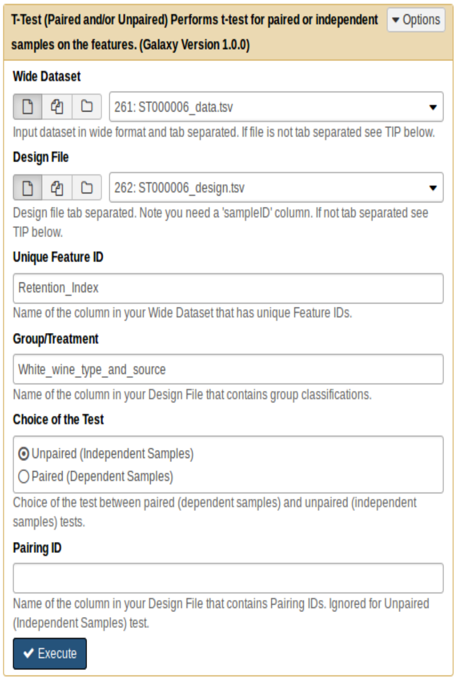

1. Select the **Wide Dataset** from the drop down menu.
2. Select the **Design File** from the drop-down menu.
3. In the **Unique Feature ID** field type the name of the unique Feature identifier.
4. Specify the **Group/Treatment** field. This field should contain the grouping variable (column name) in the **Design File** that identify the grouping variable used for the test.
5. Enter the number of iterations to carry out.
6. Click **Execute.**

Output

This tool outputs two TSV files: one file with summary statistics and another file with flags. Two groups are compared at a time. When the **Group/Treatment** variable contains more than two groups, all groups are compared pairwise.

TSV file for the summary statistics with the columns:

- *Unique Feature ID*
- *GrandMean*: Mean across all samples
- *SampleVariance*: Variance across all samples
- *mean {group_i}*: mean for samples in group i

- *mean {group_j}*: mean for samples in group j
- *diff_of_{group_i_ group_j}*: difference in the mean for samples in group i compared to group j.
- *t-value_for_diff_{group_i_ group_j}*
- *perm_greater_than_t_for_diff_{group_i_ group_j}*
- *neg_log10_p_value_{group_i_ group_j}*

- TSV file for the flags table with the columns:
  - *flag_significant_0p05_on_{group_i_ group_j} i*s equal to 1 if perm_greater_than_t_for_diff_{group_i_ group_j} is less than 0.05
  - *flag_significant_0p01_on_{group_i_ group_j}* is equal to 1 if perm_greater_than_t_for_diff_{group_i_ group_j} is less than 0.01
  - *flag_significant_0p1_on_{group_i_ group_j}* is equal to if perm_greater_than_t_for_diff_{group_i_ group_j} is less than 0.1

- PDF file with the volcano plot(s) for the difference(s).

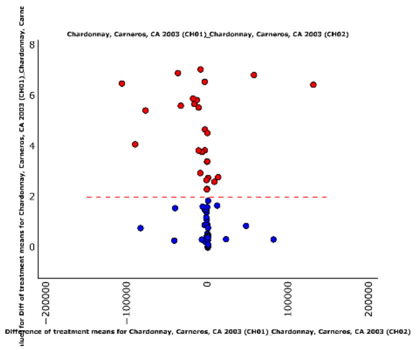

The volcano plot is produced based on the values of the differences between the means of group i and group j for each feature and the corresponding negative log (base 10) p-values estimated from permutation. The differences between the group values are displayed on x-axis and the re-scaled p-values are displayed on the y-axis. Each dot on the plot represents a feature. Individual volcano plots are generated for each pair of groups compared. The red dashed line in the volcano plot(s) corresponds to a p-value = 0.01 (2 on the negative log base 10 scale). Volcano plots were first described in:

Jin, Wei, Rebecca M. Riley, Russell D. Wolfinger, Kevin P. White, Gisele Passador-Gurgel, and Greg Gibson. "The contributions of sex, genotype and age to transcriptional variance in Drosophila melanogaster." Nature genetics 29, no. 4 (2001): 389-395.
