# Supplementary material for: SECIMTools: a suite of metabolomics data analysis tools
Source: BMC Bioinformatics. 2018 Apr 20;19:151. doi: 10.1186/s12859-018-2134-1 (PMC5910624; doi:10.1186/s12859-018-2134-1)
Supplement: Supplementary file 2 — Example input and output. (DOCX 3818 kb) [file 12859_2018_2134_MOESM2_ESM.docx]

**Supplemental Material**

**Retention Time (RT) Flags**

**Input Files:**

- Data file: TEST0000_rt.tsv
- Design file: TEST0000_design.tsv

**Tool options:**


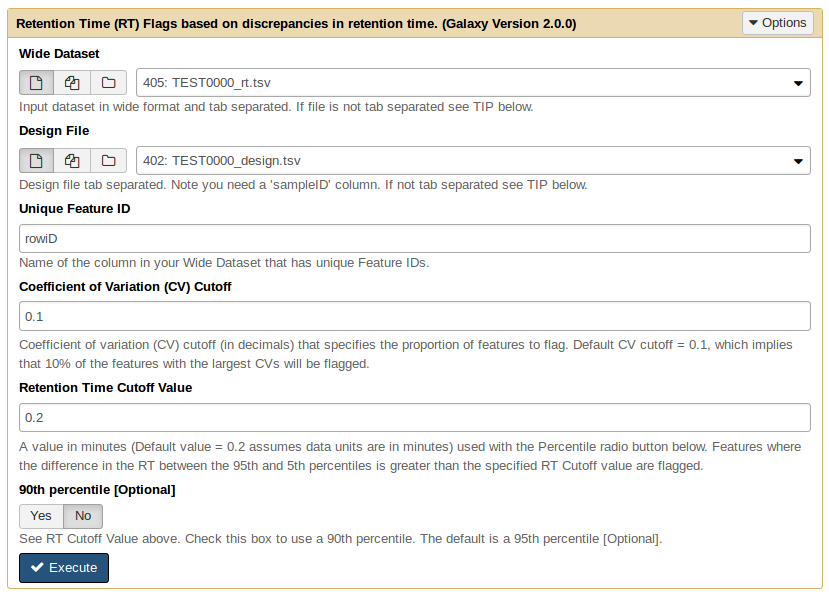


**Output:**


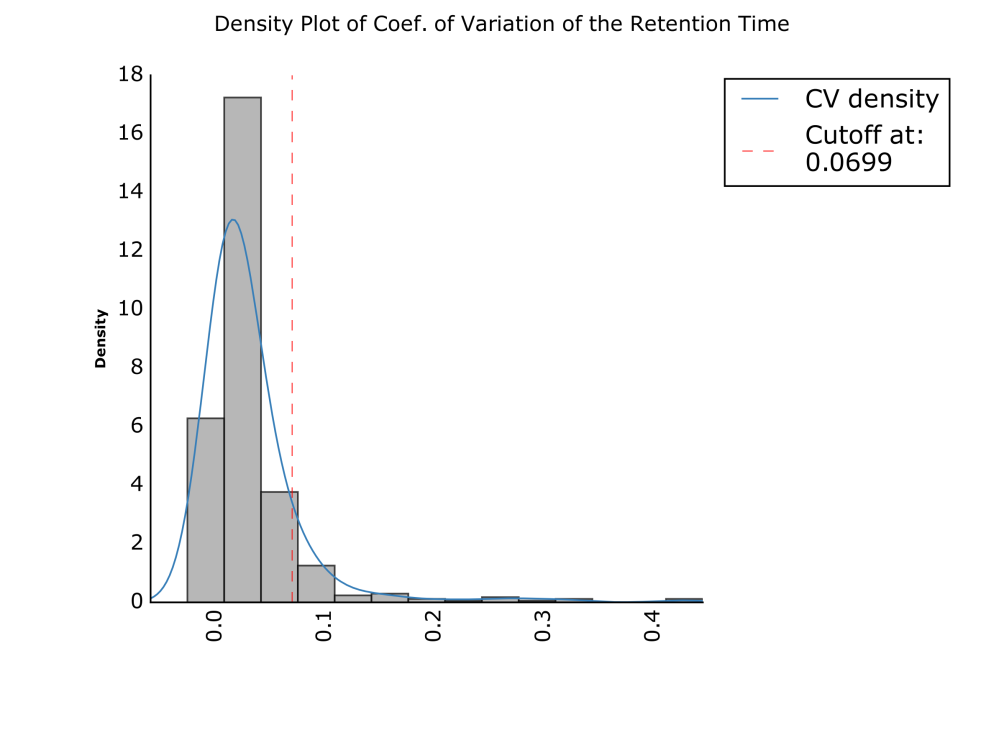


SF1. The histogram and smoothed density plot of the coefficients of variation for retention time. The red dotted line shows the cutoff for the top 10 % of the data.

**Run Order Regression (ROR)**

**Input Files:**

- Data file: ST000006_data.tsv
- Design file: ST000006_design.tsv

**Tool options:**


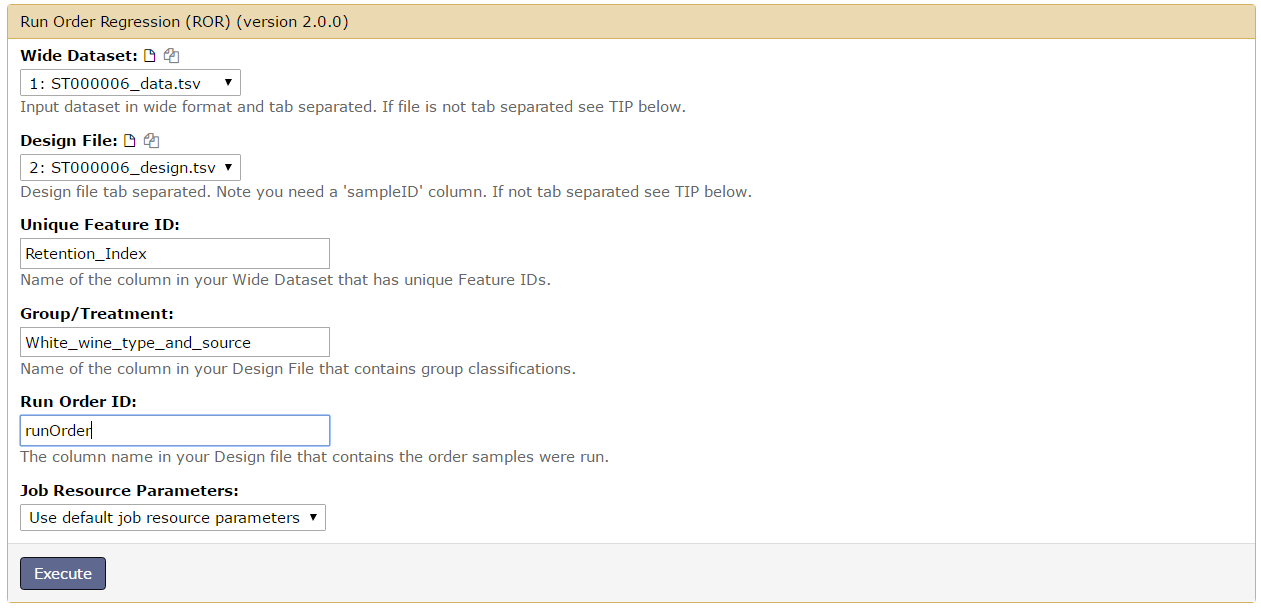


**Output:**


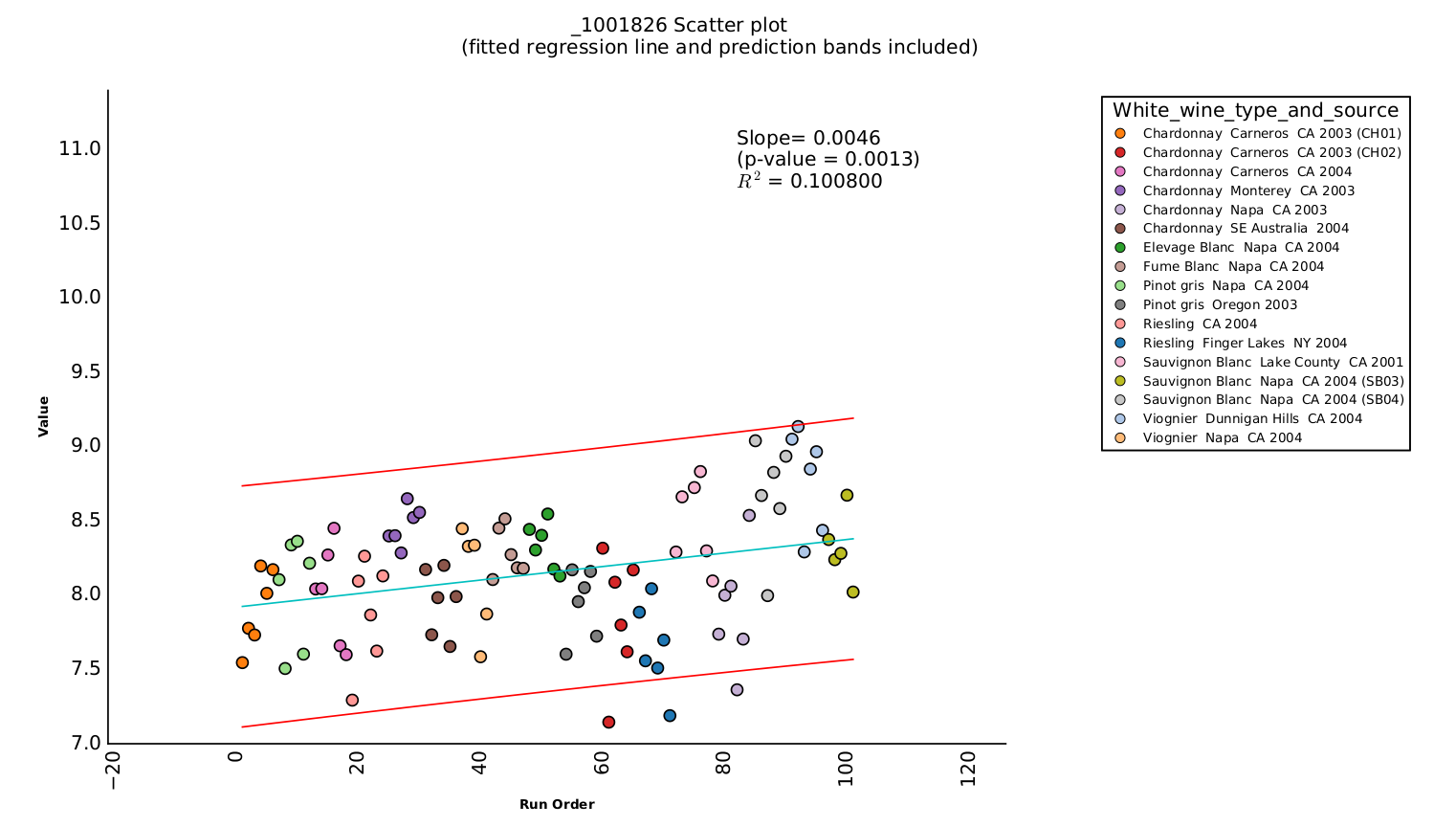


SF2. This plot shows the color-coded scatterplot of the values of “_1001826” feature for all samples. The run order of the samples is displayed along the $x$-axis and the feature value is displayed on the $y$-axis. Each color represents a different group of samples. A regression line has been fit and the R^2^ Is displayed on the plot. If there is no effect of run order, the slope of the regression line will be zero. A test of the null hypothesis that the slope is zero is performed and the resulting $p$-value. The fitted line (light blue) and the 95% confidence bands (red) are displayed on the graph.

**Bland Altman (BA) Plot**

**Input Files:**

- Data file: ST000006_data.tsv
- Design file: ST000006_design.tsv

**Tool options:**


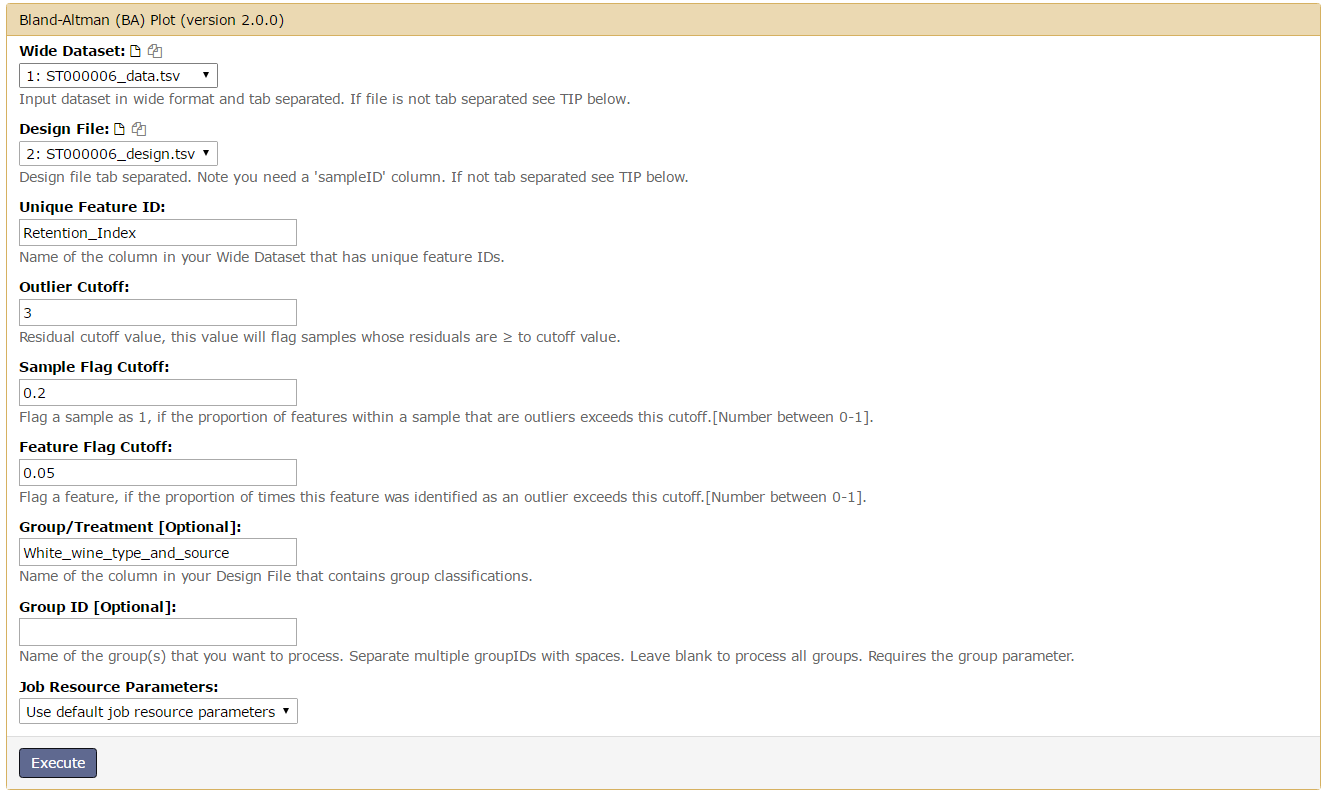


**Output:**


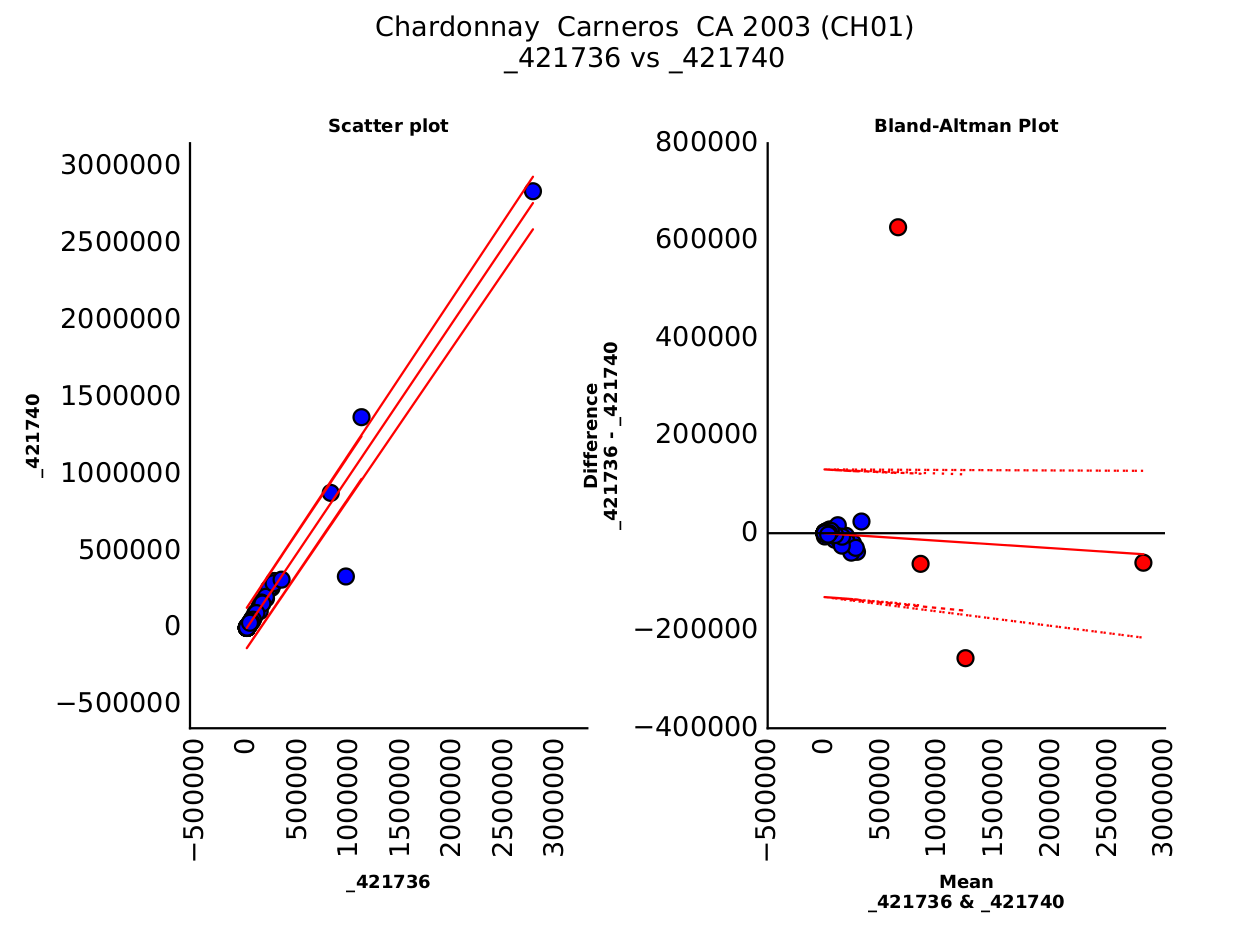


SF3.The Scatter plot (left) displays the values of the first sample (_421736) on the $x$-axis and the values of the second sample (_421740) on the $y$-axis. Samples representing replicates are expected to have similar feature values. The Bland-Altman plot (right) displays the mean of each feature for the two samples on the $x$-axis and the corresponding difference between features on the $y$-axis. A linear regression fit and the corresponding confidence bands are displayed on each graph in red. The slope of the line is expected to be zero. The Red dots represent the outliers that have been identified (flagged) using at least one of these methods: Cook’s Distance, Pearson Residuals and DFFITS.


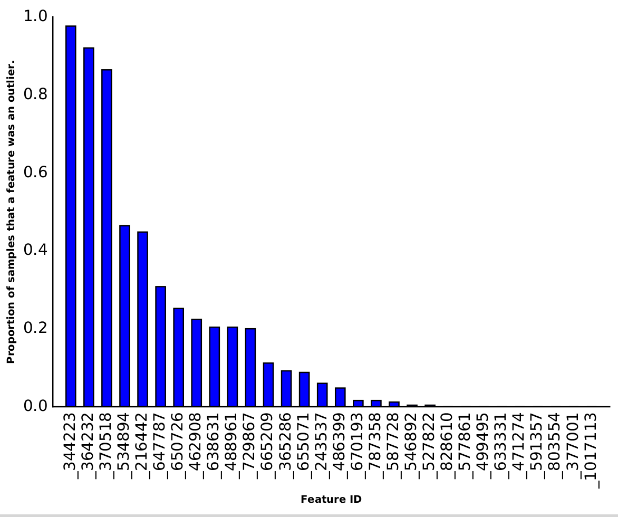

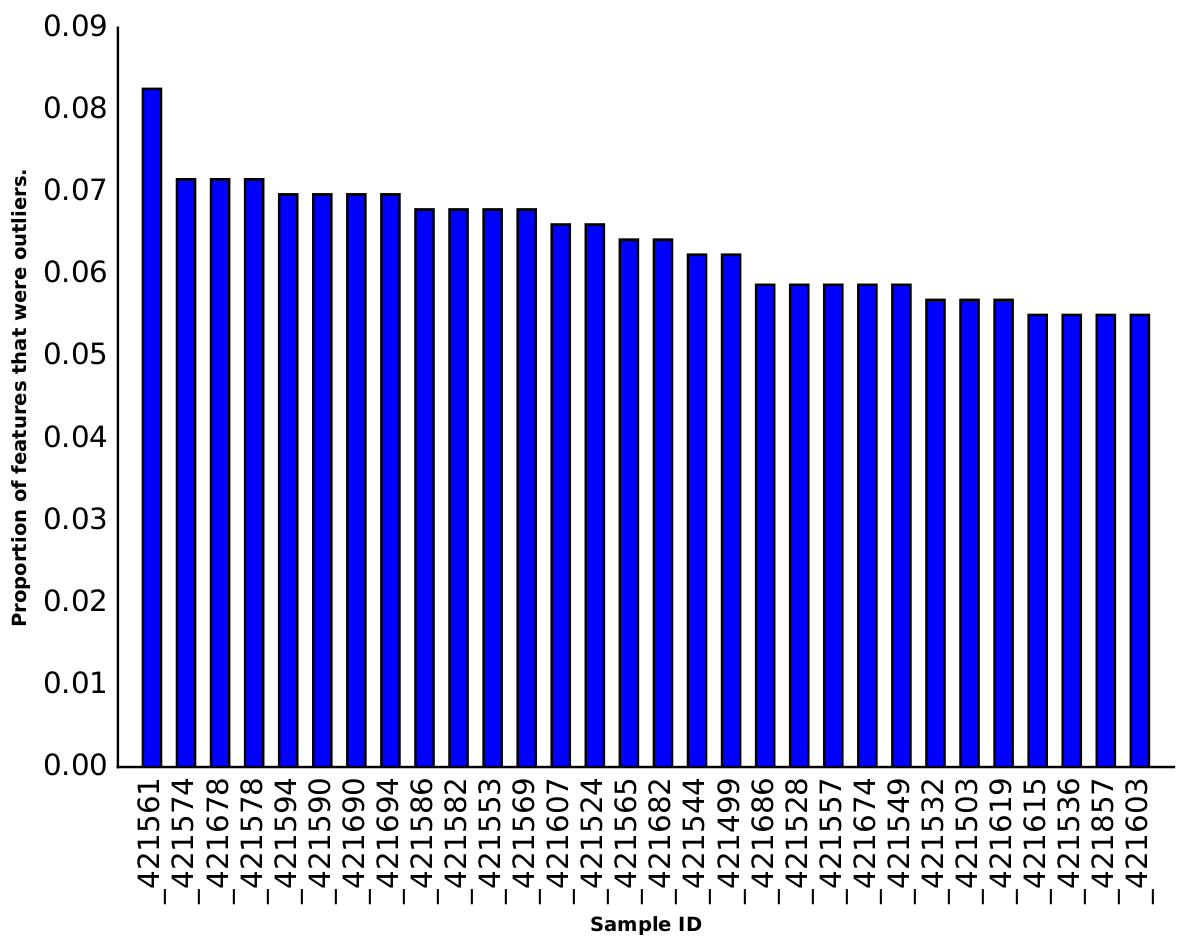


SF4. Histogram of 30 most features (left) and samples (right) with the largest number of flags produced by the BA Plot Tool.

**Coefficient of Variation (CV) Flags**

**Input Files:**

- Data file: ST000006_data.tsv
- Design file: ST000006_design.tsv

**Tool options:**


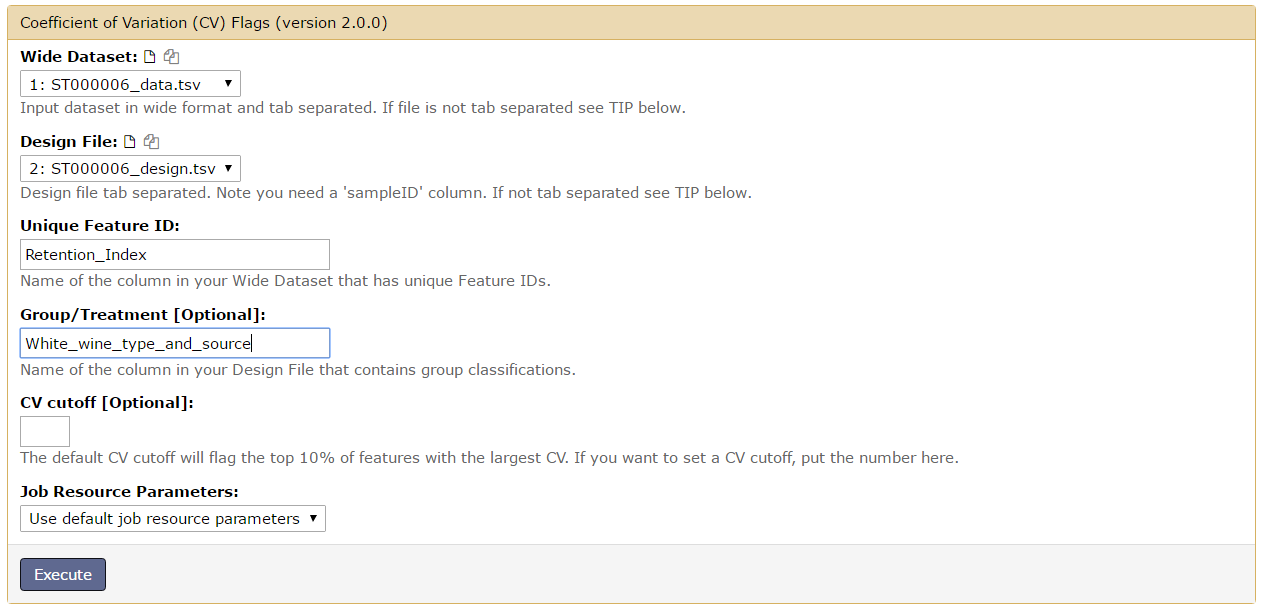


**Output:**


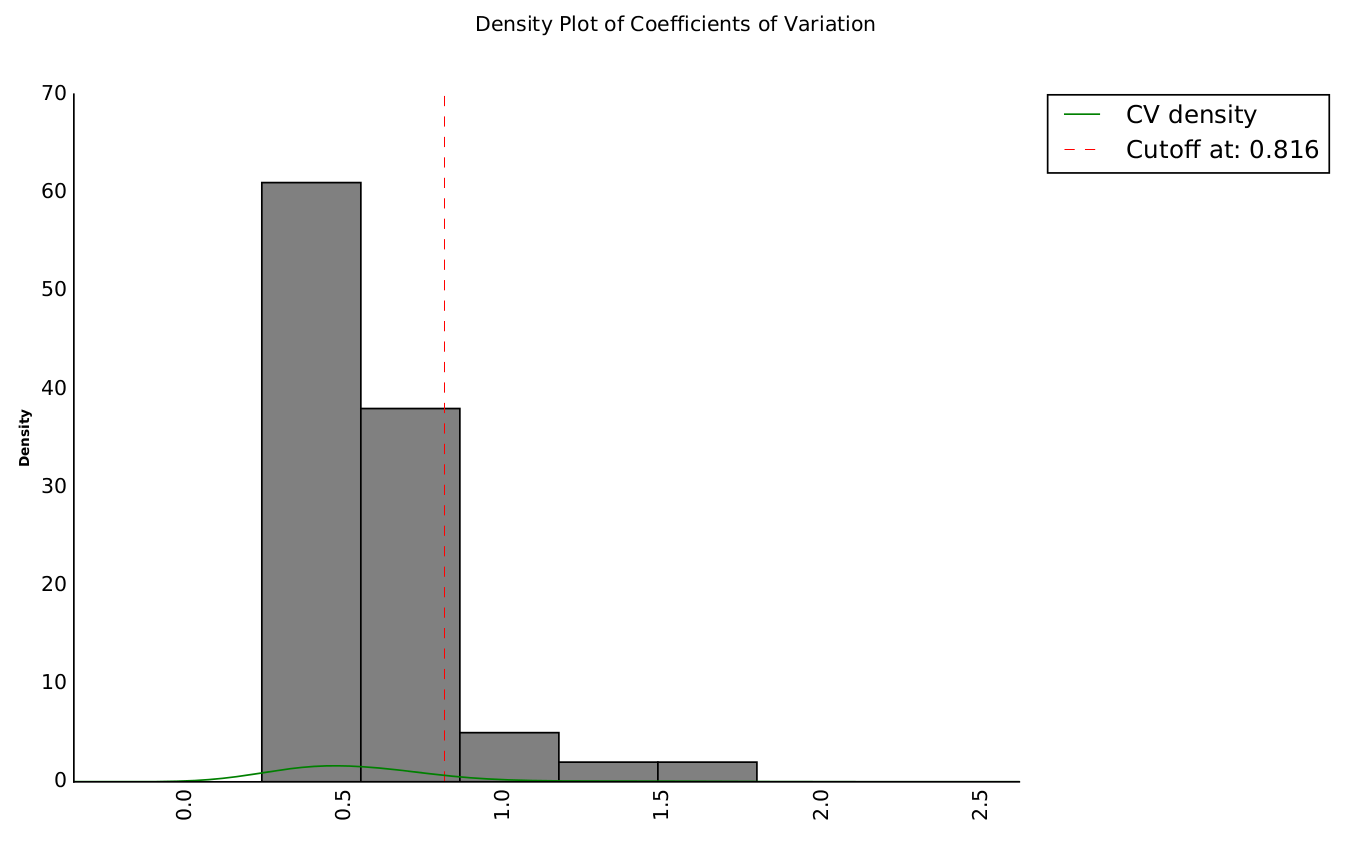


SF5. Histogram and corresponding density plot (in green) of the coefficients of variation for all samples. The dotted red line is the user adjustable threshold for large values of the CV.

**Magnitude Difference Flags**

**Input Files:**

- Data file: ST000006_data.tsv
- Design file: ST000006_design.tsv

**Tool options:**


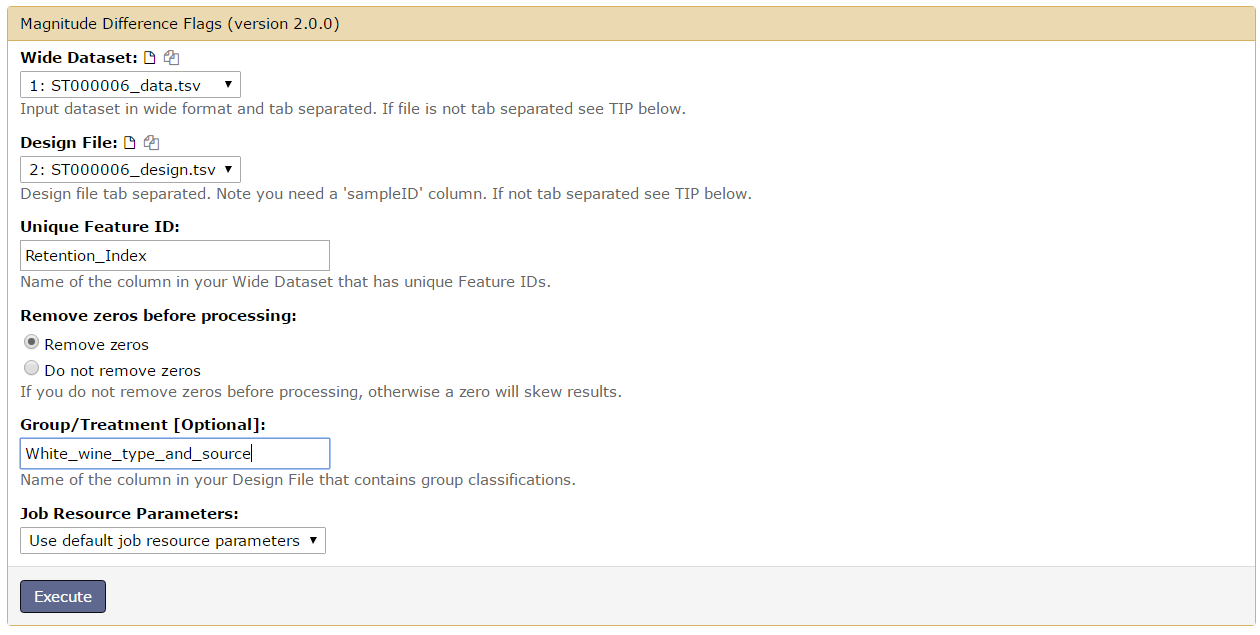


**Output:**


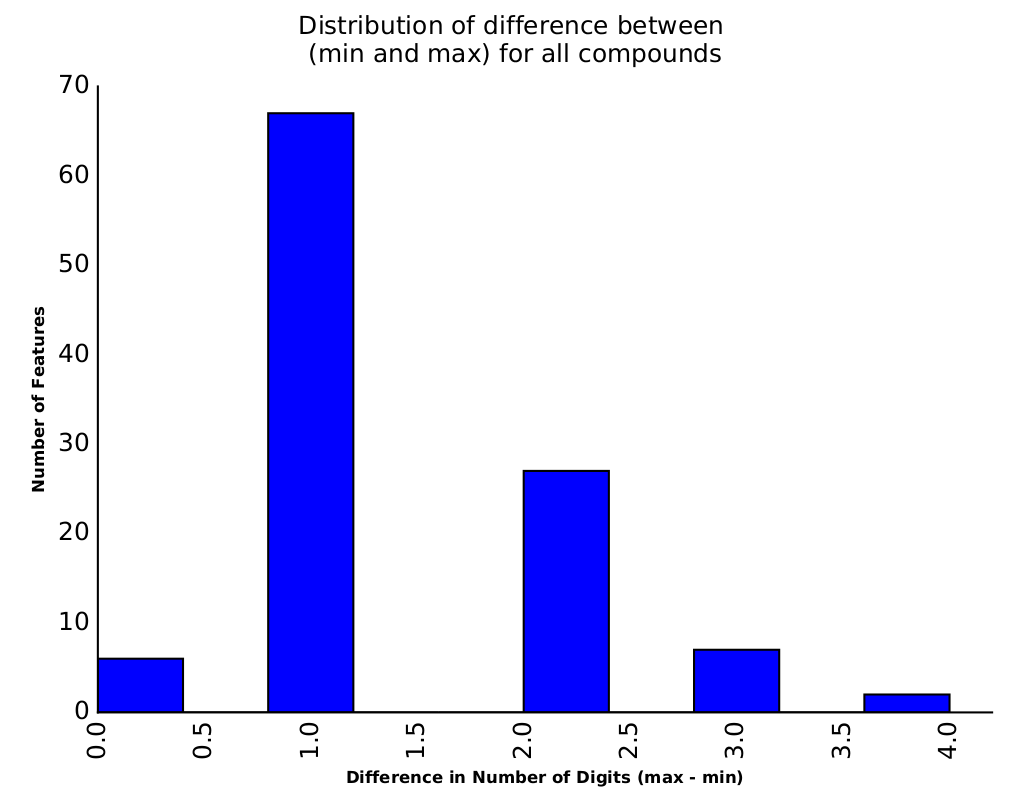


SF6: Histogram of the maximum differences between the largest and the smallest number of digits for a given feature across all samples. A difference of one means that there is an order of magnitude difference between the maximum and minimum value of feature intensity. Differences are performed within each treatment group.

**Distribution of Features across Samples**

**Input Files:**

- Data file: ST000006_data_log.tsv
- Design file: ST000006_design.tsv

**Tool options:**


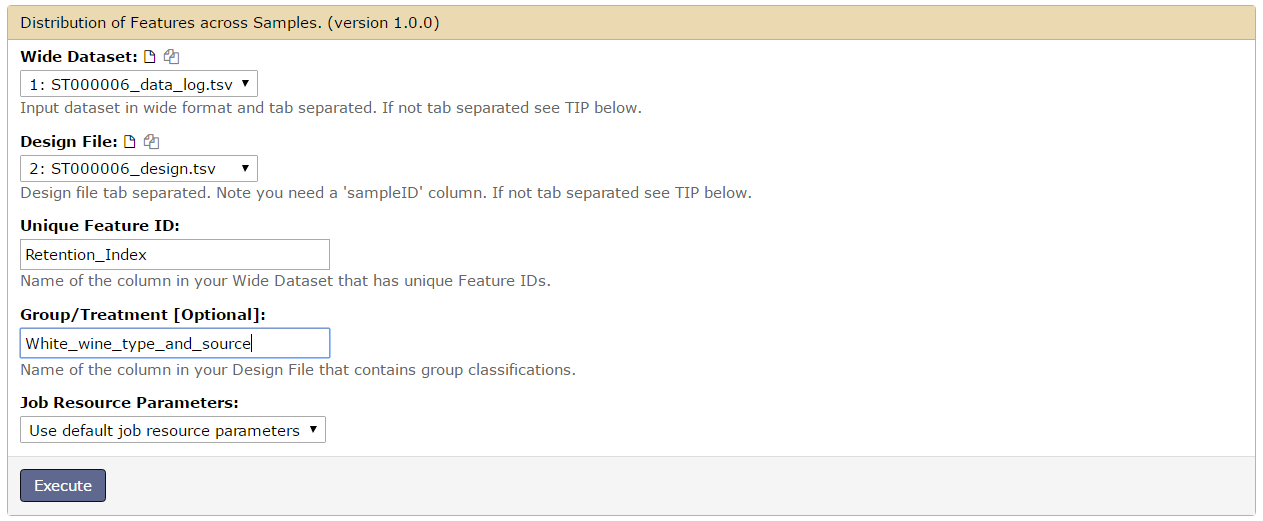


**Output:**


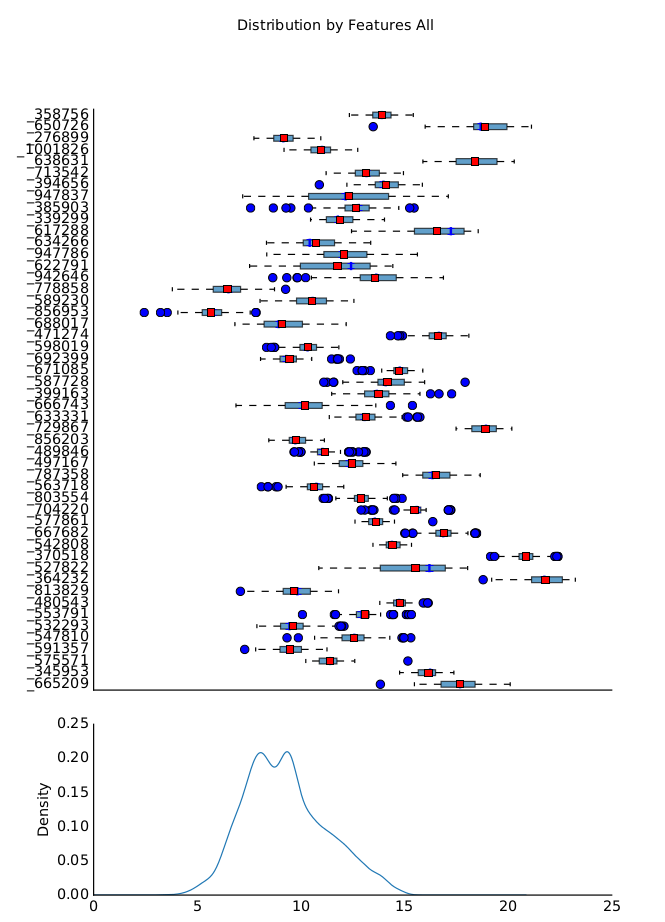


S7. Boxplots for 50 randomly selected features (top). Density plot for all features summarized on one graph (bottom).

**Distribution of Features within Samples**

**Input Files:**

- Data file: ST000006_data_log.tsv
- Design file: ST000006_design.tsv

**Tool options:**


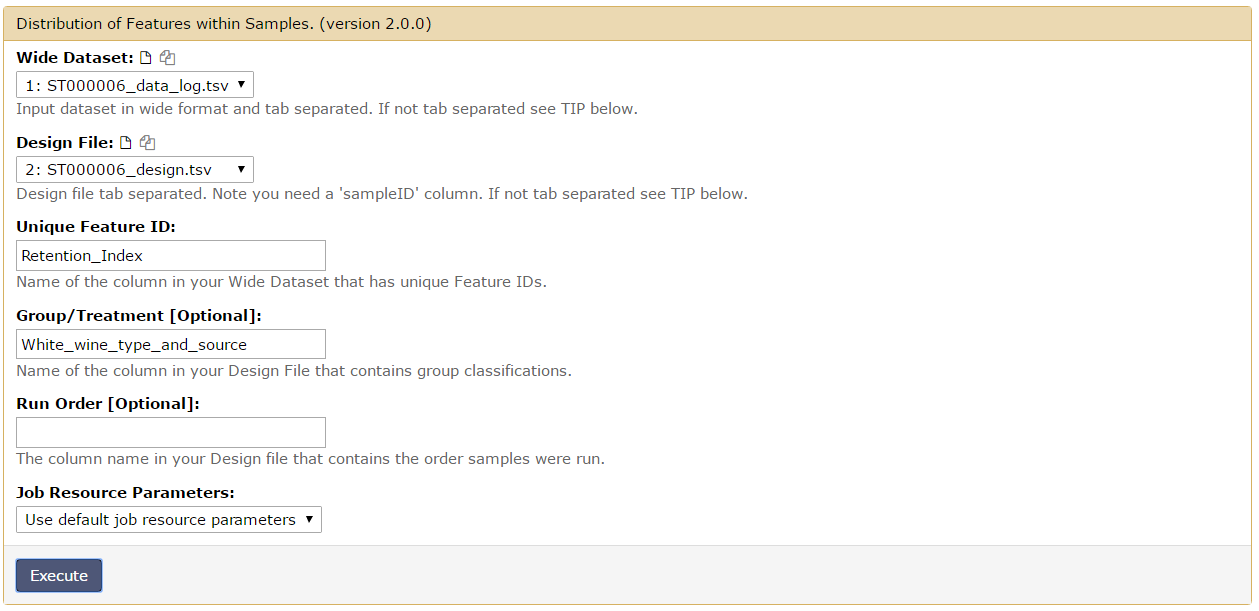


**Output:**


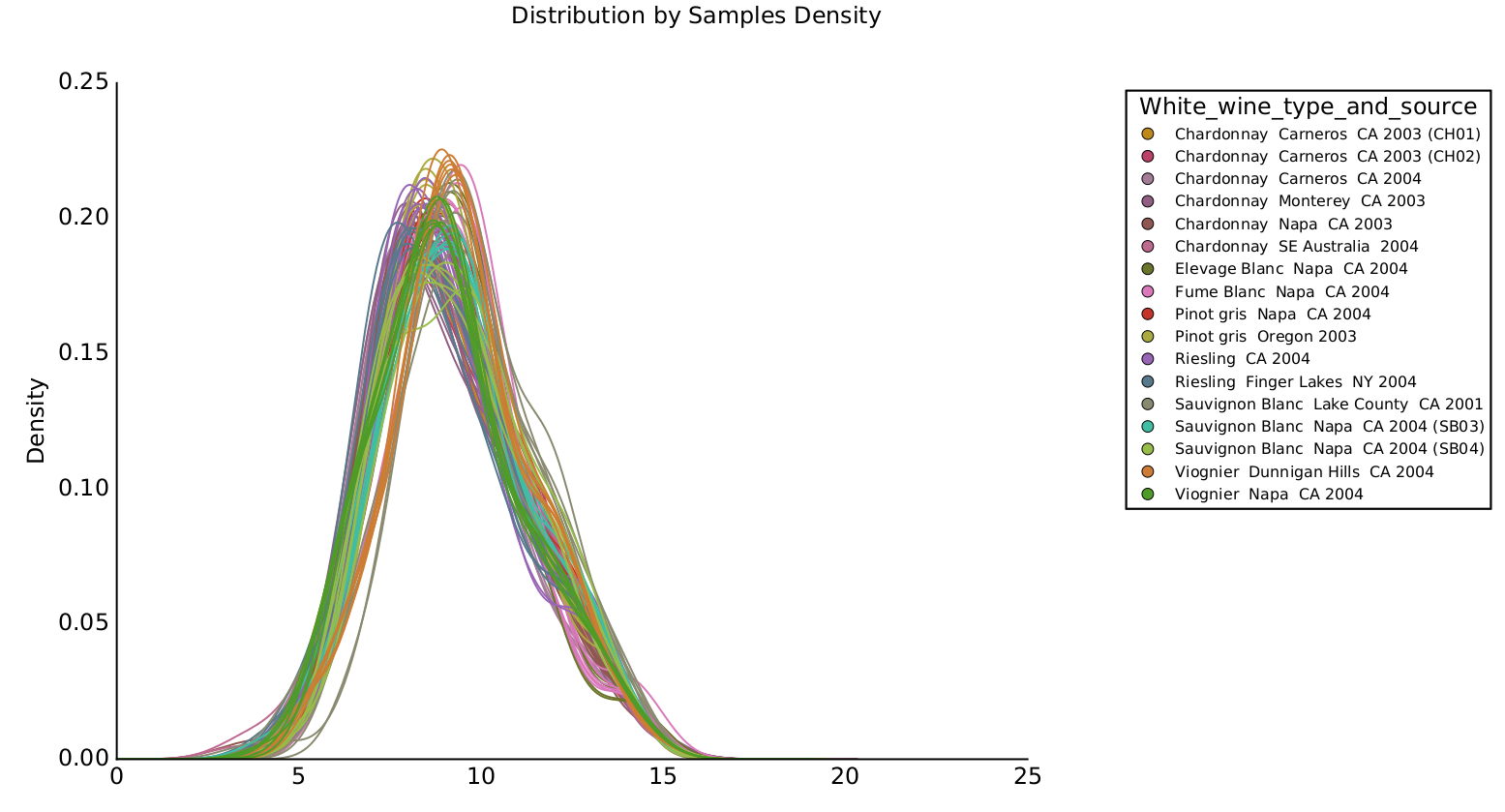


S8. Density plots for the distribution of features. Each line represents a sample and the colored reflects the group from the design file.


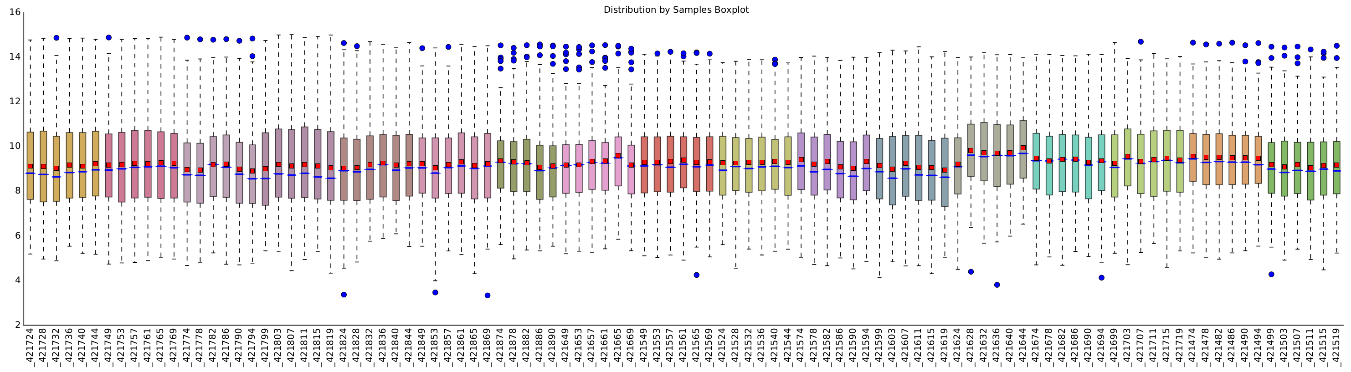


S9. Boxplots of the distribution of features for each sample. Each boxplot is color coded based on the group indicated by the design file.

**Standardize Euclidean Distance (SED)**

**Input Files:**

- Data file: ST000006_data.tsv
- Design file: ST000006_design.tsv

**Tool options:**


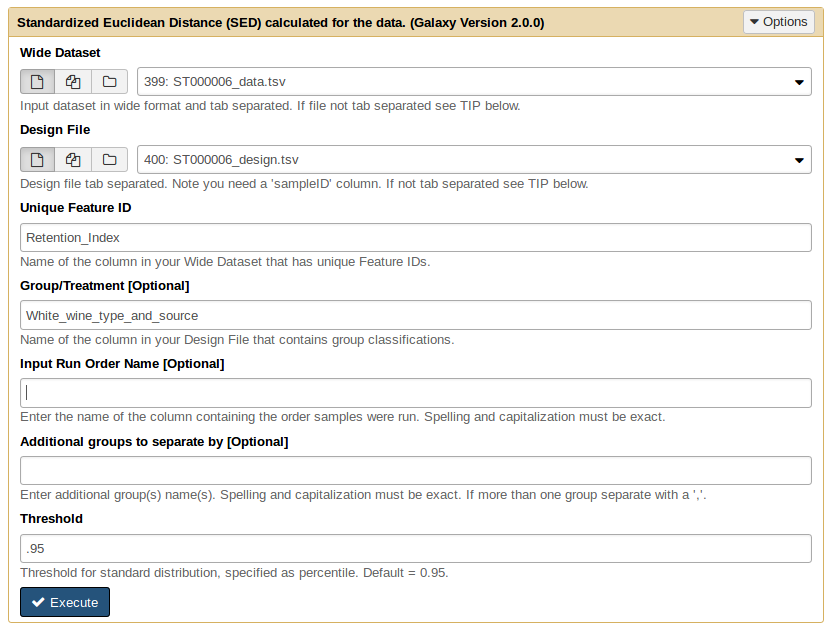


**Output:**


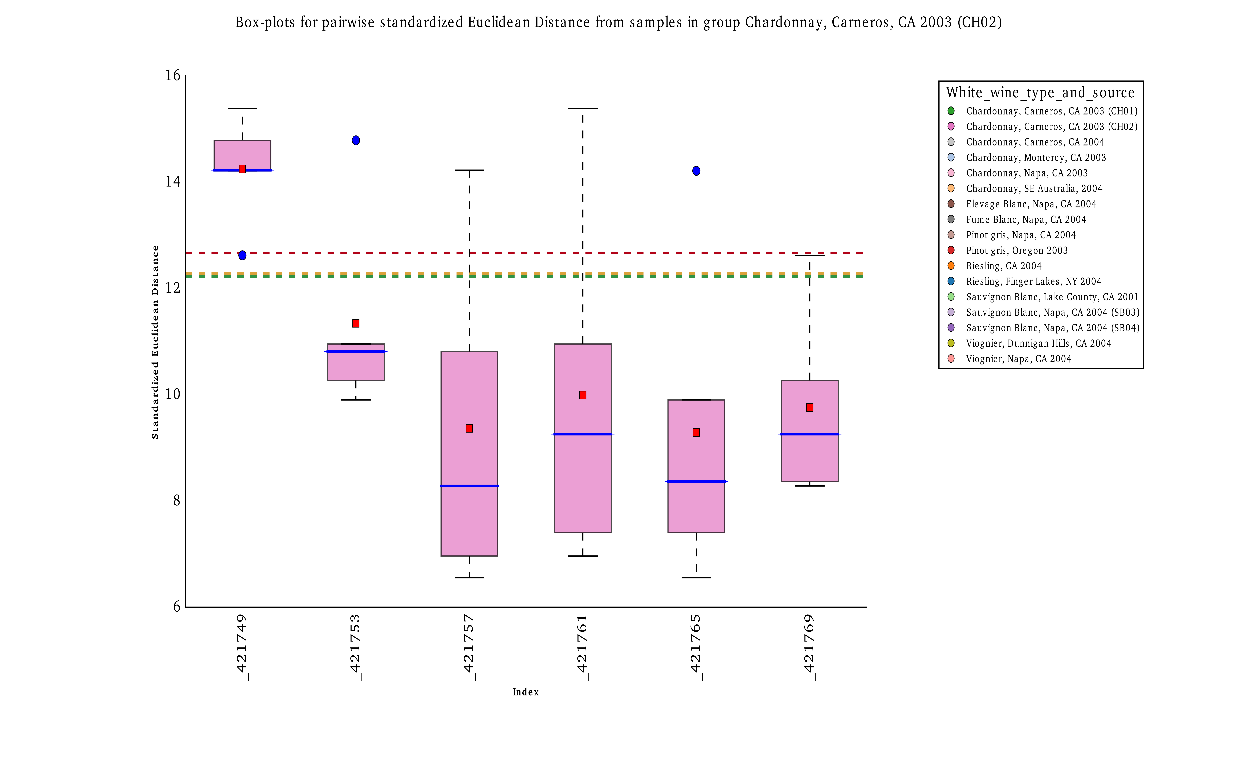


SF10. Euclidean distances are computed pairwise for each sample within a group. All pairwise distances computed are summarized for each sample as boxplots. Potential outliers (blue dots), means (red squares), and median (dark blue bars) are displayed. The threshold to declare a potential outlier is specified as threshold (specified as a percentile) in the input. This example shows the Standardized Euclidean Distances in the “Chardonnay, Carneros, CA 2003 (CH02)” group. The dashed lines correspond to the cutoffs are computed from beta, normal and chi-squared distributions in red, yellow and green respectively. Please note that normal and chi-squared cutoffs are expected to be very close to each other.


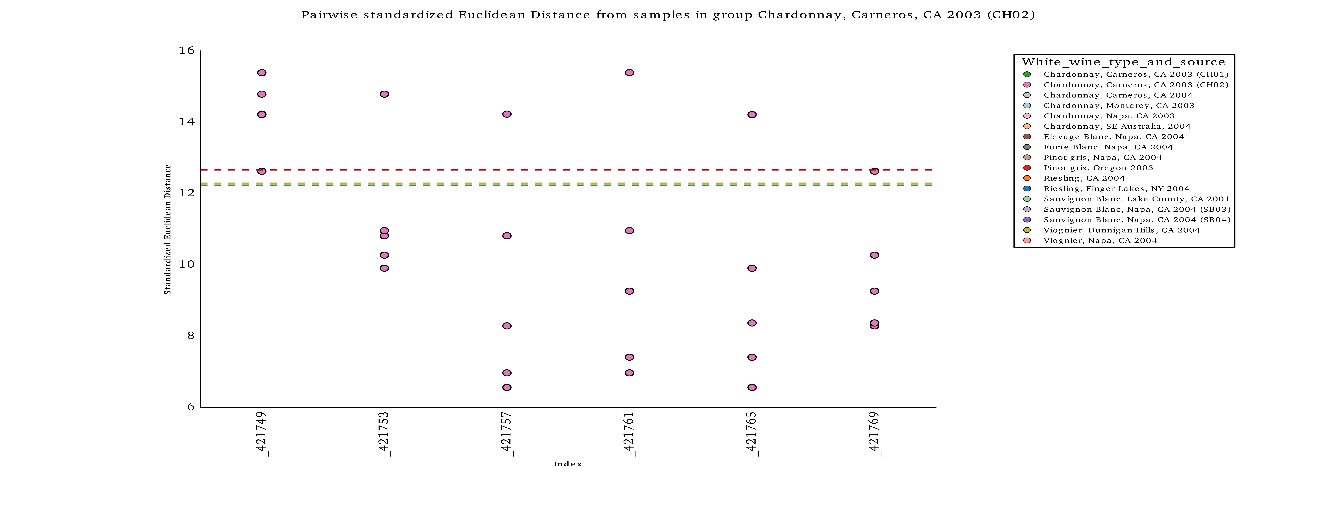


SF11. Figure showing a scatterplot of the Standardized Euclidean Distances in the “Chardonnay, Carneros, CA 2003 (CH02)” group. Distances are computed pairwise between samples within a group. The dashed lines correspond to the cutoffs are computed from beta, normal and chi-squared distributions in red, yellow and green respectively. Please note that normal and chi-squared cutoffs are expected to be very close to each other.**Penalized Mahalanobis Distance (PMD)**

**Input Files:**

- Data file: ST000006_data.tsv
- Design file: ST000006_design.tsv

**Tool options:**


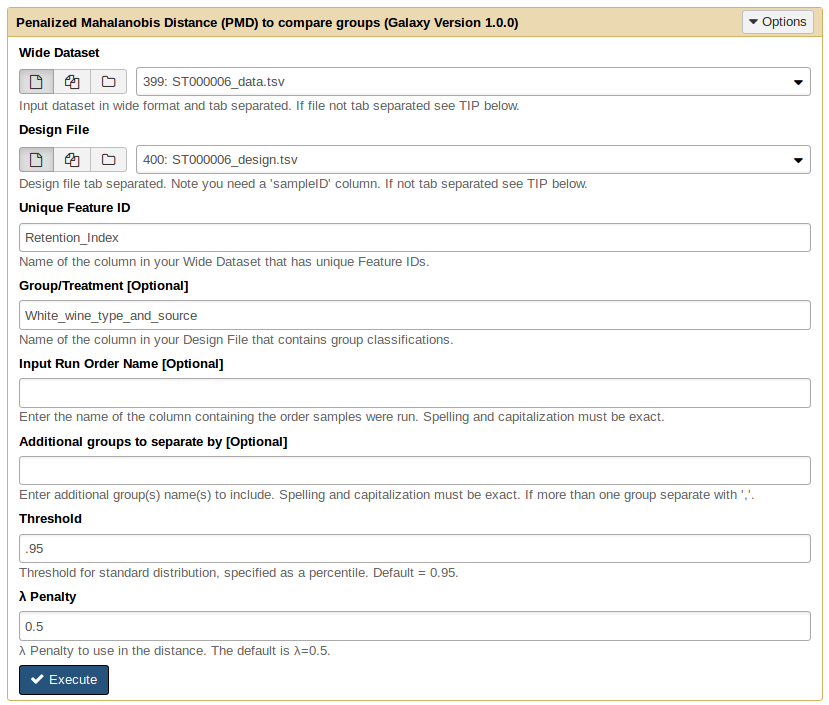


**Output:**


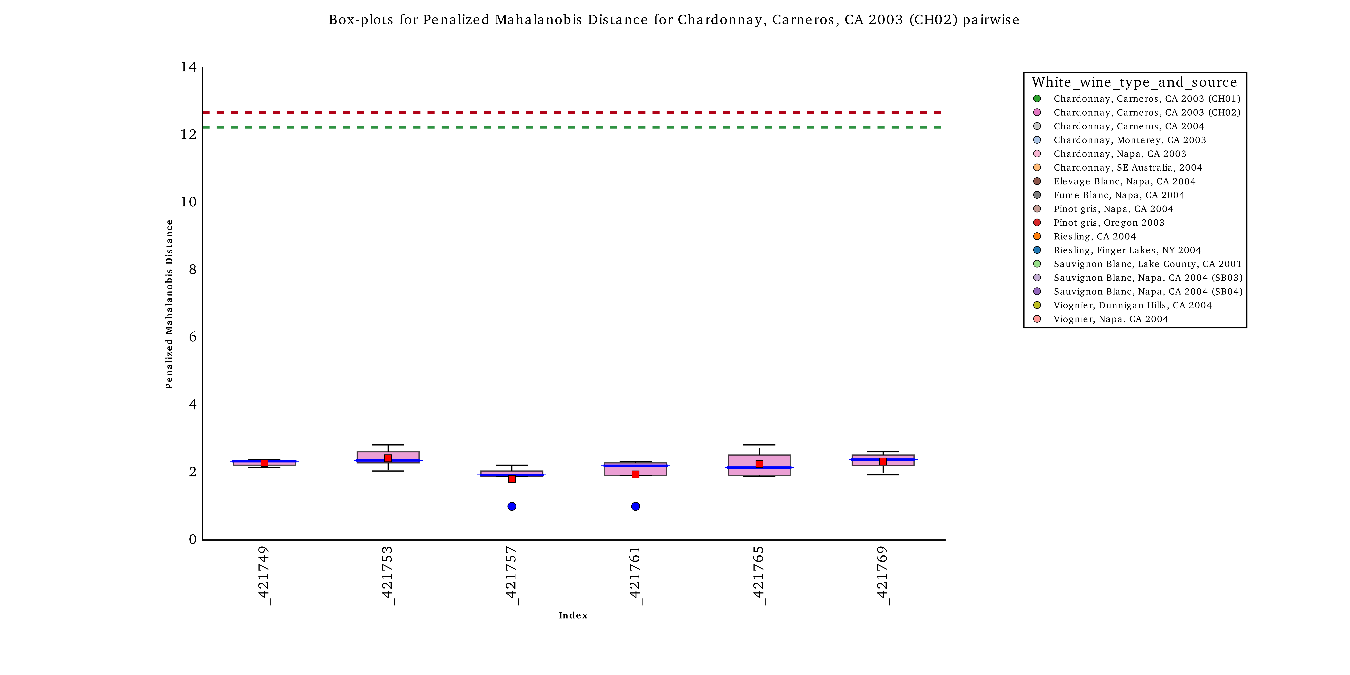


SF12 Penalized Mahalanobis distances are computed pairwise for each sample within a group. All pairwise distances computed are summarized for each sample as boxplots. Potential outliers (blue dots), means (red squares), and median (dark blue bars) are displayed. The threshold to declare a potential outlier is specified as threshold (specified as a percentile) in the input. This example shows the Standardized Euclidean Distances in the “Chardonnay, Carneros, CA 2003 (CH02)” group. The dashed lines correspond to the cutoffs are computed from beta, normal and chi-squared distributions in red, yellow and green respectively. Please note that normal and chi-squared cutoffs are expected to be very close to each other.


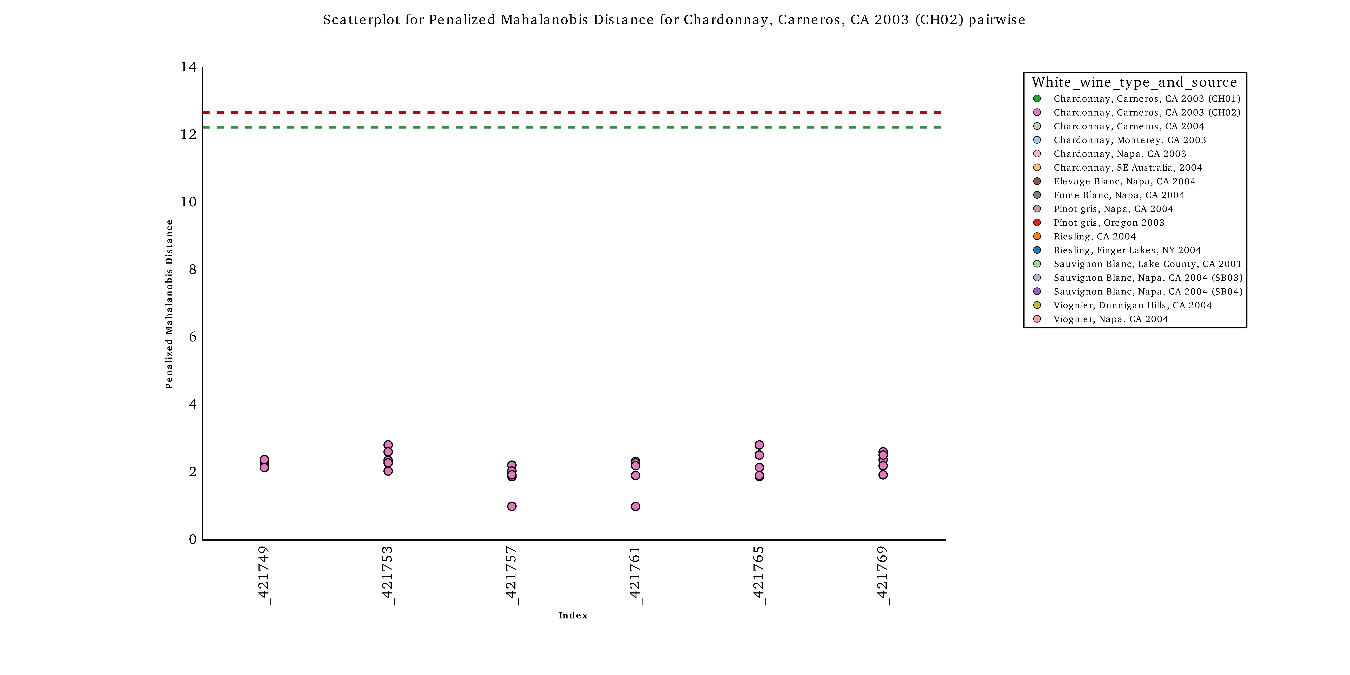


SF13 A scatterplot of the Penalized Mahalanobis Distances in the “Chardonnay, Carneros, CA 2003 (CH02)” group. Distances are computed pairwise between samples within a group. The dashed lines correspond to the cutoffs are computed from beta, normal and chi-squared distributions in red, yellow and green respectively. Please note that normal and chi-squared cutoffs are expected to be very close to each other.

**Analysis of Variance (ANOVA) Fixed Models**

**Input Files:**

- Data file: ST000006_data.tsv
- Design file: ST000006_design.tsv

**Tool options:**


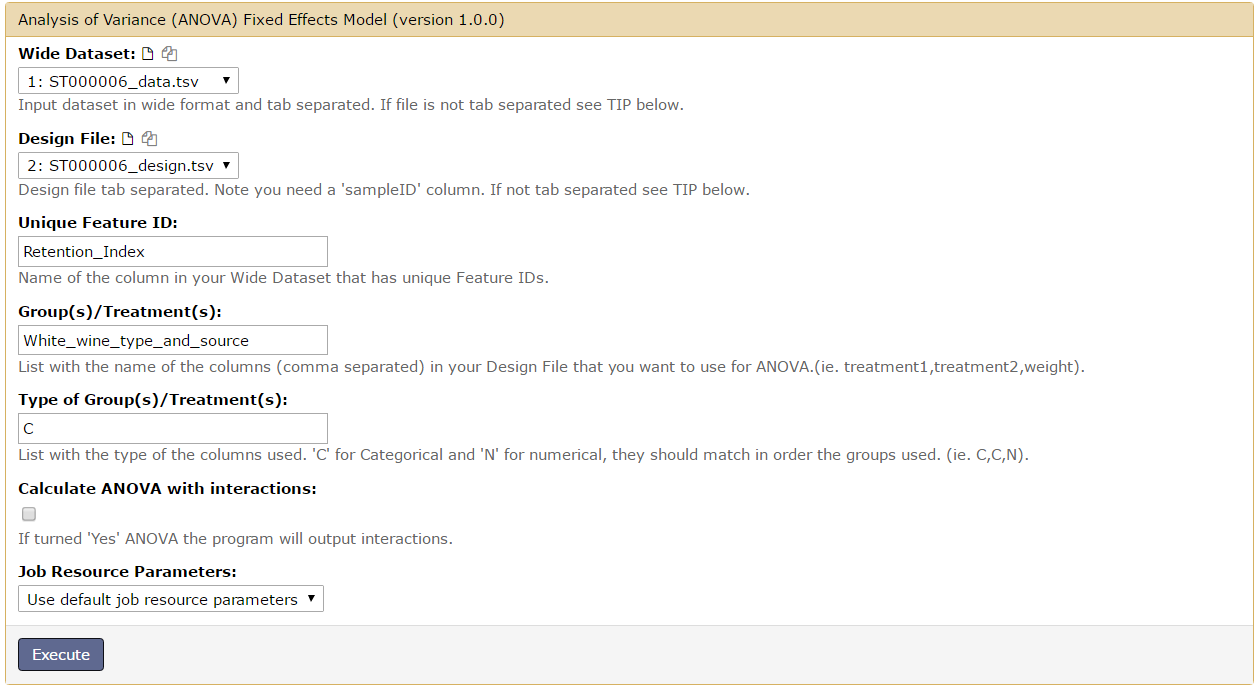


**Output:**


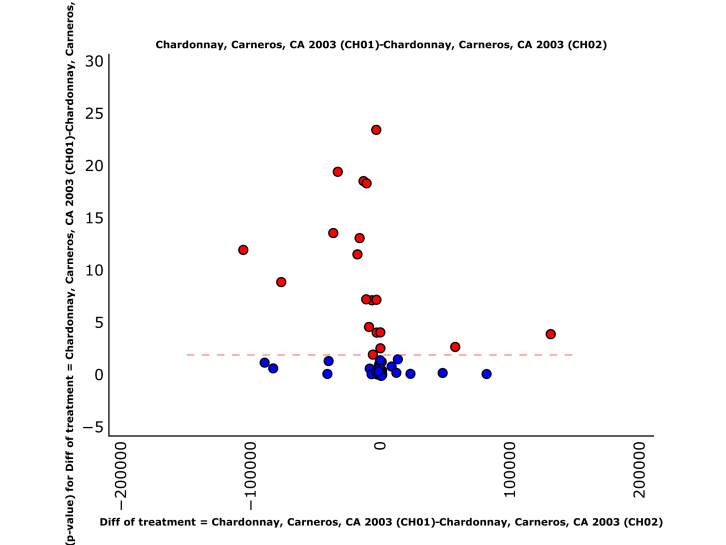


SF14. Volcano plot. One plot for each pairwise contrast is generated. On $x$-axis the difference between the pair of groups is displayed. On the $y$-axis the $p$-value for the test that the group means are equal is displayed on the negative $log$ base 10 scale. Each dot represents a feature. The red dashed line in the volcano plot(s) corresponds to a $p$-value = 0.01 (2 on the negative $log$ base 10 scale).


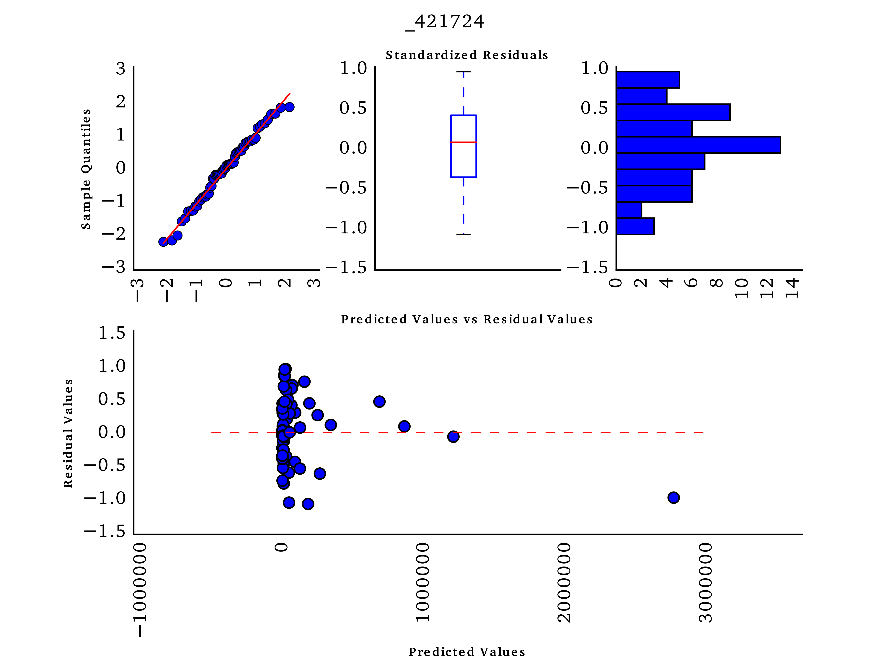


SF15. The Quantile-Quantile Plot (QQ Plot) is generated by the ANOVA Fixed Effects Models (top left). The QQ plot displays the expected quantiles of a normal distribution on $x$-axis versus the observed quantiles on $y$-axis. A boxplot (top center) of the standardized residuals and corresponding histogram of the residuals (top right). In the bottom figure the $x$-axis contains the predicted values, the corresponding standardized residuals are on the $y$-axis.

**Hierarchical Clustering Heatmap**

**Input Files:**

- Data file: ST000006_data_log.tsv
- Design file: ST000006_design.tsv

**Tool options:**


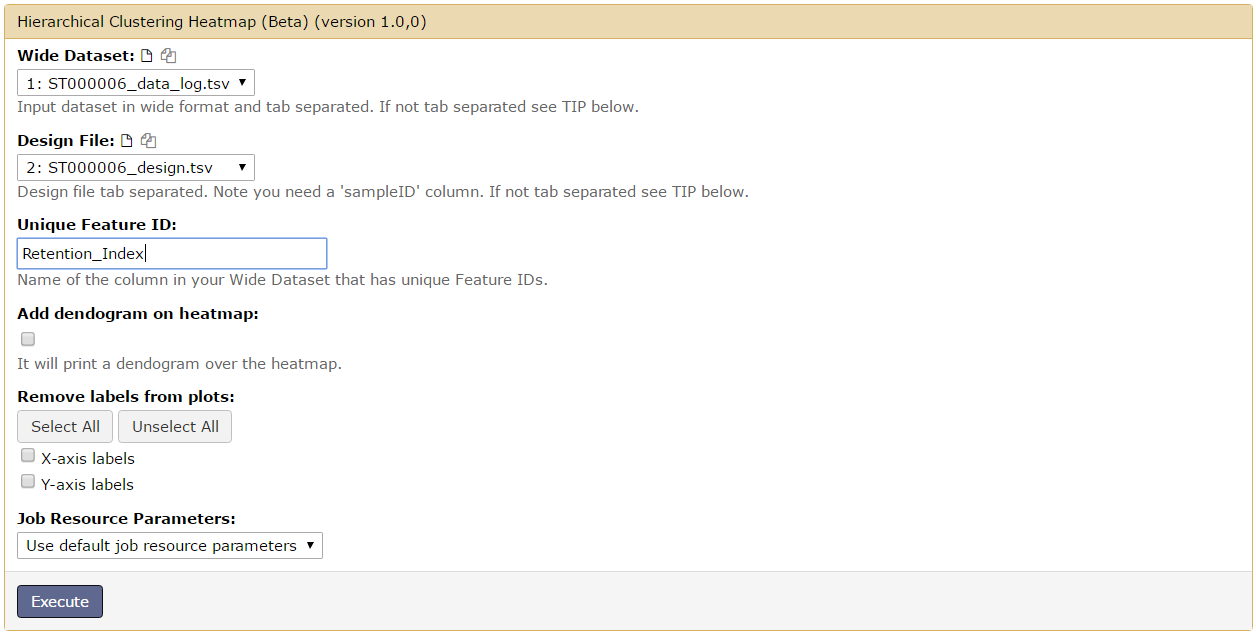


**Output:**


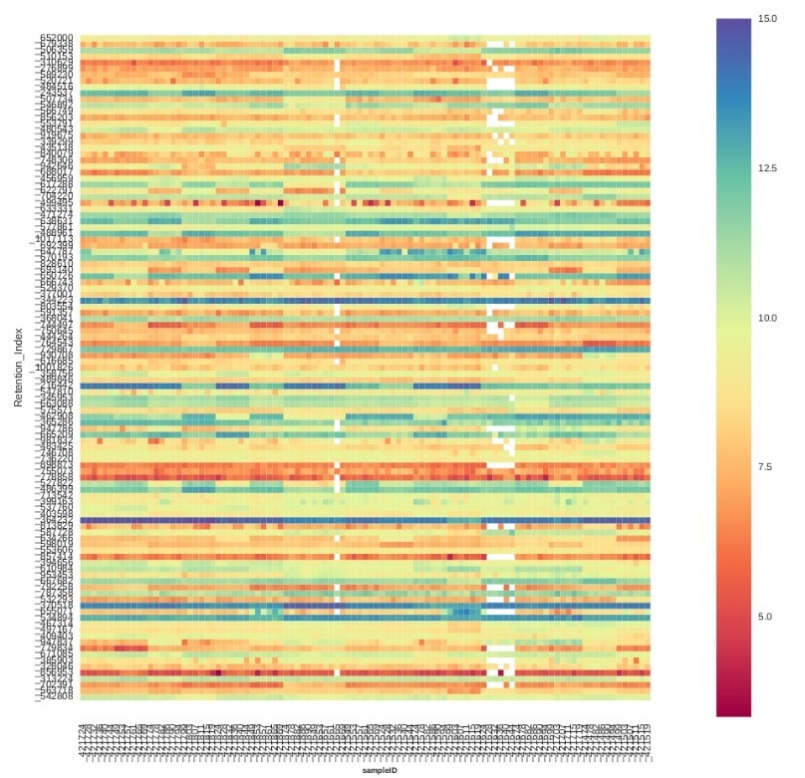


S16. Heatmap without dendrograms. The white spots are missing data.

**Input Files:**

- Data file: ST000006_data_log.tsv
- Design file: ST000006_design.tsv

**Tool options:**


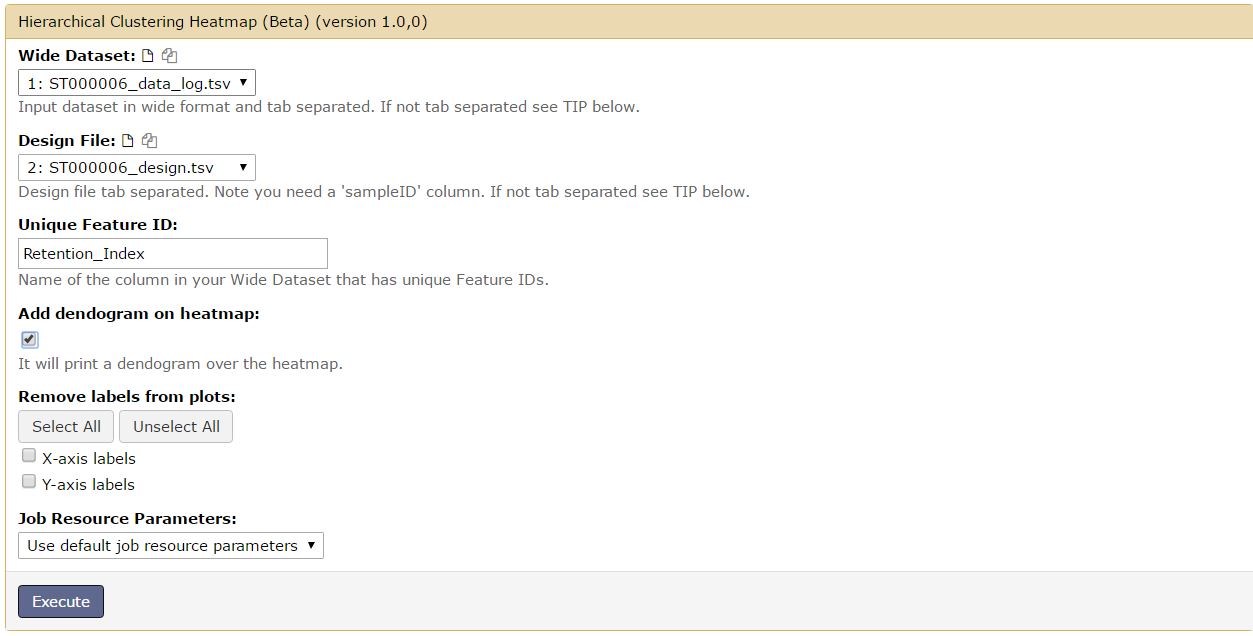


**Output:**


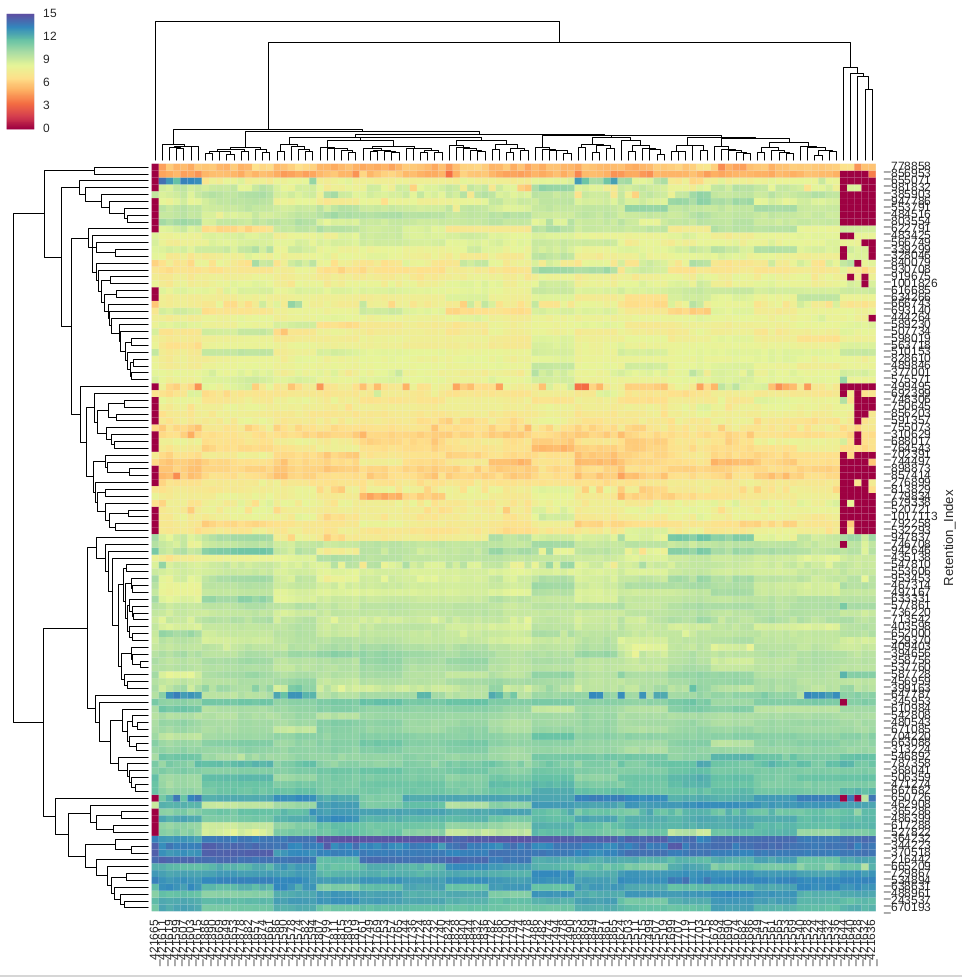


S17. Hierarchical cluster heatmap with dendrograms. The missing data are represented with dark red.

**Modulated Modularity Clustering (MMC)**

**Input Files:**

- Data file: ST000006_data.tsv
- Design file: ST000006_design.tsv

**Tool options:**


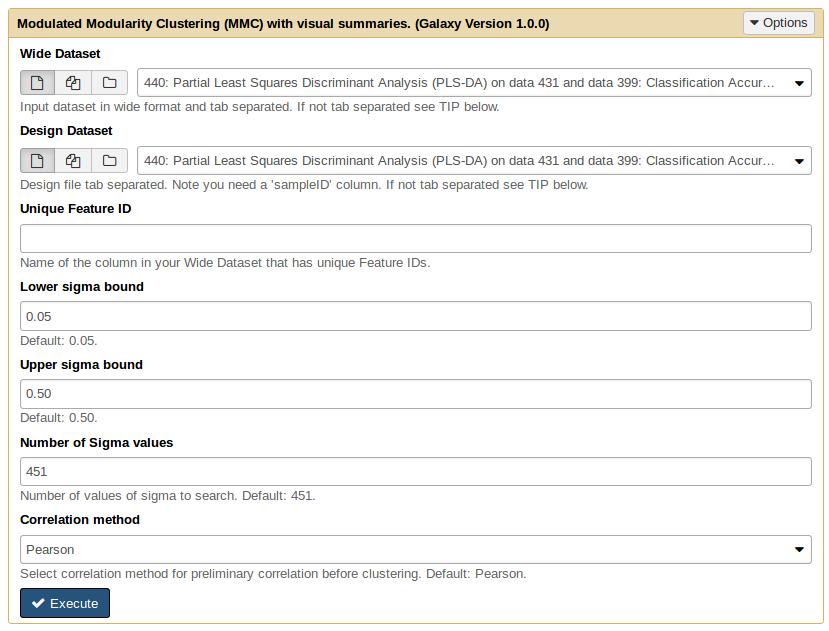


**Output:**


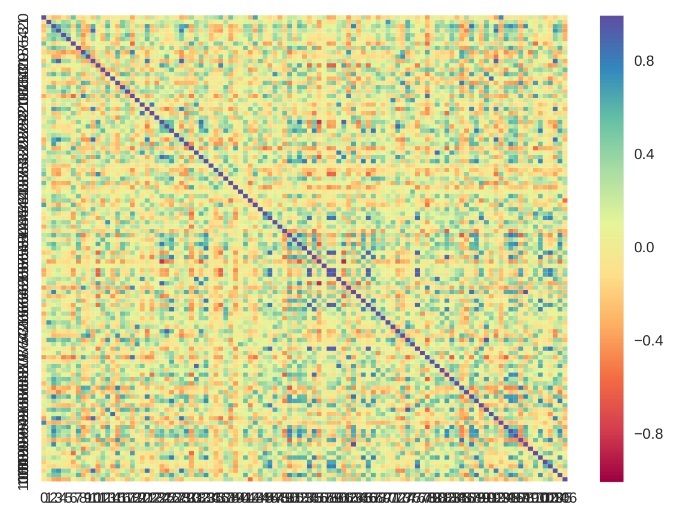

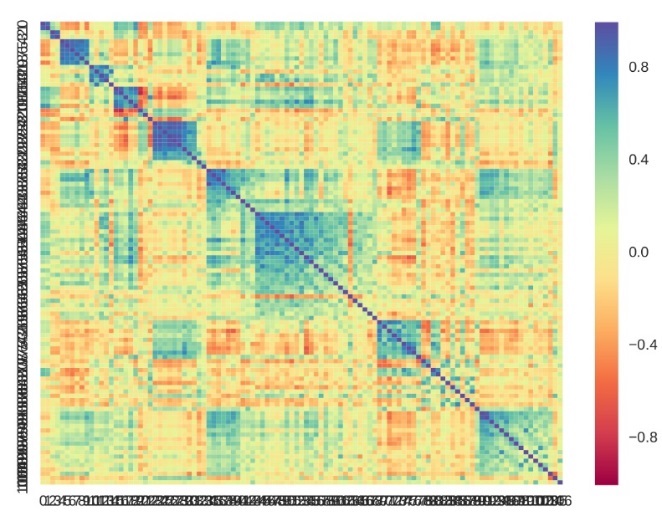

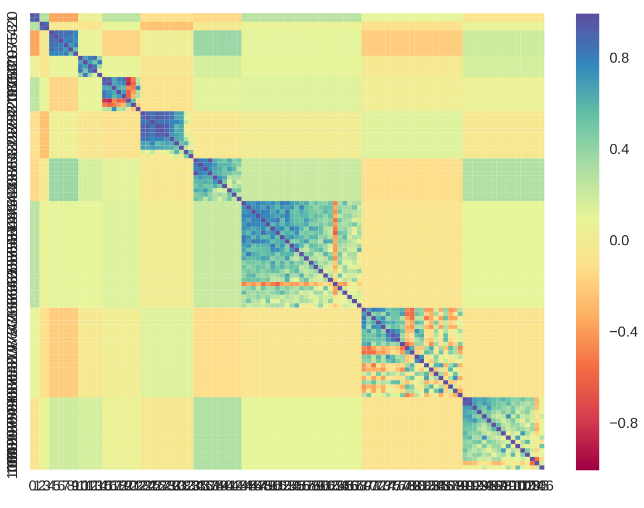


SF18 The heatmaps show the original variance-covariance matrix (top left), the rearranged variance covariance matrix (top right) and the smoothed version of the rearranged variance covariance matrix (bottom).

**Principal Component Analysis (PCA)**

**Input Files:**

- Data file: ST000006_data.tsv
- Design file: ST000006_design.tsv

**Tool options:**


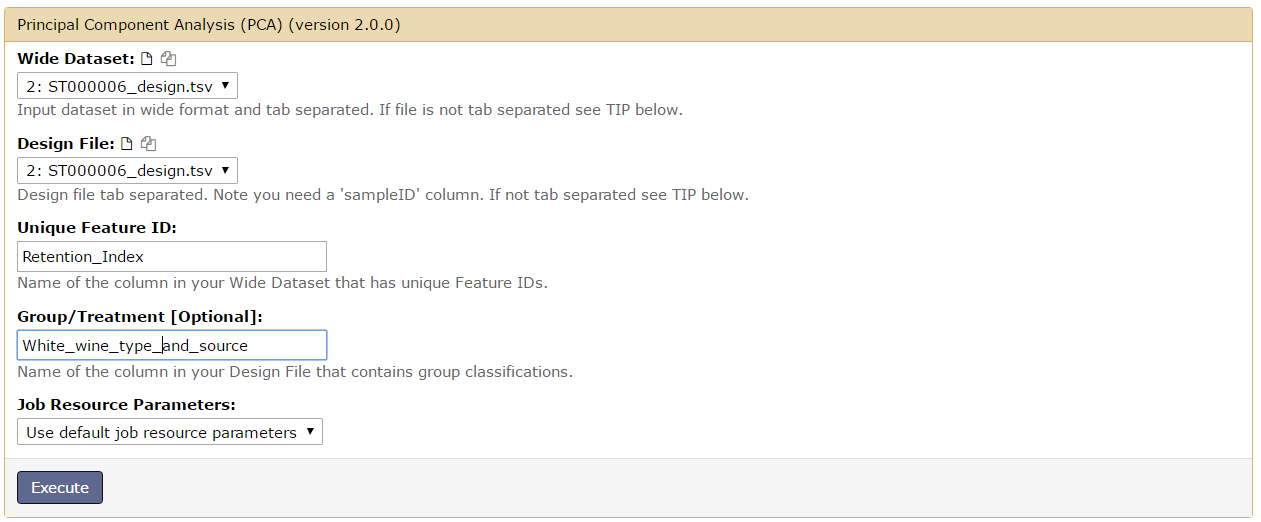


**Output:**


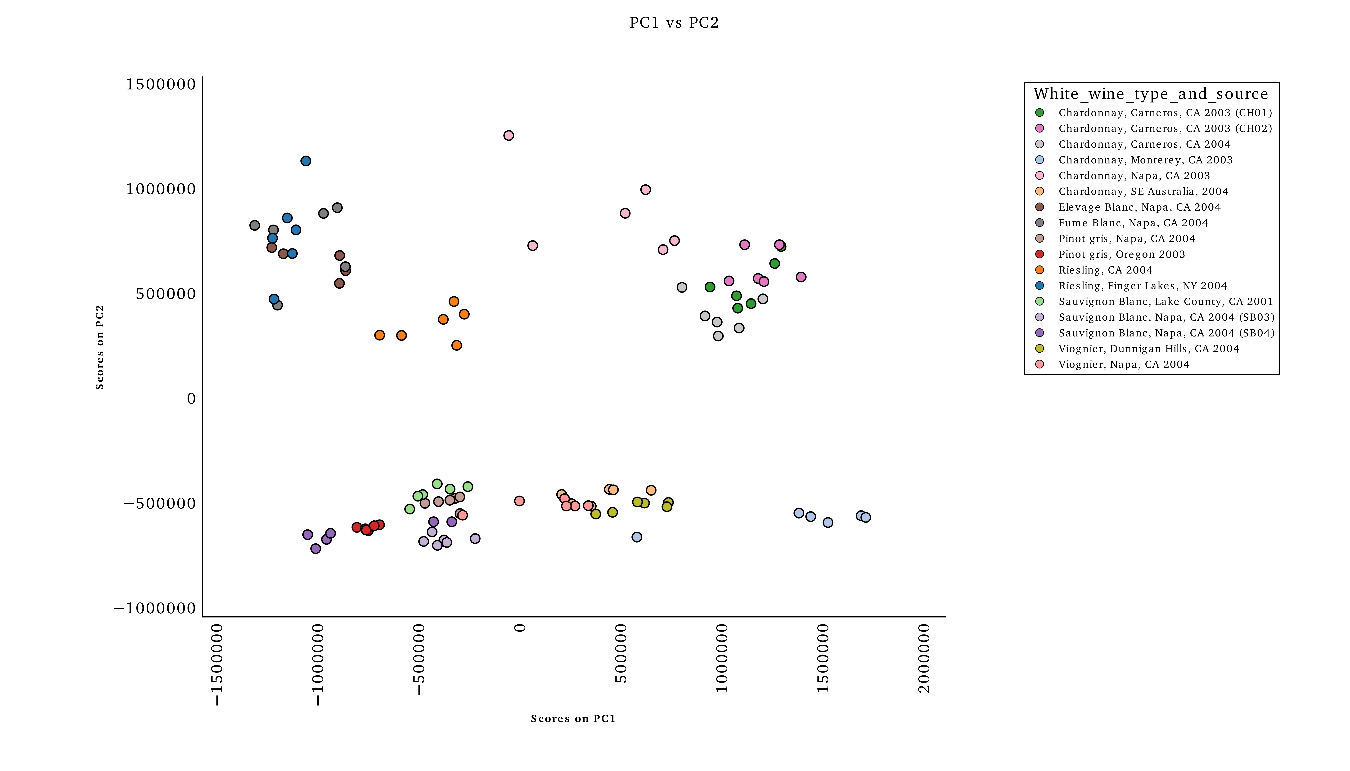


SF19. Scatterplot of the first two principal components. Each sample is represented as a point. The samples are color coded based on their group.


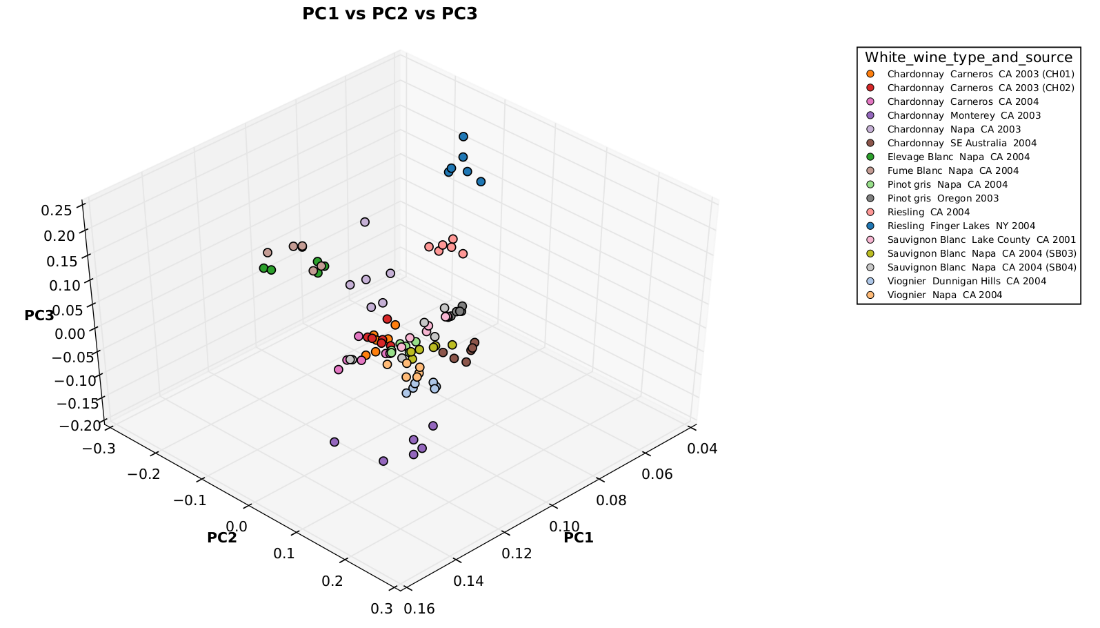


SF20. The 3D scatterplot shows the first three principal components. Each sample is represented as a point. The samples are color coded based on group.

**Partial Least Squares Discriminant Analysis (PLS-DA)**

**Input Files:**

- Data file: ST000006_data.tsv
- Design file: ST000006_design.tsv

**Tool options:**


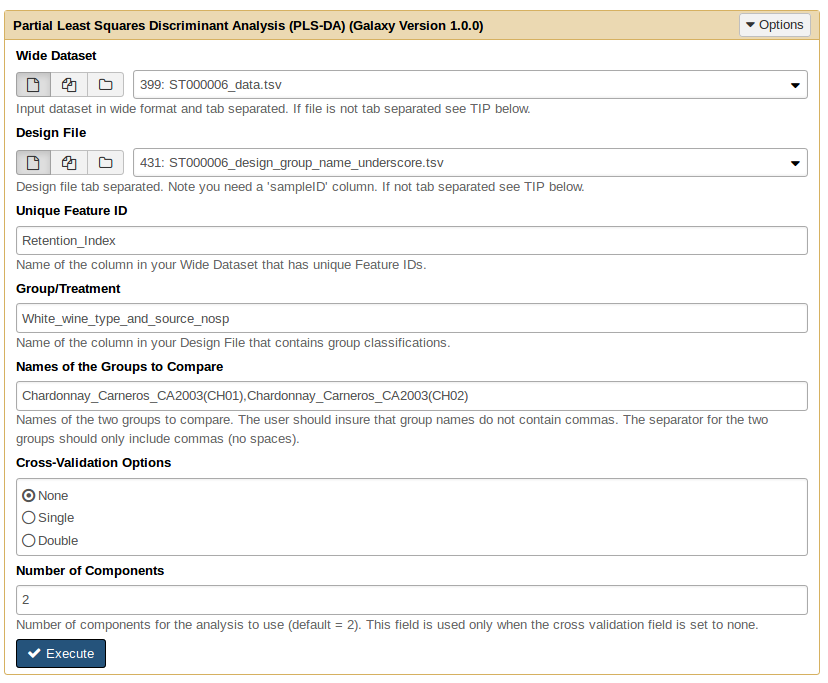


**Output:**


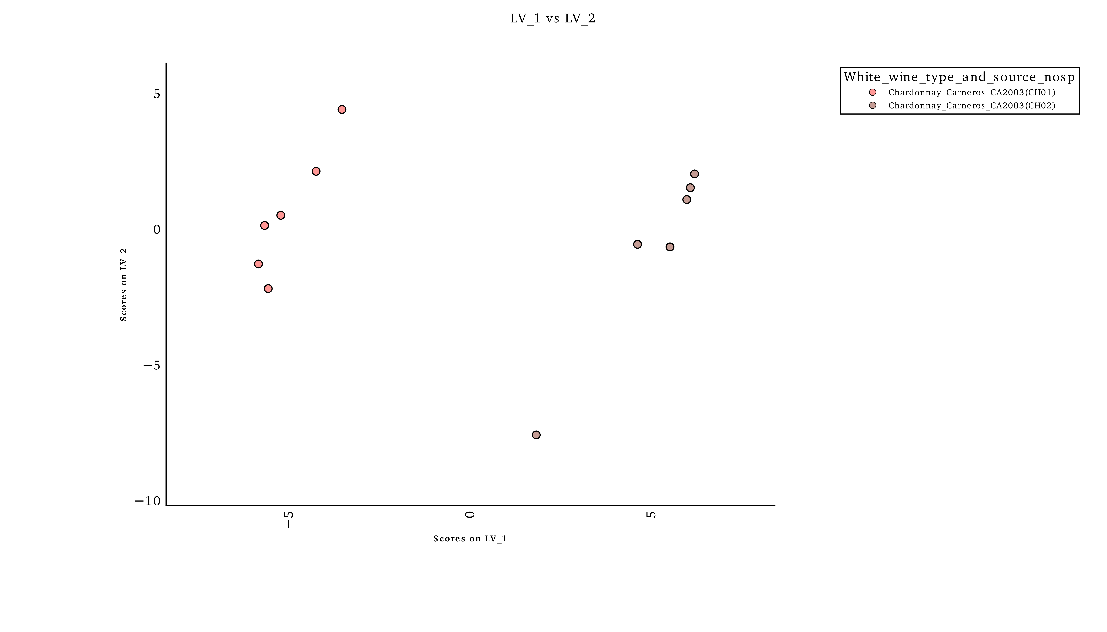


SF21. The plot corresponds to a pairwise comparison between groups: “Chardonnay_Carneros_CA2003(CH01)” and “Chardonnay_Carneros_CA2003(CH01)”.

**Linear Discriminant Analysis (LDA)**

**Input Files:**

- Data file: ST000006_data.tsv
- Design file: ST000006_design.tsv

**Tool options:**


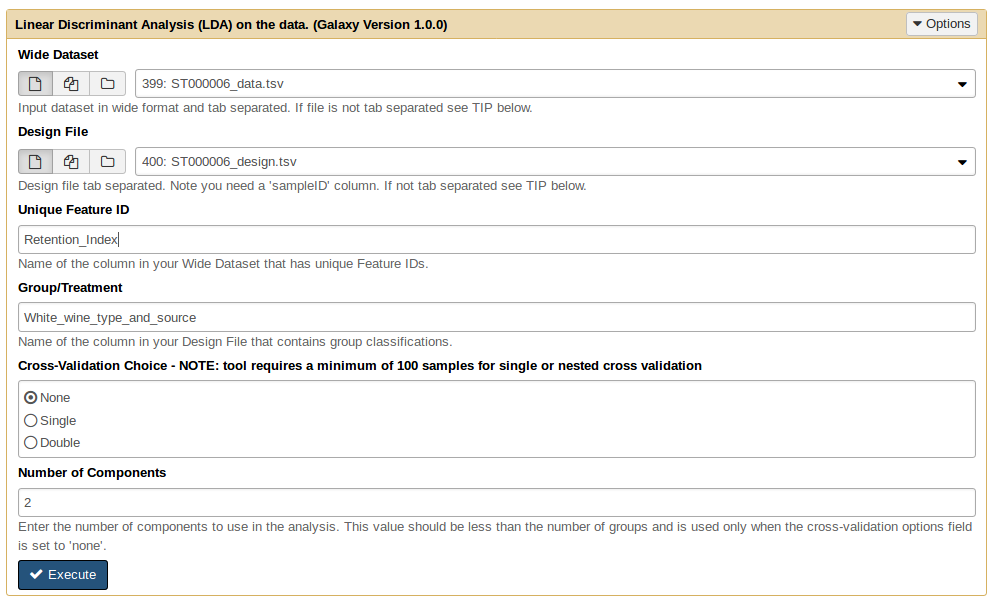


**Output:**


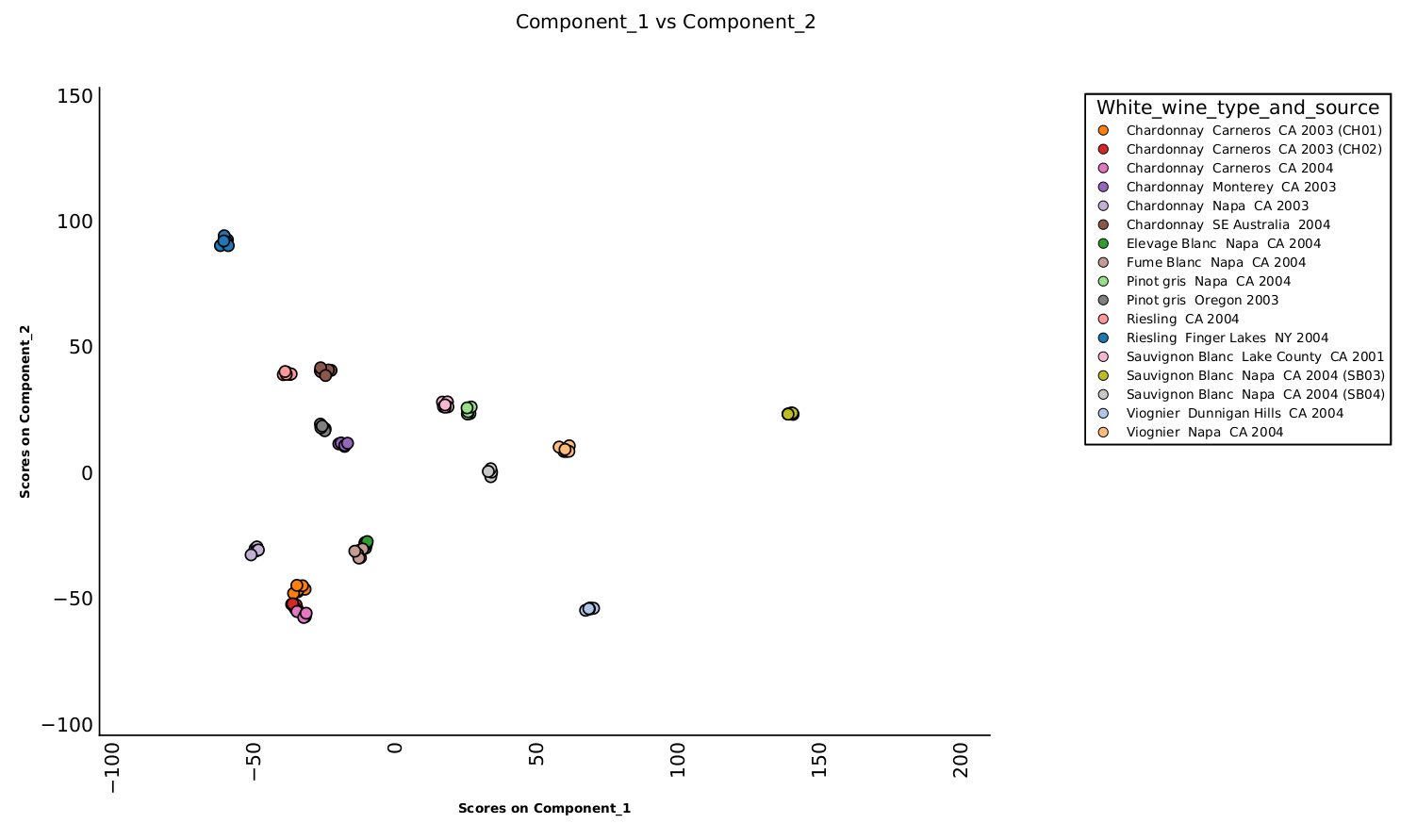


SF22. Scatterplot for the first two components of each sample. The samples are color coded based on group.

**Random Forest (RF)**

**Input Files:**

- Data file: ST000006_data.tsv
- Design file: ST000006_design.tsv

**Tool options:**


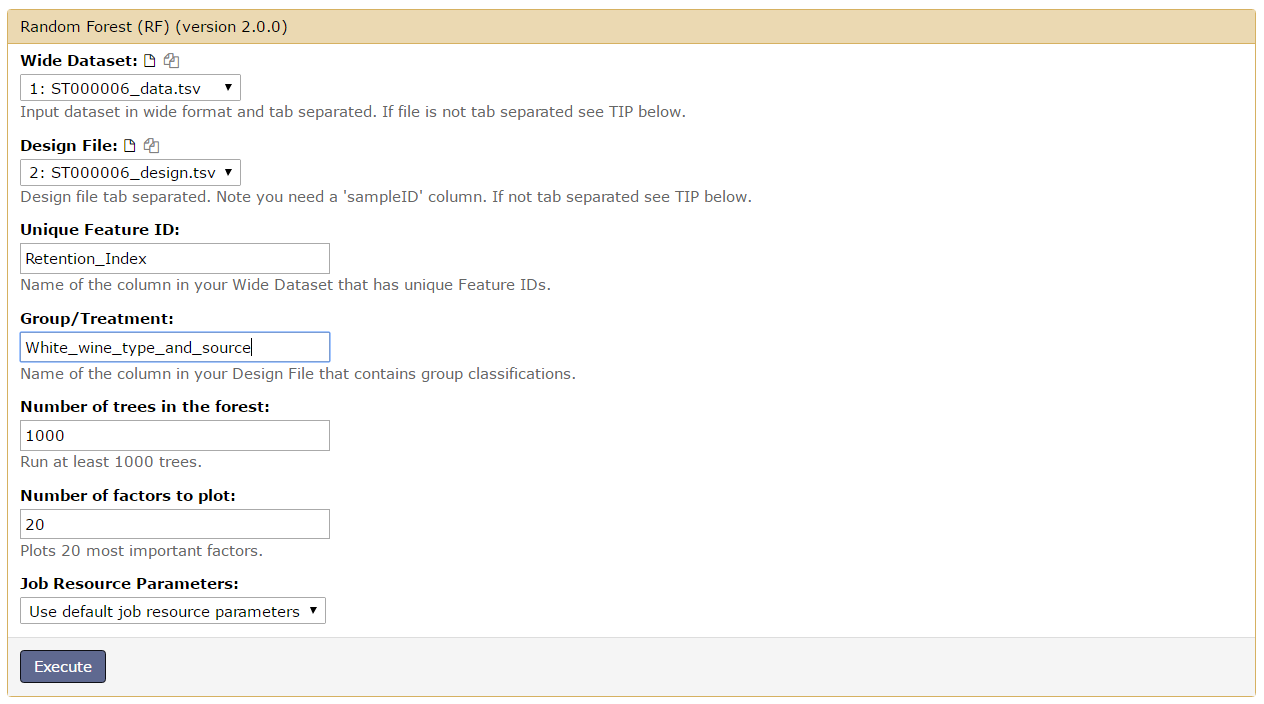


**Output:**


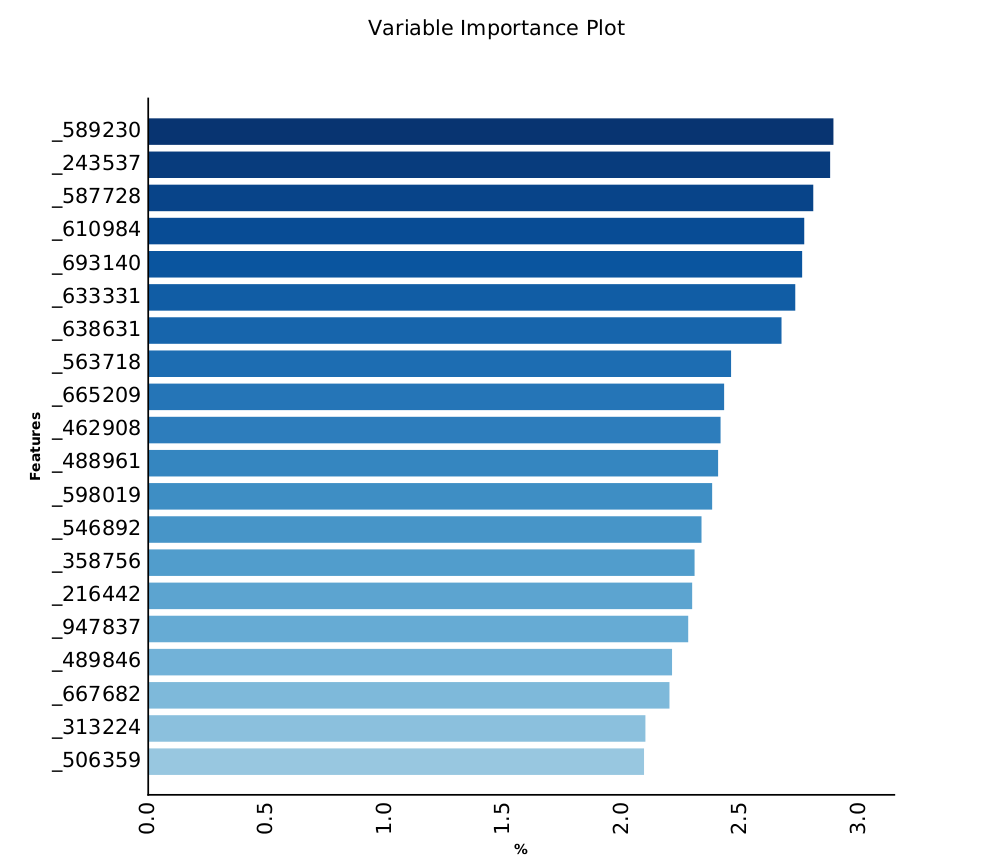


SF23. The variable importance plot displays 20 features with the largest relative importance values ($x$-axis), identified by the random forest algorithm. The color of each feature is scaled from (dark blue) to (light blue).

**LASSO/Elastic Net Variable Selection**

**Input Files:**

- Data file: ST000006_data.tsv
- Design file: ST000006_design.tsv

**Tool options:**


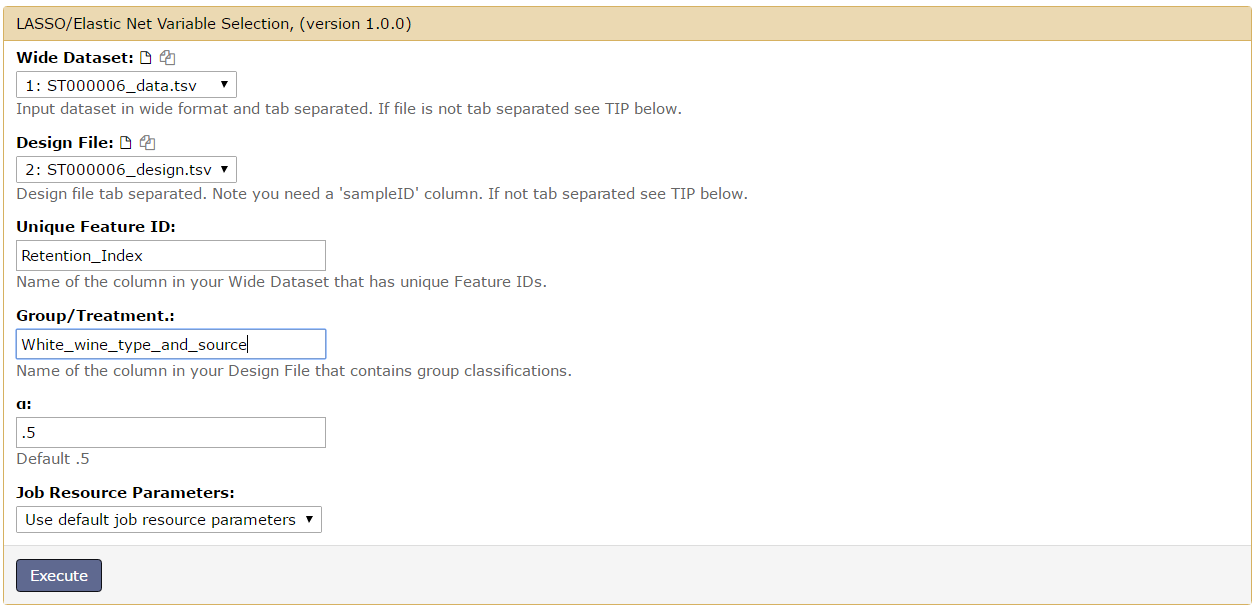


**Output:**


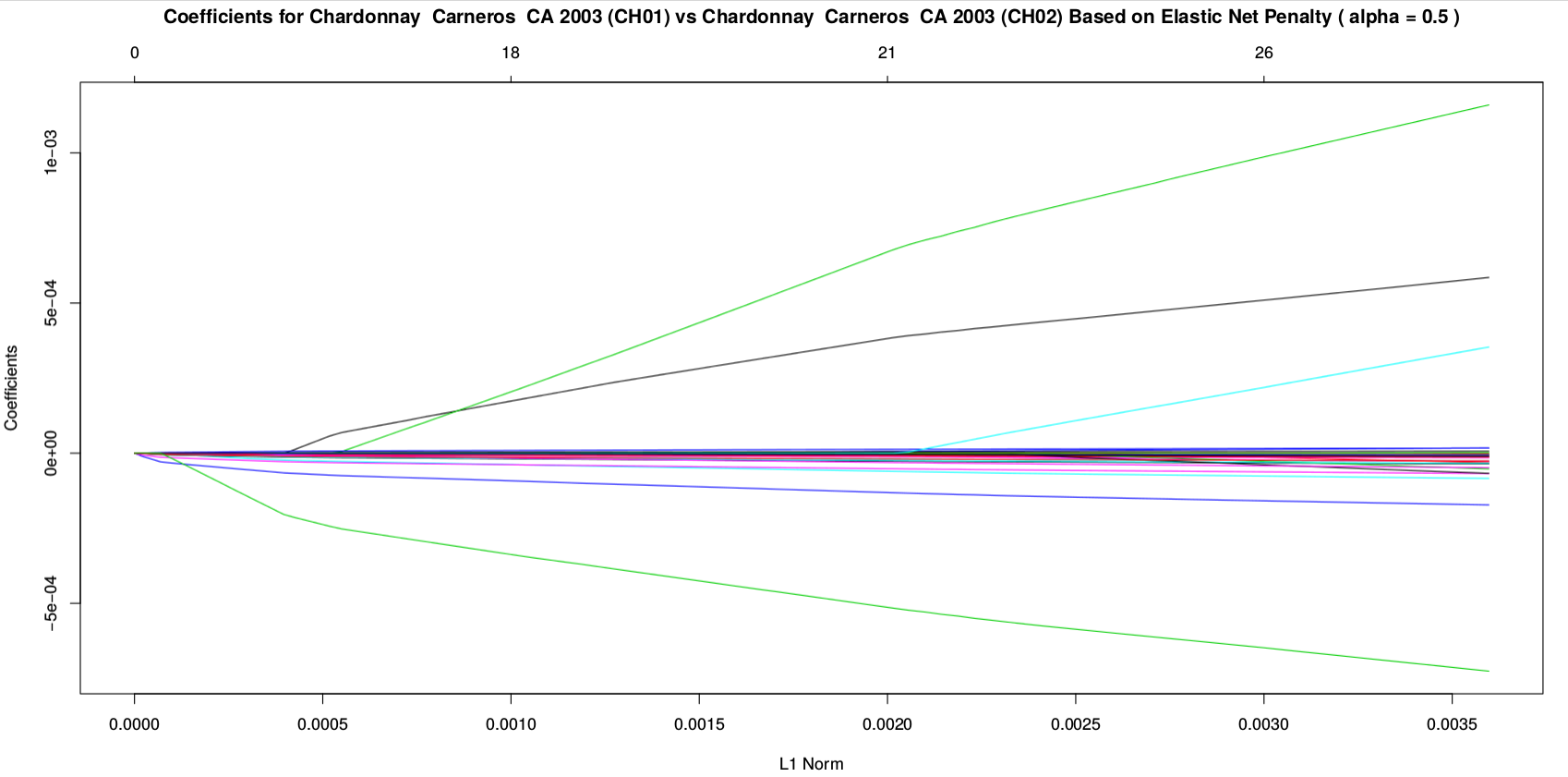


SF24. The plot shows the behavior of the LASSO/Elastic Net coefficients based on the value of penalty $\lambda$ for penalty split parameter $\alpha=0.5$. The value$\alpha=1$ corresponds to the LASSO penalty. The value of the penalty parameter $\lambda=0$ forces all the regression coefficients to be zero. The optimal value for the penalty $\lambda$ is determined by a cross-validation procedure. For easier visualization the line for each coefficient (for each feature) has its own color.


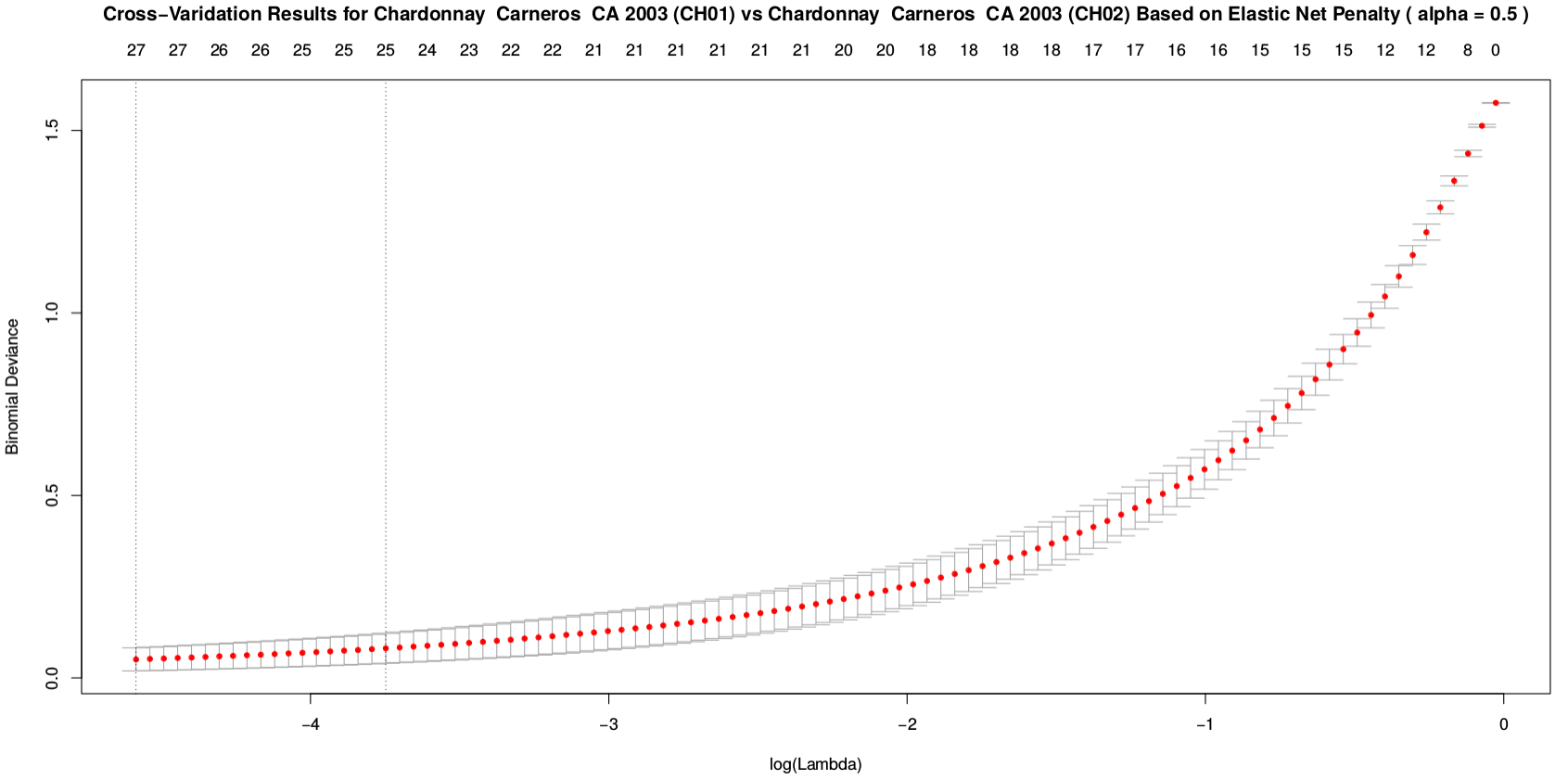


SF25. The value of the penalty parameter $\lambda$ on the log scale is plotted along the $x$-axis. The corresponding cross validation error (binomial deviance) is plotted along the $y$-axis. The number of features that corresponds to the given value of penalty $log(\lambda)$is displayed on the top of the graph along the $x$-axis. The two vertical lines correspond to $\lambda$-s picked by the cross-validation algorithm. The first $\lambda$ (dotted line on the left) corresponds to the smallest mean cross-validation error. The second $\lambda$ (dotted line on the right) corresponds to the most regularized model with the error within one standard deviation from the minimum error.

More details about the Elastic Net and LASSO methods can be found in the reference below:

Tibshirani, Robert. "Regression shrinkage and selection via the lasso." Journal of the Royal Statistical Society. Series B (Methodological) (1996): 267-288.

Zou, H., and Hastie, T. (2005). Regularization and variable selection via the elastic net. Journal of the Royal Statistical Society: Series B (Statistical Methodology), 67(2), 301-320.

Friedman, Jerome, Trevor Hastie, and Rob Tibshirani. "Regularization paths for generalized linear models via coordinate descent." Journal of statistical software 33, no. 1 (2010): 1.

**T-Test (Single Group)**

**Input Files:**

- Data file: ST000006_data.tsv
- Design file: ST000006_design.tsv

**Tool options:**


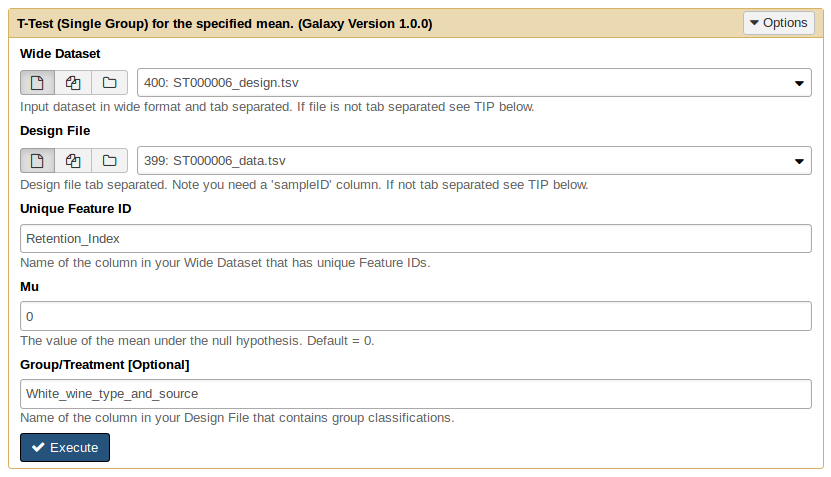


**Output:**


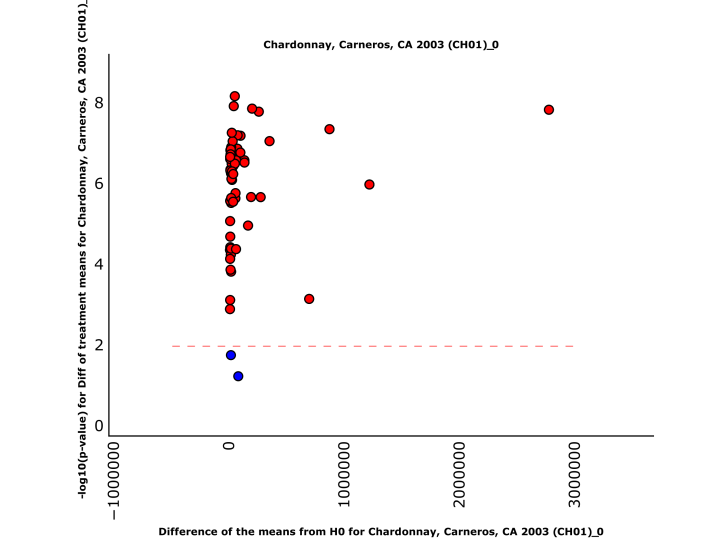


SF26. Volcano plot. On the $x$-axis the difference between the group mean and the value under the null (mu) is displayed. On the $y$-axis the $p$-value for the test that the group means are equal is displayed on the negative $log$ base 10 scale. Each dot represents a feature. The red dashed line in the volcano plot(s) corresponds to a $p$-value = 0.01 (2 on the negative $log$ base 10 scale).

**T-Test**

**Input Files:**

- Data file: ST000006_data.tsv
- Design file: ST000006_design.tsv

**Tool options:**


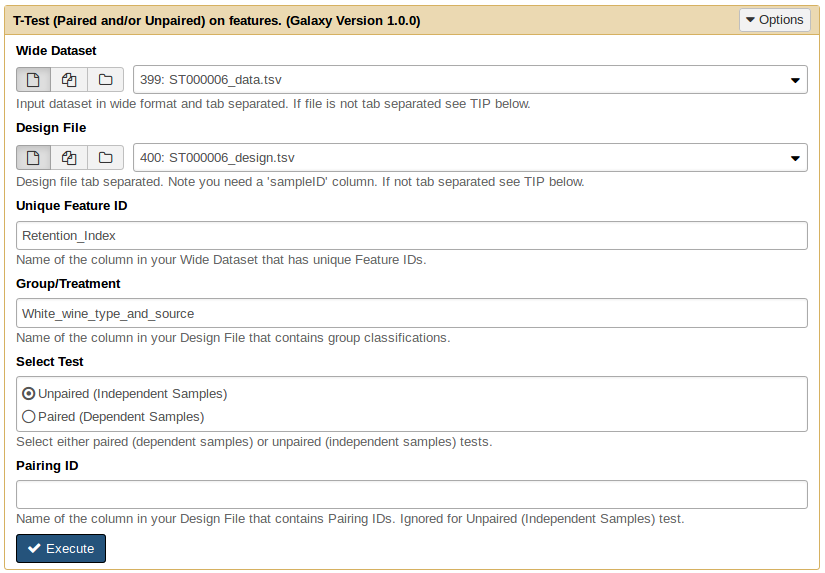


**Output:**


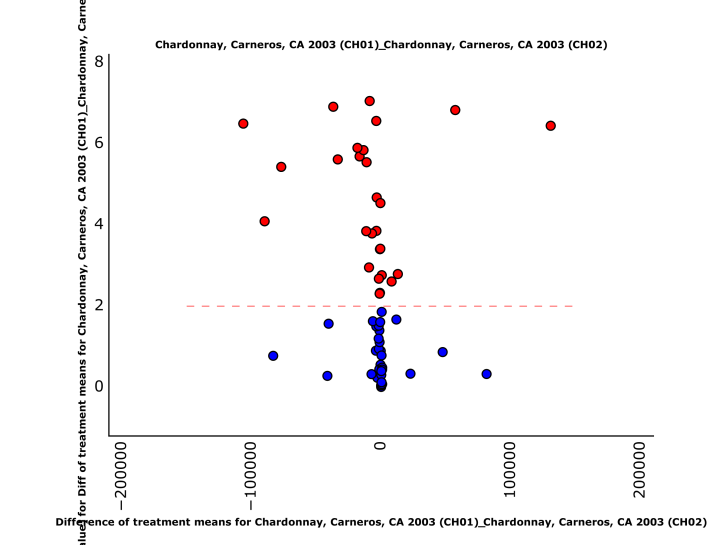


SF27.

Volcano plot. One plot for each pairwise comparison is generated for unpaired test. On the $x$-axis the difference between the group means is displayed. On the $y$-axis the $p$-value for the test that the group means are equal is displayed on the negative $log$ base 10 scale. Each dot represents a feature. The red dashed line in the volcano plot(s) corresponds to a $p$-value = 0.01 (2 on the negative $log$ base 10 scale).

**Kruskal-Wallis Non-Parametric Test**

**Input Files:**

- Data file: ST000006_data.tsv
- Design file: ST000006_design.tsv

**Tool options:**


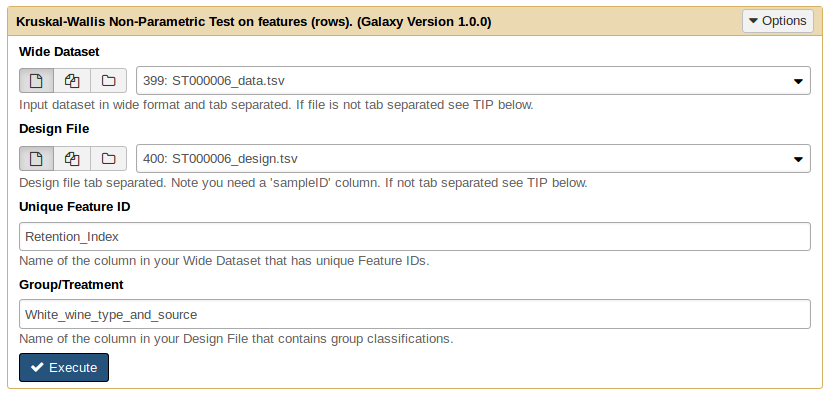


**Output:**


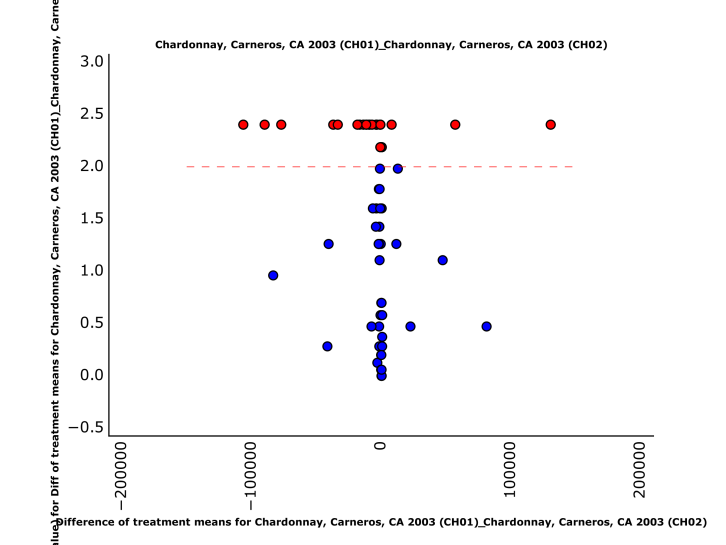


SF28. Volcano plot. One plot for each pairwise comparison is generated. On the $x$-axis the difference between the pair of groups is displayed. On the $y$-axis the $p$-value for the test that the group means are equal is displayed on the negative $log$ base 10 scale. Each dot represents a feature. The red dashed line in the volcano plot(s) corresponds to a $p$-value = 0.01 (2 on the negative $log$ base 10 scale).

**MZ-RT Matching**

**Input Files:**

- Anno File 1: TEST0000_mzrt_first.tsv
- Anno File 2: TEST0000_mzrt_second.tsv

**Tool options:**


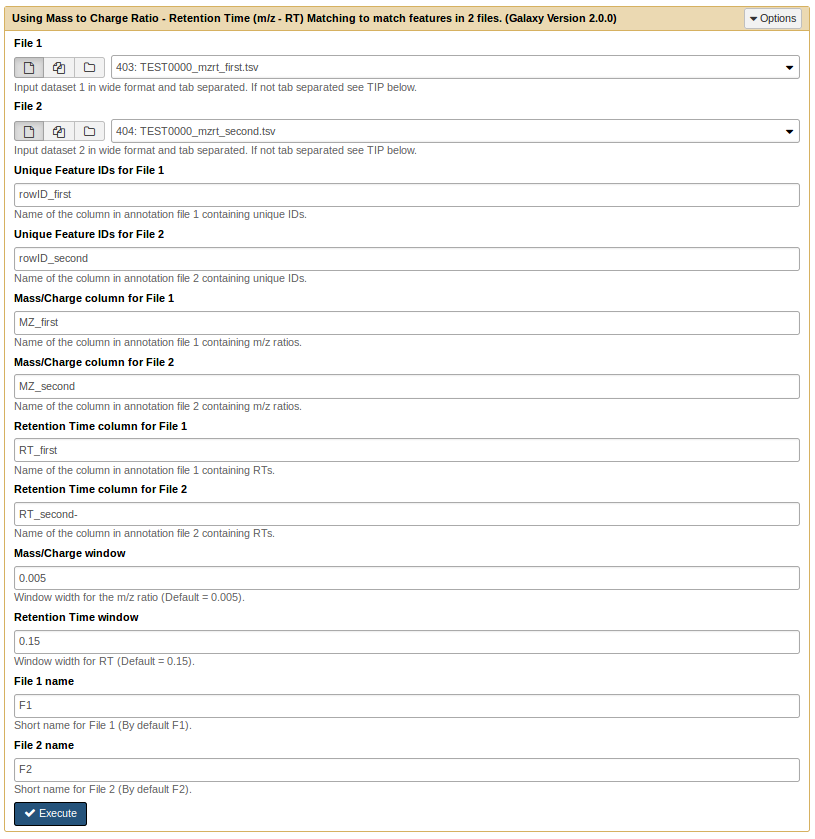


**Output:**


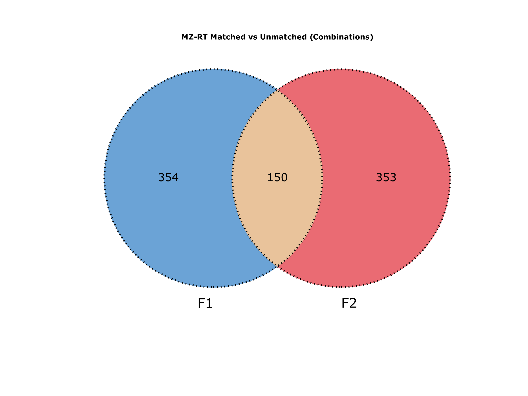


SF29. Venn diagram of the matched vs unmatched combinations for the two files.


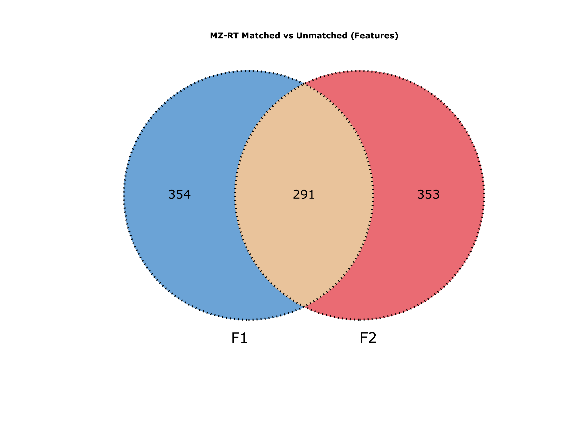


SF30. Venn diagram of the matched vs unmatched features for the two files.


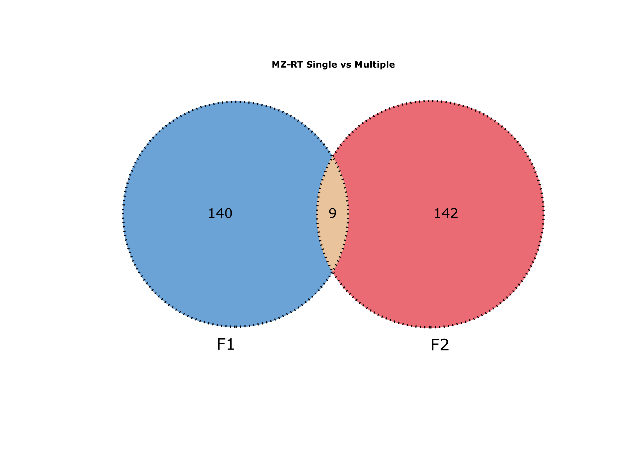


SF31. Venn diagram of the single vs multiple matches for the two files.
