# Supplementary material for: SECIMTools: a suite of metabolomics data analysis tools
Source: BMC Bioinformatics. 2018 Apr 20;19:151. doi: 10.1186/s12859-018-2134-1 (PMC5910624; doi:10.1186/s12859-018-2134-1)
Supplement: Supplementary file 3 — Mahalanobis Distance calculation. (PDF 102 kb) [file 12859_2018_2134_MOESM3_ESM.pdf]

Penalized Mahalanobis Distance

Theoretical Justification

Let $\boldsymbol{X}$ be $n \times p$ dimensional matrix with $n > p$. The primary interest is the distances between the columns of the matrix $\boldsymbol{X}$ using the correlation structure between the rows of the matrix $\boldsymbol{X}$. The singular value decomposition (SVD) for $\boldsymbol{X}$ has the form $\boldsymbol{U}\boldsymbol{S}\boldsymbol{V}^T$. Here $\boldsymbol{U}\boldsymbol{U}^T = \boldsymbol{I}$ and $\boldsymbol{V}\boldsymbol{V}^T = \boldsymbol{I}$ are the orthogonal matrices of dimension $n \times n$ and $p \times p$ respectively. The matrix $\boldsymbol{S}$ has dimension $n \times p$ and has non-zero elements only on diagonal when row indexes are equal to column indexes. The rank of $\boldsymbol{S}$ is equal to the rank of $\boldsymbol{X}$. Also let $\boldsymbol{X}\boldsymbol{X}^T = \boldsymbol{U}\boldsymbol{D}\boldsymbol{U}^T$ be a spectral decomposition (SD) of $\boldsymbol{X}\boldsymbol{X}^T$. The matrix $\boldsymbol{D}$ is a square diagonal matrix with dimension $n \times n$. The relationship between two decompositions is the following:

$$\boldsymbol{X}\boldsymbol{X}^T = \left[\boldsymbol{U}\boldsymbol{S}\boldsymbol{V}^T\right]\left[\boldsymbol{U}\boldsymbol{S}\boldsymbol{V}^T\right]^T = \boldsymbol{U}\boldsymbol{S}\boldsymbol{V}^T\boldsymbol{V}\boldsymbol{S}^T\boldsymbol{U}^T = \boldsymbol{U}\boldsymbol{S}\boldsymbol{S}^T\boldsymbol{U}^T = \boldsymbol{U}\boldsymbol{D}\boldsymbol{U}^T \tag{1}$$

Since $n > p$, the number of non-zero diagonal elements can be at most p. The relationship between two decompositions imply $\boldsymbol{S}\boldsymbol{S}^T = \boldsymbol{D}$. The diagonal elements in $\boldsymbol{S}$ are not defined uniquely since the sign in SVD can be arbitrary. The diagonal elements of $\boldsymbol{D}$ however are defined uniquely and are all non-negative for non-negative definite matrices such as $\boldsymbol{X}\boldsymbol{X}^T$. We use the notations d_i and s_i^2 where $i = 1, 2, \ldots, n$ for diagonal elements of $\boldsymbol{D}$ and $\boldsymbol{S}\boldsymbol{S}^T$ respectively. The values $|s_i|$ are called singular values of the matrix $\boldsymbol{X}$ and they are related to eigenvalues d_i of matrix $\boldsymbol{X}\boldsymbol{X}^T$ using relationship $d_i = s_i^2$.

If we assume that matrix $\boldsymbol{X}\boldsymbol{X}^T$ is invertible (i.e. $n < p$ and row space has a full rank, which is *not* the case for our matrix) then all d_i-s are positive and the inverse matrix $\left[\boldsymbol{X}\boldsymbol{X}^T\right]^{-1}$ has the form

$$\left[\boldsymbol{X}\boldsymbol{X}^T\right]^{-1} = \boldsymbol{U}\boldsymbol{D}^{-1}\boldsymbol{U}^T = \boldsymbol{U}\left[\boldsymbol{S}\boldsymbol{S}^T\right]^{-1}\boldsymbol{U}^T \tag{2}$$

where

$$\boldsymbol{D}^{-1} = \text{diag}\left[\frac{1}{d_1}, \frac{1}{d_2}, \ldots, \frac{1}{d_n}\right]$$
$$\left[\boldsymbol{S}\boldsymbol{S}^T\right]^{-1} = \text{diag}\left[\frac{1}{s_1^2}, \frac{1}{s_2^2}, \ldots, \frac{1}{s_n^2}\right]$$

For the considered matrix $\boldsymbol{X}$ with dimensions $n \times p$ with $n > p$ the matrix $\boldsymbol{X}\boldsymbol{X}^T$ does not have a full rank and is *not* invertible. In this case the SVD decomposition of the inverse (2) does not make sense.

To fix that the penalty is introduced. The idea is to add penalty to the original matrix $\boldsymbol{X}\boldsymbol{X}^T$ to make it invertible. The suggested matrix is $\boldsymbol{X}\boldsymbol{X}^T + \lambda\boldsymbol{I}$ and the corresponding inverse has the form

$$[\boldsymbol{X}\boldsymbol{X}^T + \lambda\boldsymbol{I}]^{-1} = [\boldsymbol{U}\boldsymbol{D}\boldsymbol{U}^T + \boldsymbol{U}(\lambda\boldsymbol{I})\boldsymbol{U}^T]^{-1}$$
$$= \boldsymbol{U}\left[\text{diag}\left[\frac{1}{d_1+\lambda}, \frac{1}{d_2+\lambda}, \ldots, \frac{1}{d_n+\lambda}\right]\right]\boldsymbol{U}^T$$
$$= \boldsymbol{U}\left[\text{diag}\left[\frac{1}{s_1^2+\lambda}, \frac{1}{s_2^2+\lambda}, \ldots, \frac{1}{s_n^2+\lambda}\right]\right]\boldsymbol{U}^T$$

The matrix $\boldsymbol{X}\boldsymbol{X}^T + \lambda\boldsymbol{I}$ is invertible and the inverse $[\boldsymbol{X}\boldsymbol{X}^T + \lambda\boldsymbol{I}]^{-1}$ exists provided $\lambda \neq 0$. The question is how to select appropriate penalty λ? Intuitively, the smallest eigenvalues d_i are the least important ones. Those values experience maximal shrinkage d_i and the largest values of $\frac{1}{d_i}$ which degenerates to ∞ when $d_i = 0$. To follow the logic the reasonable choose is to shrink based on the smallest d_i in the dataset which is *not* equal to 0. That implies

$$\lambda := \min\{d_i : d_i \neq 0\}.$$

The approach tends to produce the results when all distances become very similar i.e. the penalty is to severe to see the difference b/w the samples. The more general alternative will be to use some rescaling

$$\lambda := \min\{d_i\gamma : d_i \neq 0\}$$

where scaling constant $\gamma \in (0;1]$. The suggested choice is to use the median $\tilde{d}$ of all non-zero eigenvalues as a penalty:

$$\lambda := \tilde{d} = \text{median}\{d_i : d_i \neq 0\}.$$

Suggested Computation Algorithm

The algorithm has to be outlined as follows.

1. Standardize the matrix $\boldsymbol{X}$ for variance computation i.e. compute the differences $[\boldsymbol{X} - \bar{\boldsymbol{X}}_R]$ where $\bar{\boldsymbol{X}}_R$ is $n \times p$ dimensional matrix where every element is replaced by row average of the original matrix $\boldsymbol{X}$.

2. Compute singular value decomposition of $[\boldsymbol{X} - \bar{\boldsymbol{X}}_R]$. The decomposition has the form

$$[\boldsymbol{X} - \bar{\boldsymbol{X}}_R] = \boldsymbol{U}_c \boldsymbol{S}_c \boldsymbol{V}_c^T \tag{3}$$

   where subscript c emphasizes that is is for the *centered* matrix $[\boldsymbol{X} - \bar{\boldsymbol{X}}_R]$. The variance estimate has the form

$$\hat{\boldsymbol{\Sigma}} = \widehat{\text{Var}(\boldsymbol{X})} = \frac{1}{p-1}[\boldsymbol{X} - \bar{\boldsymbol{X}}_R][\boldsymbol{X} - \bar{\boldsymbol{X}}_R]^T$$

3. Define the penalty $\lambda := \min\{d_i\gamma : d_i \neq 0\}$ where γ is a user-defined penalty parameter and d_i-s are diagonal elements of $\boldsymbol{D}_c = \boldsymbol{S}_c \boldsymbol{S}_c^T$.

4. Compute the estimate of the penalized sigma $\hat{\boldsymbol{\Sigma}}_\lambda^{-1}$

$$\begin{aligned} \hat{\boldsymbol{\Sigma}}_\lambda^{-1} &= (p-1)\left[[\boldsymbol{X} - \bar{\boldsymbol{X}}_R][\boldsymbol{X} - \bar{\boldsymbol{X}}_R]^T + \lambda \boldsymbol{I}\right]^{-1} \\ &= (p-1)\,\boldsymbol{U}_c \left[\text{diag}\left[\frac{1}{d_1+\lambda}, \frac{1}{d_2+\lambda}, \dots, \frac{1}{d_n+\lambda}\right]\right] \boldsymbol{U}_c^T \end{aligned} \tag{4}$$

5. Compute the Mahalnobis distance using the penalized estimate (4). The Mahalanobis distance for individual columns $\boldsymbol{X}_1$ and $\boldsymbol{X}_2$ of $\boldsymbol{X}$ is denoted as $\hat{d}_M(\boldsymbol{X}_1, \boldsymbol{X}_2)$ and has

the form

$$\hat{d}_M(\boldsymbol{X}_1, \boldsymbol{X}_2) = \sqrt{[\boldsymbol{X}_1 - \boldsymbol{X}_2]^T \, \hat{\boldsymbol{\Sigma}}_\lambda^{-1} \, [\boldsymbol{X}_1 - \boldsymbol{X}_2]} \tag{5}$$
